# Particle Diffusivity and Free-Energy Profiles in Hydrogels from Time-Resolved Penetration Data

**DOI:** 10.1016/j.bpj.2020.12.020

**Published:** 2021-01-07

**Authors:** Amanuel Wolde-Kidan, Anna Herrmann, Albert Prause, Michael Gradzielski, Rainer Haag, Stephan Block, Roland R. Netz

**Affiliations:** 1 Fachbereich Physik, Freie Universität Berlin, Berlin, Germany; 2 Institut für Chemie und Biochemie, Freie Universität Berlin, Berlin, Germany; 3 Institut für Chemie, Technische Universität Berlin, Berlin, Germany

## Abstract

A combined experimental and theoretical method to simultaneously determine diffusivity and free-energy profiles of particles that penetrate into inhomogeneous hydrogel systems is presented. As the only input, arbitrarily normalized concentration profiles from fluorescence intensity data of labeled tracer particles for different penetration times are needed. The method is applied to dextran molecules of varying size that penetrate into hydrogels of polyethylene-glycol chains with different lengths that are covalently cross-linked by hyperbranched polyglycerol hubs. Extracted dextran bulk diffusivities agree well with fluorescence correlation spectroscopy data obtained separately. Empirical scaling laws for dextran diffusivities and free energies inside the hydrogel are identified as a function of the dextran mass. An elastic free-volume model that includes dextran as well as polyethylene-glycol linker flexibility quantitively describes the repulsive dextran-hydrogel interaction free energy, which is of steric origin, and furthermore suggests that the hydrogel mesh-size distribution is rather broad and particle penetration is dominated by large hydrogel pores. Particle penetration into hydrogels for steric particle-hydrogel interactions is thus suggested to be governed by an elastic size-filtering mechanism that involves the tail of the hydrogel pore-size distribution.

## Significance

The barrier function of mucus and other biological hydrogels against particles and pathogens depends on their diffusivity and free-energy profiles. We introduce a method that allows for simultaneous extraction of these quantities from non-normalized concentration profiles measured in penetration experiments. We apply our method to fluorescently labeled dextran polymers diffusing into polyethylene-glycol-based hydrogels and explain the results by an elastic free-volume model. We conclude that the penetration is governed by the large pores of the broad pore-size distribution, which is most likely a general characteristic of hydrogels. Our method is generally applicable to various kinds of labeled particles, including bacteria and virions, and can be used to help unravel the mechanisms behind mucus barrier function.

## Introduction

The penetration of particles into hydrogels is relevant for technological applications (1,2), drug delivery (3), and biological systems such as biofilms (4), the extracellular matrix (5), and mucus (6). Mucus, which is the most common biological hydrogel, lines the epithelial tissues of different organs, such as the respiratory, gastrointestinal, and urogenital tracts. Mucus is mainly composed of mucins, which are glycoproteins of varying length that absorb large amounts of water and thereby lend mucus its hydrogel nature, and additional components such as enzymes and ions (7). Mucins are relevant in the cell signaling context and presumably also play a role in the development of cancer (8). But primarily, mucus is a penetration barrier against pathogens, e.g., virions or bacteria, whereas it allows the permeation of many nonpathogens, e.g., nutrients, that are absorbed through the mucosa of the small intestine (9). Studies have suggested that based on the type of mucus, the combination of different mechanisms gives rise to the protective barrier function (10,11), in addition to the advective transport of pathogens through mucus shedding or clearance (12,13), which is not considered here. One typically distinguishes steric size-filtering mechanisms from interaction-filtering mechanisms (6,14); the latter presumably play a major role in the defense of organisms against pathogens because they allow for precise regulation of the passage of wanted and unwanted particles and molecules (15,16). Recent studies demonstrated that attractive electrostatic interactions reduce the particle diffusivity inside hydrogels substantially and much more than repulsive electrostatic interactions (17,18) and that salt concentration and the distribution of charges and pore sizes are important parameters that influence the permeation properties of charged hydrogels (19,20).

Particle penetration into mucus and biofilms has been studied by single-particle tracking techniques (21,22) as well as by methods in which a diffusor ensemble is observed (15,16,23,24). On short timescales, transient particle binding to the hydrogel (16, 17, 18) is important and leads to anomalous particle diffusion (25). On spatial length scales larger than the hydrogel mesh size and on timescales larger than typical binding escape times, particle diffusion is in a continuum description determined by the free-energy and diffusivity profiles across an inhomogeneous hydrogel system. In this framework, particle binding is effectively taken into account via a reduction of the diffusivity and a lowering of the free energy. If the free-energy and diffusivity profiles are known, particle penetration can be quantitatively predicted, provided the particle concentration is low and the particles do not modify the hydrogel properties in an irreversible manner. In this context, it should be noted that both profiles depend on the interactions between particle and hydrogel and therefore are different for each distinct hydrogel-particle pair. Because of method restrictions, experiments primarily focus on determining either the particle diffusivity inside the hydrogel (6,10,21) or on the partitioning between hydrogel and the bulk solution (26), from which the free energy inside the hydrogel (relative to the bulk solution) can be determined. However, for prediction of the penetration or permeation speed of particles into the hydrogel, both the diffusivity and the free energy in the hydrogel are needed.

**Figure 1:**
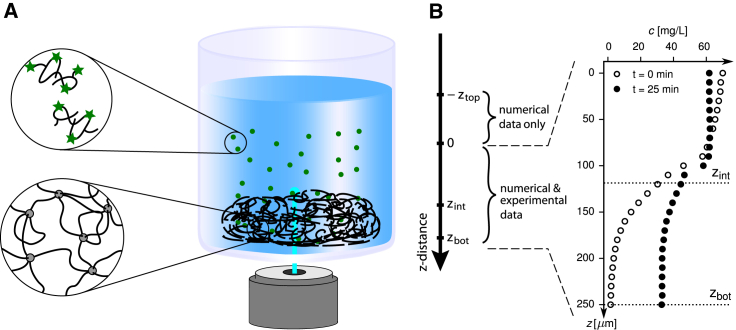
(*A*) Schematic drawing of the experimental setup. Concentration profiles of fluorescently labeled dextran molecules (*green*) are measured as they penetrate from the bulk solution (*blue*) into the hydrogel (*black*). The origin of the *z* axis is positioned such that experimentally measured profiles range from *z* = 0 to *z* = *z*_bot_. The hydrogel-bulk solution interface is located at *z* = *z*_int_. In the range from *z* = −*z*_top_ to *z* = 0, only numerically determined concentration profiles are available. (*B*) Exemplary experimental concentration profiles for two different penetration times for *M*_dex_ = 4 kDa dextran diffusing into the *hPG-G10* hydrogel are given; positions of the hydrogel-bulk solution interface *z*_int_ and the hydrogel-glass bottom interface *z*_bot_ are indicated. To see this figure in color, go online.

In this work, we study synthetic hydrogels that consist of polyethylene-glycol (PEG) linkers of different molecular masses that are permanently cross-linked by hyperbranched polyglycerol (hPG) hubs (2). Such synthetic hydrogels can be regarded as simple models for mucus because they display size-dependent particle permeabilities (14,27) similar to mucus. As diffusing particles, we employ fluorescently labeled dextran molecules of varying sizes. When using confocal laser-scanning fluorescence microscopy to investigate particle penetration into hydrogels, the sample can be oriented such that the hydrogel-bulk interface is either parallel (16) or perpendicular (28) to the optical axis, which makes no significant difference from a scanning perspective. However, for laterally extended samples like cell cultures that grow on a substrate, the parallel alignment causes the light path to span substantially larger distances, making this setup more prone to distortions in the imaging process. A perpendicular alignment, as employed in this work and sketched in Fig. 1, is therefore preferable for biological samples (28) and is also compatible with future extensions of such penetration assays to mucus-producing cell cultures.

We investigate the filtering function of hydrogels by theoretical analysis of time-resolved concentration profiles of the labeled dextran molecules as they penetrate into the hydrogel. The employed numerical method allows for simultaneous extraction of free-energy and diffusivity profiles from relative concentration profiles at different times and is a significant extension of earlier methods (29, 30, 31) because it does not require absolute concentration profiles but works with relative, i.e., arbitrarily normalized, concentrations. This is a crucial advantage because often fluorescence intensity profiles are subject to significant perturbation due to, e.g., laser light intensity fluctuations or fluorescence dye bleaching over the course of the experiment, and using relative concentrations makes the often-difficult conversion of measured intensity data into absolute particle concentrations obsolete. The analysis framework we introduce here can thus be used for a wide range of experimental setups to simultaneously extract free-energy and diffusivity profiles from a variety of different biological systems. As a check on the robustness of the method, the extracted dextran bulk diffusivities are shown to agree well with fluorescence correlation spectroscopy (FCS) data that are obtained separately. The obtained particle free energies and diffusivities inside the hydrogel are shown to obey empirical scaling laws as a function of the dextran mass. The dextran free energy inside the hydrogel is described by a free-volume model based on repulsive steric interactions between the dextran molecules and the hydrogel linkers, which includes dextran as well as hydrogel linker flexibility. This model constitutes a modified size-filtering mechanism for repulsive particle-hydrogel interactions, according to which particle penetration into hydrogel pores is assisted by the elastic widening of pores and the elastic shrinking of dextran molecules and matches the extracted particle free energies in the hydrogel quantitatively. The model furthermore suggests that the hydrogel mesh-size distribution is rather broad and that particle penetration is dominated by the fraction of large pores in the hydrogel.

## Materials and Methods

### Hydrogel preparation

The hydrogel is formed by cross-linking end-functionalized polyethylene-glycol-bicyclo[6.1.0]non-4-yne (PEG-BCN) linkers with hyperbranched polyglycerol azide (hPG-N_3_) hubs via strain-promoted azide-alkyne cycloaddition. The two macromonomers PEG-BCN and hPG-N_3_ are synthesized as previously described (2,32). The “click” reaction of binding the PEG-BCN linkers to the hPG-N_3_ hubs works in water, at room temperature, without the addition of a catalyst or external activation like heat or ultraviolet radiation and without the formation of byproducts. Two different sizes of PEG-BCN linkers are employed, having a molecular weight of either *M*_PEG_ = 6 or *M*_PEG_ = 10 kDa (for details about the mass distributions, see Supporting Materials and Methods, Section S1), the hydrogels are denoted as *hPG-G6* and *hPG-G10*, respectively. The number ratio of the PEG-BCN linkers to the hPG-N_3_ hubs (M_hPG_ = 3 kDa, 20% azide) is kept constant at 3:1 for both *hPG-G6* and *hPG-G10*. This ratio can ideally lead to a cubic lattice structure if each hPG-hub exactly binds to six PEG linkers. The chemical structure of the hPG-N_3_ hubs, however, allows on average for eight binding sites, making the hydrogel presumably quite disordered.

The two components of the hydrogel are stored as aqueous stock solutions at concentrations of 8.5 wt% (6 kDa PEG-BCN), 8.4 wt% (10 kDa PEG-BCN), and 5 wt% (hPG-N_3_). After very long storage times of the stock solutions of about 1 year, the cross-linking click reaction of PEG linkers and hPG hubs starts to become impaired, which is why storage times are kept short. To minimize aging effects of the hydrogels, hydrogel formation is always initiated shortly before the start of the experiments by mixing the components according to Table 1. The resulting gel solution is thoroughly vortexed before being placed as 1 *μ*L drops on the glass substrate. Both hydrogel solutions are adjusted to have the same mass concentration. However, after drying and reswelling on the glass substrate, volumes of the formed hydrogels are different and measured as $V_{\text{tot}}^{\text{hPG}-\text{G}6}$ = 0.42 ± 0.03 *μ*L and $V_{\text{tot}}^{\text{hPG}-\text{G}10}$ = 0.31 ± 0.04 *μ*L for *hPG-G6* and *hPG-G10*, respectively (see Fig. S1 in Supporting Materials and Methods, Section S2). This results in a final hydrogel concentration of 9 wt% (∼90 mg/mL) for *hPG-G6* and 12 wt% (∼120 mg/mL) for *hPG-G10*.

**Table 1:** Composition of the Hydrogels Used in this Study

	*n*_PEG_	$V_{\text{PEG}}^{\text{sol}}$^a^	*n*_hPG_	$V_{\text{hPG}}^{\text{sol}}$^b^	$V_{{\text{H}}_{2}\text{O}}$	$V_{\text{gel}}^{\text{sol}}$	*V*_app_	*m*_app_	*n*_app_
*hPG-G6*	142 nmol	10 *μ*L	47 nmol	2.8 *μ*L	13.0 *μ*L	25.8 *μ*L	1 *μ*L	38 *μ*g	7.3 nmol
*hPG-G10*	84 nmol	10 *μ*L	28 nmol	1.7 *μ*L	12.7 *μ*L	24.4 *μ*L	1 *μ*L	38 *μ*g	4.6 nmol

Here, $V_{\text{PEG}}^{\text{sol}}$ and $V_{\text{hPG}}^{\text{sol}}$ denote the volumes of the stock solutions, $V_{{\text{H}}_{2}\text{O}}$ is the volume of purified water added to the resulting gel solutions, and *n*_PEG_ and *n*_hPG_ denote the amount of PEG linkers and hPG hubs in the gel solutions. From the total resulting volume of the gel solutions $V_{\text{gel}}^{\text{sol}}$, only *V*_app_ = 1 *μ*L was placed as a gel spot on the glass substrate, leading to the combined applied amount *n*_app_ and the combined applied mass *m*_app_ of PEG linkers and hPG hubs.

a Solution is of 8.5 wt% for 6 kDa PEG and 8.4 wt% for 10 kDa PEG.

b hPG solution is of 5 wt%.

### Estimate of mean hydrogel mesh size

**Figure 2:**
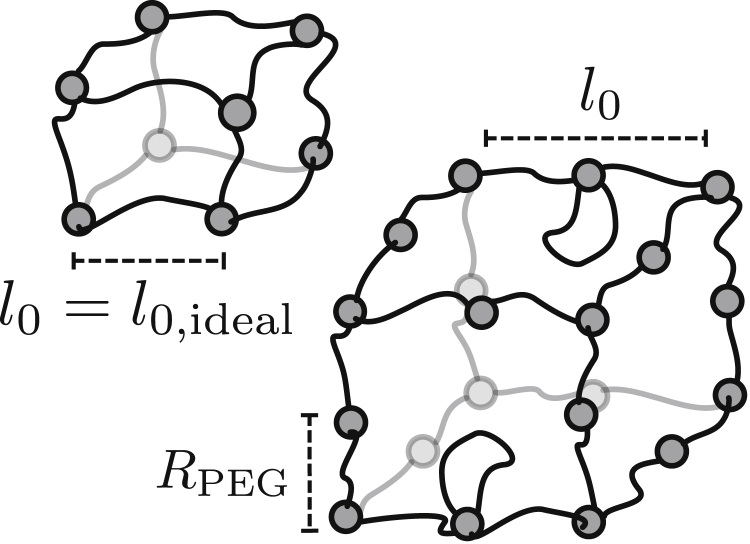
A cubic pore with lower connectivity to the right, containing two PEG linkers per edge instead of one, leads to an effectively larger unit-cell length *l*_0_ at the same PEG end-to-end distance *R*_PEG_. Only for a perfect cubic lattice to the left is the estimate of Eq. 1 valid and *l*_0_ = *l*_0,ideal_ = *R*_PEG_.

Assuming an idealized cubic hydrogel network structure, the mean mesh size can be easily estimated. The length of a cubic unit cell *l*_0,ideal_ follows from the total gel volume *V*_tot_ and the total number of hPG hubs $n_{\text{hPG}}^{\text{tot}}$ in mol as

(1) $$l_{0,\text{ideal}}=\sqrt[{3}]{\frac{V_{\text{tot}}}{n_{\text{hPG}}^{\text{tot}}N_{\text{A}}}},$$

where *N*_A_ is the Avogadro constant. The total volumes for the rehydrated gels are $V_{\text{tot}}^{\text{hPG}-\text{G}6}$ = 0.42 *μ*L and $V_{\text{tot}}^{\text{hPG}-\text{G}10}$ = 0.31 *μ*L as mentioned above. The total number of hPG hubs is given as $n_{\text{hPG}}^{\text{tot}}=n_{\text{hPG}}\times V_{\text{app}}/V_{\text{gel}}^{\text{sol}}$, with the values from Table 1 for the respective gel and in which we account for the fact that only *V*_app_ = 1 *μ*L of the total gel solution $V_{\text{gel}}^{\text{sol}}$ is applied onto the gel substrate. This results in rough estimates for the mesh size of $l_{0,\text{ideal}}^{\text{hPG}-\text{G}6}$ = 7.1 nm and $l_{0,\text{ideal}}^{\text{hPG}-\text{G}10}$ = 7.5 nm, which shows that even though PEG linkers of significantly different masses were used, the mesh sizes of the two gels differ only slightly. In deriving Eq. 1, one assumes an ideal hydrogel pore connectivity that corresponds to a perfect cubic lattice. There is no reason why the hydrogel should consist of a perfect cubic lattice; on the contrary, entropy favors a disordered network topology. For cubic pores with lower connectivity, Fig. 2 illustrates how the pore size *l*_0_ can increase for a fixed PEG end-to-end distance *R*_PEG_. Thus, except for the case of an ideal cubic lattice, the pore size *l*_0_ will be larger than the estimate of Eq. 1, as indeed suggested by our elastic free-volume model.

### Dextran preparation

Dextrans conjugated with the dye fluorescein isothiocyanate (FITC) are obtained from *Sigma-Aldrich* as d4-FITC, d10-FITC, d20-FITC, d40-FITC, and d70-FITC, the number stating the molecular weight in kDa of the commercial product. To remove unbound FITC from the dextran solutions, all batches are subjected to a desalting PD-10 column, which eliminates low-molecular weight compounds such as free FITC dye. This step is done according to the manufacturer’s recommendations, and the column is equilibrated using phosphate-buffered saline (PBS). Afterwards, the molecular weight distribution of all dextrans is determined by gel permeation chromatography (see Supporting Materials and Methods, Section S1).

### Penetration assay of FITC-labeled dextrans

After preparation of the hydrogel solutions and purification of the dextrans (see above), penetration assays are performed with five different dextran solutions and two different gels. For these assays, coverslips (Menzel #1; VWR, Darmstadt, Germany) with a diameter of 25 mm and a thickness of 0.13–0.16 mm are thoroughly washed with water and absolute ethanol and subsequently dried under a stream of nitrogen. For every experiment, 1 *μ*L of the respective hydrogel solution is placed on the center of the coverslip. The substrates with the applied gel spots are kept in a humid environment overnight, allowing hydrogel formation to be completed before the hydrogel spots are left to dry for 30 min at ambient conditions. Permeation experiments are performed within 1 day after hydrogel formation. To start a permeation experiment, a home-made polydimethylsiloxane stamp (1 × 1 cm) prepared with a cylindrical cavity in the middle (5 mm diameter) is placed on the coverslip, so that the dried hydrogel is located in the middle of the stamp’s cavity. The polydimethylsiloxane surrounding the dried hydrogel allows for the addition of solutions such as buffer or dextran. Before the measurement, 30 *μ*L of PBS buffer are added to reswell the hydrogel for 30 min, which typically creates hydrogel volumes of semispheroid shape with a base radius of 1050 *μ*m and heights of ∼150 *μ*m for *hPG-G10* and ∼210 *μ*m for *hPG-G6* (see Supporting Materials and Methods, Section S2). Afterwards, the coverslip is mounted on a Leica SP8 confocal laser-scanning microscope (CLSM; Leica, Wetzlar, Germany) and imaged using a 20× objective (0.75 HC PL APO water immersion objective with correction ring). In a first step, the hydrogel is visually identified by imaging the sample with a 488 nm laser and collecting the transmitted light using the transmission photomultiplier tube of the CLSM, allowing us to place the optical axis of the CLSM in the center of the hydrogel and to place the focal plane 30 *μ*m below the glass-hydrogel interface. After aligning the sample like this, the PBS buffer is removed from the cavity and replaced by 35 *μ*L of the FITC-dextran solution (0.07 mg/mL for all dextrans). This fixes the total length from the bottom of the glass dish at *z* = *z*_bot_ to the air-water interface at *z* = *z*_top_, where *z* = 0 corresponds to the end of the measurement region (see Fig. 1 *A*). The total length of the solution is thus *z*_tot_ = *z*_top_ + *z*_bot_ = 1780 *μ*m. The individual contributions to *z*_tot_ vary because of different gel thicknesses changing the extent of the measured region, ranging from *z* = 0 to *z* = *z*_bot_ (cf. also Fig. 1 *A*).

About 10 s after the application of the dextran solution, the spatial distribution of the FITC-based fluorescence intensity is measured using a z-stack that starts 30 *μ*m below and ends 410 *μ*m above the glass-hydrogel interface (with 10 *μ*m increments). The recorded intensities are afterwards truncated to probe the spatial FITC distribution within the hydrogel starting from the glass bottom (located at *z*_bot_) and extending ∼100 *μ*m into the bulk solution, away from the gel-water interface located at *z* = *z*_int_ (cf. Fig. 1 *A*). In these measurements, the sample is excited at *λ* = 488 nm, and the emission is recorded between 500 and 550 nm using a photomultiplier tube. For the *M*_dex_ = 4 kDa to the *M*_dex_ = 40 kDa dextrans, one z-stack is recorded every *Δt* = 10 s, yielding time-resolved FITC distributions after the penetration of the dextran molecules into the hydrogel network over time. For the *M*_dex_ = 70 kDa dextrans, a period of *Δt* = 30 s is used instead to account for the much smaller diffusion coefficient of the larger dextran molecules. The employed temporal resolutions can be easily estimated to be larger than timescales on which effects of anomalous diffusion are present; for diffusion over length scales larger than the mesh size of the hydrogel, normal diffusion is expected. An upper bound for the corresponding crossover timescale can be estimated as *τ* = $l_{0}^{2}$/*D*_gel_, where *l*_0_ = 24 nm is an upper estimate for the hydrogel mesh size and *D*_gel_ = 0.15 *μ*m^2^/s is the smallest obtained diffusion constant in the hydrogel (see below for explicit results). The resulting value of *τ* ≈ 0.2 ms, beyond which normal diffusion is expected, is several orders of magnitude lower than the experimental temporal resolution. Thus, anomalous diffusion cannot be observed in the experimental data, and the normal diffusion equation that is used to model the time-dependent experimental concentration profiles should be valid.

For all dextran types, measurements are performed at least three times with total measurement times of ∼30 min, with the exception of the *M*_dex_ = 70 kDa dextrans. Here, only one measurement is performed for each gel, but with a longer recording time of ∼1 h.

### FCS of FITC-labeled dextrans

Reference diffusion coefficients for the FITC-labeled dextran molecules in the bulk solution are obtained using FCS. The measurements are performed on a Leica TCS SP5 II CLSM with an FCS setup from PicoQuant (Berlin, Germany). The CLSM is equipped with an HCX PL APO 63×/1.20 W CORR CS water immersion objective. Samples are put on high-precision cover glasses (18 × 18 mm, 170 ± 5 *μ*m thick) and excited with the 488 nm Argon laser line. The fluorescent light is passed through a 50/50 beam splitter with a lower wavelength cutoff of *λ* = 515 nm. Both channels are detected separately with a single photon avalanche diode. Afterwards, a pseudo-cross correlation is performed between both channels to eliminate the influence of detector afterpulsing. Before a measurement, the optical setup is calibrated with the water-soluble Alexa-Fluor 488 dye. The correlated signal is fitted with two components and accounting for triplet states. The first component is fixed to a freely diffusing FITC dye molecule for which only the fraction is a fit parameter. The second component is set to a log-normal distributed species. The component fractions and means of distribution are fitted, and the width of distribution is taken from previously performed gel permeation chromatography measurements (for details about the fitting procedure, see Supporting Materials and Methods, Section S3). The fitted diffusion times are used to calculate the diffusion coefficients and hydrodynamic radii using the Stokes-Einstein relation.

### Numerical model and discretization

Extending a previously introduced method (29, 30, 31), spatially resolved diffusivity and free-energy profiles are estimated from experimentally measured concentration profiles. Numerical profiles are computed by discretizing the entire experimental setup from the glass bottom of the substrate to the air-water interface (*z*_bot_ to −*z*_top_ in Fig. 1 *A*). In the regime in which concentration profiles are measured (*z* = 0 to *z* = *z*_bot_), the experimental resolution is used as the numerical discretization width *Δz* = 10 *μ*m. For the range without experimental data (*z* = 0 to *z* = −*z*_top_), in total, six bins are employed. Two of those bins are spaced with *Δz* = 10 *μ*m; for the other four bins, discretization spacings between *Δz* = 300 and 400 *μ*m are used, depending on the z-length measured in the respective experiment *z*_bot_. The z-dimension of the total system is the same for all experiments and given as *z*_tot_ = *z*_top_ + *z*_bot_ = 1780 *μ*m. The experimentally measured region always extends from the glass bottom through the gel and at least 100 *μ*m into the bulk solution, away from the hydrogel-bulk interface, which leads to values of *z*_bot_ ≈ 300 *μ*m, depending on the exact thickness of the hydrogel in the respective measurement.

The numerical optimization problem is given by the cost function, which is defined as

(2) $$\sigma^{2}\left(D,F,\vec{f}\right):=\frac{1}{N\times M}\sum_{j=1}^{\text{N}}\sum_{i=1}^{\text{M}}{\left[c_{i}^{\text{num}}\left(t_{j}\right)-f_{\text{j}}\times c_{i}^{\text{exp}}\left(t_{j}\right)\right]}^{2},$$

with *N* the total number of experimental profiles, *M* the total number of experimental data points per concentration profile and *σ*^2^(*D*, *F*, $\vec{f}$) being the mean squared deviation between the experimental and numerical profiles. The diffusivity profile *D* = *D*(*z*), the free-energy landscape *F* = *F*(*z*), and the vector containing all scaling factors (see below for details) $\vec{f}$ = (*f*_1_, …, *f*_*j*_, …, *f*_*N*_) are all optimized to find the minimal value of *σ*^2^. This nonlinear regression is performed using the trust region method implemented in Python’s *scipy* package (33).

The numerical profiles

$${\vec{c}}_{\text{num}}\left(t_{\text{j}}\right)={\left(c_{1}^{\text{num}}\left(t_{\text{j}}\right),\ldots ,c_{i}^{\text{num}}\left(t_{\text{j}}\right),\ldots ,c_{M}^{\text{num}}\left(t_{\text{j}}\right)\right)}^{T}$$

are computed from the diffusivity and free-energy profiles as

(3) $${\vec{c}}_{\text{num}}\left(t_{\text{j}}\right)=e^{Wt_{\text{j}}}\times {\vec{c}}_{\text{init}},$$

where the rate matrix *W*(*D*, *F*) is defined as

$$W_{i,k}=\frac{D_{i}+D_{k}}{2\text{Δ}z^{2}}e^{-\frac{F_{i}-F_{k}}{2k_{\text{B}}T}},\;\text{with}\;k=i\pm 1$$

as explained previously (29). Numerical profiles at time *t*_*j*_ depend on the initial profile ${\vec{c}}_{\text{init}}$ at *t* = 0, which is determined as explained below.

The numerically computed profiles are fitted to the rescaled experimental profiles ${\vec{c}}_{\text{exp}}\left(t_{\text{j}}\right)$ at time *t*_*j*_ > 0. The scaling factors $\vec{f}$ are obtained simultaneously from the fitting procedure and correct drifts in the experimentally measured fluorescence intensity profiles (see Supporting Materials and Methods, Section S4). As a check, the numerical model is compared to the analytical solution for a model with piecewise constant values of the diffusivity and free energy in the respective regions. Results from the numerical model agree perfectly with those from the analytical solution (see Supporting Materials and Methods, Section S5).

### Construction of the initial concentration profile

**Figure 3:**
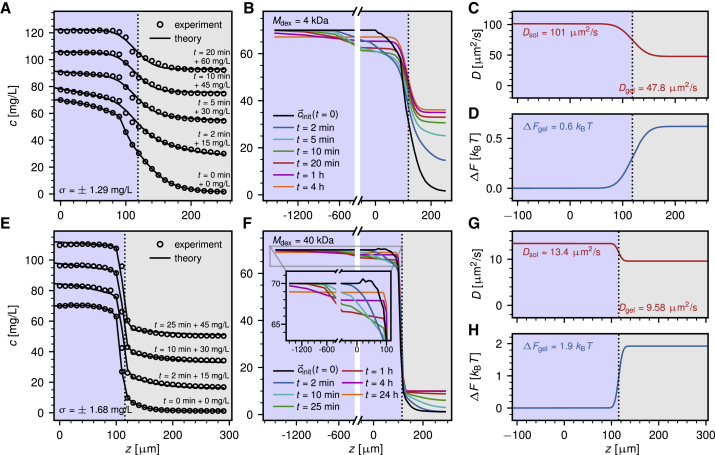
Exemplary time-dependent dextran concentration profiles from experimental measurements (*circles*) and numerical modeling (*solid lines*) for the *hPG-G10* hydrogel. Results for the smallest dextran with *M*_dex_ = 4 kDa in (*A*)–(*D*) are compared with results for *M*_dex_ = 40 kDa in (*E*)–(*H*). (*A* and *E*) Experimental and modeled concentration profiles agree very accurately; note that concentration profiles are shifted vertically for better visibility. The initial bulk concentration of dextran is *c*_0_ = 70 mg/L. (*B* and *F*) Modeled concentration profiles are presented for a wide range of penetration times. The initial profile ${\vec{c}}_{\text{init}}$ (*black line*) is based on experimental data (see Construction of the Initial Concentration Profile). (*C* and *G*) Extracted diffusivity profiles are given, showing that the diffusivity in the hydrogel is only slightly reduced compared to the bulk solution. (*D* and *H*) Extracted free-energy profiles are shown. Significant exclusion of dextran from the hydrogel is observed, with a stronger effect for the larger dextran. To see this figure in color, go online.

The initial profile ${\vec{c}}_{\text{init}}$, used for the computation of all later profiles according to Eq. 3, needs to cover the entire computational domain and is generated by extending the first experimentally measured profile ${\vec{c}}_{\text{exp}}$ (*t* = 0) into the bulk regime (from *z* = 0 to *z* = −*z*_top_, cf. Fig. 1 *A*). We define *t* = 0 as the time of the first measurement, which is performed ∼10 s after application of the dextran solution onto the gel-loaded substrate. For the spatial extension of the profile, a constant initial concentration is assumed in the bulk, the value of which is taken as the experimentally measured value furthest into the bulk *c*_0_ := $c_{1}^{\text{exp}}$ (*t* = 0) at *z* = 0. This leads to the following expression used for the initial profile

(4) $$c_{i}^{\text{init}}:=\left\{ \begin{array}{ll} c_{0}, & \text{if }-z_{\text{top}}\leq z_{i}\leq 0\\ c_{i}^{\text{exp}}\left(t=0\right), & \text{if }0<z_{i}\leq z_{\text{bot}} \end{array}\right.,$$

which by construction is continuous at *z* = 0. The initial profiles used for the fit procedure are shown in Fig. 3, *B* and *F* as black lines. To obtain concentration profiles in physical units, we set the first measured value furthest into the bulk equal to the applied dextran concentration *c*_0_ = 70 mg/L.

### Free-energy and diffusivity profiles

Because the experimental system consists of two regions, namely the hydrogel and the bulk solution, and to reduce the number of parameters of the numerical model to avoid overfitting, we employ sigmoidal profiles for the diffusivity *D*(*z*) and free energy *F*(*z*), which transition continuously from the value in the bulk solution to their values in the hydrogel. This sigmoidal shape is modeled using the following expressions:

$$D\left(z\right)=\frac{D_{\text{sol}}+D_{\text{gel}}}{2}+\frac{D_{\text{sol}}-D_{\text{gel}}}{2}\text{ erf}\left(\frac{z-z_{\text{int}}}{\sqrt{2}d_{\text{int}}}\right),$$

(5) $$F\left(z\right)=\frac{\Delta F_{\text{gel}}}{2}+\frac{\Delta F_{\text{gel}}}{2}\text{ erf}\left(\frac{z-z_{\text{int}}}{\sqrt{2}d_{\text{int}}}\right),$$

where erf(*z*) := 1/$\sqrt{\pi }\int_{-z}^{z}e^{-z^{\prime 2}}$ *dz*′ is the error function. The fit parameters *z*_int_ and *d*_int_ determine the transition position and width, respectively, and are the same for the free-energy and diffusivity profiles. Because only free-energy differences carry physical meaning, the free energy in the bulk solution is set to zero so that *F*_sol_ = 0. The values of the diffusivity and free energy in the hydrogel and in the bulk solution are thus determined by fitting the five parameters of Eq. 5, namely *D*_gel_, *ΔF*_gel_, *D*_sol_, *z*_int_, and *d*_int_, to the experimentally measured concentration profiles.

**Figure 5:**
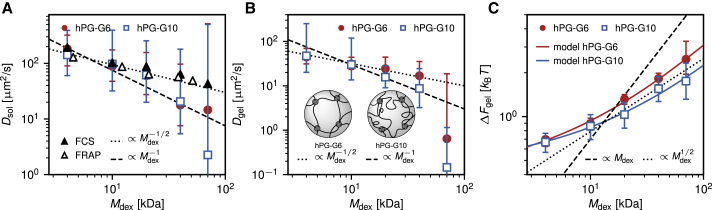
Results for the diffusivity and free energy obtained from the experimental measurements as a function of dextran mass. (*A*) Fitted diffusivities in the bulk solution (*squares* and *circles*) agree within the error with FCS data measured in this work (*solid black triangles*) and with FRAP measurements from literature (34) (*open black triangles*). (*B*) Fitted diffusivities in the hydrogel are reduced relative to the bulk values and are compared to different power laws. (*C*) Dextran molecules are excluded from the hydrogel and *ΔF*_gel_ > 0 for all dextran masses. For larger dextran molecules, *ΔF*_gel_ increases as a square root with the dextran mass. The results from the free-volume model of Eq. 12 (*continuous lines*) agree nicely with the measurements. Error bars have been estimated as explained in Supporting Materials and Methods, Section S6. The inset in (*B*) presents a schematic depiction of the two different gels. Even though the *hPG-G10* gel is composed of larger linkers, the mass density is larger than in the *hPG-G6* gel, which results in an effectively smaller pore size. To see this figure in color, go online.

Confidence intervals for the obtained parameters of *D*_sol_, *D*_gel_, and *ΔF*_gel_ are estimated by determining the parameter values that change *σ* by not more than 50% (for details, see Supporting Materials and Methods, Section S6). The error bars shown in Fig. 5 are then obtained by averaging the confidence intervals over all measurements.

## Results and Discussion

Fluorescence intensity profiles of FITC-labeled dextran molecules penetrating into PEG-based hydrogels are analyzed using the procedure explained in the Materials and Methods. The analysis is based on numerical solutions of the one-dimensional generalized diffusion equation (35)

(6) $$\frac{\partial c\left(z,t\right)}{\partial t}=\frac{\partial }{\partial z}\left[D\left(z\right)e^{-\beta F\left(z\right)}\frac{\partial }{\partial z}\left(c\left(z,t\right)e^{\beta F\left(z\right)}\right)\right],$$

where *c*(*z*, *t*) is the concentration at time *t* and depth *z* (see Fig. 1), *D*(*z*) and *F*(*z*) are the spatially resolved diffusivity and free-energy profiles that the dextran molecules experience, and *β* = 1/*k*_B_*T* is the inverse thermal energy. Whereas the diffusivity *D*(*z*) describes the mobility of dextran molecules at position *z*, the free-energy profile *F*(*z*) uniquely determines the equilibrium partitioning of dextran molecules. The numerical solution of Eq. 6 provides a complete model of the penetration process into the hydrogel and at the same time allows for extraction of the diffusivity and free-energy profiles by comparison with experimentally measured concentration profiles. A direct conversion of measured fluorescence intensities into absolute concentrations is often difficult because of drifts of various kinds. The method developed here circumvents this problem and allows for in-depth analysis of arbitrarily normalized concentration profiles, as explained in Numerical Model and Discretization. Complete profiles of free energies and diffusivities, both in the bulk and in the PEG hydrogel, are obtained, and the results for different hydrogels and dextran molecules of varying sizes will be analyzed in the following.

### Comparison between experimental and modeled concentration profiles

Fig. 3, *A* and *E* shows exemplary concentration profiles for dextran molecules with molecular masses of *M*_dex_ = 4 kDa and *M*_dex_ = 40 kDa penetrating into the *hPG-G10* hydrogel (see Hydrogel Preparation). Measurements are performed over a total time span of ∼30 min, and concentration profiles are recorded every 10 s, leading to a total of ∼180 concentration profiles as input for the numerical extraction of the diffusivity and free-energy profiles. The first measured concentration profile at *t* = 0 min represents the start of the experiment, ∼10 s after the dextran solution was applied onto the gel (see Penetration Assay of FITC-Labeled Dextrans). The numerically determined concentration profiles (*lines*) reproduce the experimental data (data points) very accurately, as seen in Fig. 3, *A* and *E*. The deviation is estimated from the normalized sum of residuals, *σ* (according to Eq. 2), which is below 2 mg/L for both measurements. A stationary concentration profile is obtained in the theoretical model only after 4 h of penetration for the smaller 4 kDa dextran (see Fig. 3 *B*); for the larger dextran molecule, the stationary profile is reached only after an entire day (see Fig. 3 *F*). These times significantly exceed the duration of the experiments.

The extracted diffusivity and free-energy profiles in Fig. 3, *C*, *D*, *G*, and *H* reveal the selective hydrogel permeability for dextran molecules of varying size. The free-energy difference in the hydrogel is positive *ΔF*_gel_ > 0 for both dextran sizes, indicating that dextran is repelled from the hydrogel. The dextran partition coefficient *K*_gel_ between the hydrogel and the bulk solution is related to the change in the free energy *ΔF*_gel_ as

(7) $$K_{\text{gel}}=e^{-\beta \text{Δ}F_{\text{gel}}}.$$

According to Eq. 7, the obtained free-energy differences *ΔF*_gel_ = 0.6 *k*_B_*T* and *ΔF*_gel_ = 1.9 *k*_B_*T* correspond to partition coefficients of about *K*_gel_ ≈ 1/2 and *K*_gel_ ≈ 1/7 for the smaller and the larger dextran molecules, respectively, which illustrates a significant exclusion in particular for the larger dextran. Compared with the partition coefficients, the diffusion constants in the hydrogel decrease only slightly as a function of the dextran mass. This suggests that the dextran molecules are only modestly hindered in their motion, a conclusion that will be rationalized by our elastic free-volume model further below.

**Figure 4:**
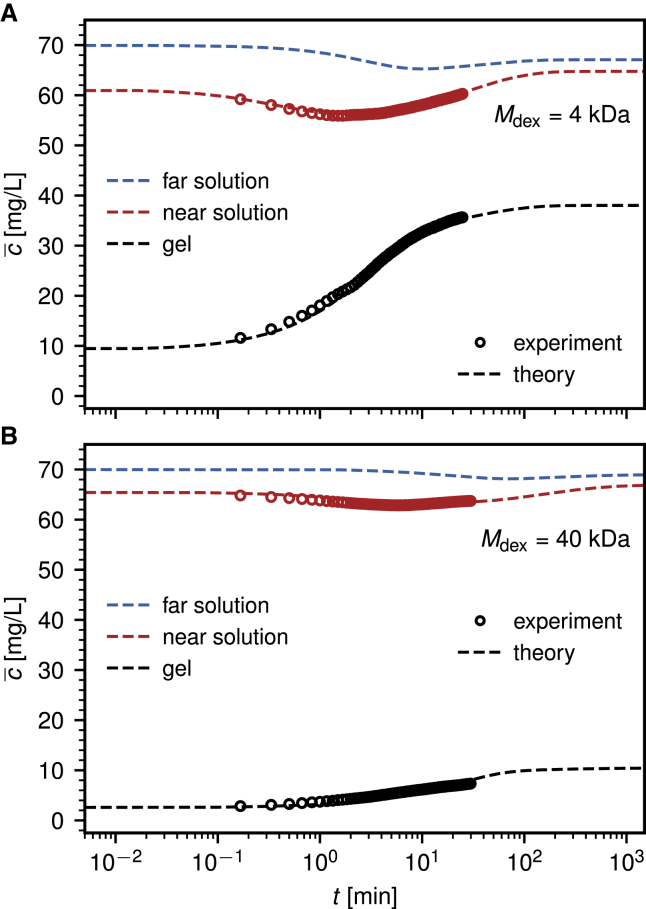
Comparison of experimental results (*circles*) and modeling results based on the extracted diffusivity and free-energy profiles (*lines*) for the mean dextran concentration $\bar{c}$ over time in three different regions, the far solution (−*z*_top_ < *z* < 0), the near solution (0 < *z* < *z*_int_), and the gel (*z*_int_ < *z* < *z*_bot_); see Fig. 1. The systems are the same as shown in Fig. 3. A nonmonotonic dextran concentration is measured over time in the near and far solution regions. The fact that $\bar{c}$ in the gel does not vanish for *t* → 0 reflects that the first measurement at *t* = 0 is done ∼10 s after the application of the dextran solution onto the gel. The initially employed bulk dextran concentration is *c*_0_ = 70 mg/L. To see this figure in color, go online.

Fig. 4 shows the temporal evolution of the average dextran concentration $\bar{c}$ in three different regions, namely inside the gel for *z*_int_ < *z* < *z*_bot_, in the near solution for 0 < *z* < *z*_int_, and in the far solution for −*z*_top_ < *z* < 0 for the same data shown in Fig. 3. The lines show the predictions based on the extracted diffusivity and free-energy profiles and the circles the experimental data, which are not available in the far solution range. The average concentration in the gel (*black*) increases monotonically and saturates after about 1 h for both dextran sizes. Note that the stationary final concentration in the hydrogel is considerably less for the larger dextran with *M*_dex_ = 40 kDa. In contrast, the average concentration in the far solution saturates more slowly and shows a slight nonmonotonicity for both dextran masses (*blue*). This nonmonotonicity is more pronounced in the near solution (*red*) and is caused by the fact that dextran molecules diffuse quickly into the hydrogel from the near solution in the beginning of the experiment, whereas the replenishment from the bulk solution takes a certain time, as also seen in the concentration profiles in Fig. 3, *B* and *F*. Very good agreement between experiments and modeling results is observed.

### Influence of dextran size on hydrogel penetration

The same analysis is performed for dextran molecules of molecular masses ranging from *M*_dex_ = 4 kDa to *M*_dex_ = 70 kDa that penetrate into PEG hydrogels with two different linker lengths, namely *hPG-G6* with a PEG linker size of *M*_PEG_ = 6 kDa and *hPG-G10* with *M*_PEG_ = 10 kDa. Fig. 5 shows the extracted diffusivities and free energies, which result from averages over at least three experiments for each system, except for *M*_dex_ = 70 kDa dextran, for which only one experiment was performed.

Fig. 5 *A* shows the bulk diffusivities *D*_sol_ extracted from measured concentration profiles as colored symbols; in principle, there should be no difference between results for *hPG-G6* and *hPG-G10*. A power-law relation between the dextran mass and the diffusivity according to *D*_sol_ ∝ $M_{\text{dex}}^{-\nu }$ is shown as straight lines for *ν* = 1 (*broken line*) and for *ν* = 1/2 (*dotted line*). An exponent of *ν* = 1/2 agrees nicely with our FCS data (*solid black triangles*; see FCS of FITC-Labeled Dextrans) as well as with literature fluorescence recovery after photobleaching (FRAP) measurements (34) (*open black triangles*). The value *ν* = 1/2 follows from combining the generally applicable Stokes-Einstein relation *D*_sol_ = *k*_B_*T*/6*πη*_w_*r*_0_ (36) with the scaling of the dextran hydrodynamic radius according to *r*_0_ ∝ $M_{\text{dex}}^{\nu }$ (37,38) by assuming that the bulk solution is a theta solvent for dextran polymers (39,40) (see Supporting Materials and Methods, Section S7 for details). The exponent *ν* = 1/2 is only expected for linear polymers, whereas dextran is in fact a branched polymer. The good agreement of FCS and FRAP data with the power law for *ν* = 1/2 suggests that the degree of branching is low (41) or that branching effectively compensates self-avoidance effects. The dextran hydrodynamic radii estimated from the FCS measurements compare well with the values reported by the supplier (see Table 2). The data for *D*_sol_ obtained from the time-dependent dextran concentration profiles show rather large uncertainties, which is due to the fact that the concentration profiles are rather insensitive to the bulk diffusivities; they are within error bars consistent with our FCS results but do not allow extraction of the power-law scaling with any reasonable confidence.

**Table 2:** Dextran Radii

*M*_dex_	*r*_0_	*r*_FCS_
4 kDa	1.4 nm	1.5 nm
10 kDa	2.3 nm	2.7 nm
20 kDa	3.3 nm	3.2 nm
40 kDa	4.5 nm	4.3 nm
70 kDa	6.0 nm	6.4 nm

Hydrodynamic radius *r_0_* as reported by the supplier, in comparison to estimated hydrodynamic radius *r_FCS_* based on our FCS measurements using the Stokes-Einstein relation and the viscosity of water as *η_w_* = 0.8 ×10^−3^ Pa s.

Values for the diffusion constant in the hydrogel *D*_gel_ are compared with power laws with exponents *ν* = 1/2 and *ν* =1 in Fig. 5 *B*. The difference of the diffusion constants between the two different hydrogels is within the error bars, which reflects the fact that the estimated mean hydrogel mesh sizes, using a very simplistic hydrogel network model with a perfect cubic structure, are $l_{0,\text{ideal}}^{\text{hPG}-\text{G}6}$ = 7.1 nm and $l_{0,\text{ideal}}^{\text{hPG}-\text{G}10}$ = 7. 5nm (see Estimate of Mean Hydrogel Mesh Size) and thus quite similar to each other. It is to be noted that for *M*_dex_ ≤ 20 kDa, the estimated mesh sizes are larger than twice the dextran hydrodynamic radii from Table 2, which would not suggest any dramatic confinement effect on the diffusion constant (42). Interestingly, for the data for which *M*_dex_ ≳ 20 kDa, the hydrogel with the larger linker length (*hPG-G10*), which has a slightly higher mesh size, is seen to reduce the diffusion constant slightly more, which at first sight is counterintuitive. This finding can be rationalized by the fact that the *hPG-G10* gel has a higher mass density compared to the *hPG-G6* gel (see Hydrogel Preparation), and thus, the effective pore size is presumably substantially smaller. This is schematically illustrated in the inset in Fig. 5 *B*. A diffusivity scaling with an exponent *ν* = 1, which describes the data for *hPG-G10* slightly better, could be rationalized by screened hydrodynamic interactions or by reptation-like diffusion (43). In fact, a crossover in the scaling of the diffusivity with increasing hydrogel density from *ν* = 1/2 to *ν* = 1 has been described before for dextran penetrating into hydroxypropyl cellulose (38). However, because of the large error bars, extraction of the diffusivity scaling with respect to dextran mass in the two gels is not uniquely possible. This is mostly due to the fact that the diffusivities change rather mildly with varying dextran mass. This is why we do not attempt to model the scaling of the extracted diffusivities, as was done elsewhere before (18,19,44), but rather focus on the mechanism behind the extracted free-energy differences in the following.

Fig. 5 *C* shows the extracted values of *ΔF*_gel_ for the two hydrogels as a function of the dextran mass. In all measurements, we find *ΔF*_gel_ > 0, which suggests exclusion of the dextran molecules from the hydrogel. Also, the value of *ΔF*_gel_ increases with the dextran mass. Because dextran, as well as the PEG-hPG based hydrogels, is uncharged (45), this exclusion must be due to steric repulsion, possibly enhanced by hydration repulsion (46,47).

### Elastic free-volume model for dextran penetration in hydrogels

For the larger dextran molecules, the hydrogel with the smaller PEG linkers, *hPG-G6*, displays a slightly stronger exclusion. The power-law relation between the hydrogel free energy and dextran mass according to *ΔF*_gel_ ∝ $M_{\text{dex}}^{\alpha }$ with an exponent of *α* = 1/2 describes the data well for larger dextran masses *M*_dex_ ≳ 20 kDa, as shown by the dotted black line in Fig. 5 *C*. This power-law behavior is in fact compatible with a simplistic elastic free-volume model for the penetration of dextran molecules into hydrogels, which yields the solid lines and will be derived in the following.

**Figure 6:**
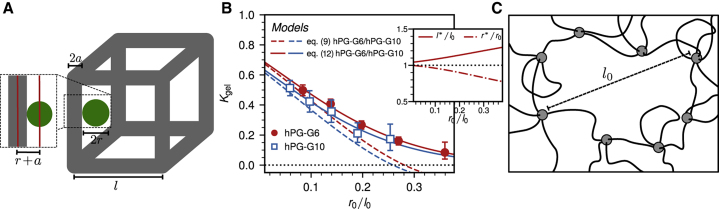
Elastic free-volume model for the partitioning of a particle in a hydrogel. (*A*) Schematic sketch of the cubic unit-cell model for the hydrogel is given, made up of connected linkers of length *l* and a finite radius of *a*. The diffusing particle is modeled as a sphere of radius *r*. Both the particle and the linkers are elastic and can stretch or contract. (*B*) Partition coefficient *K*_gel_ extracted from the experimentally measured dextran concentration profiles (*symbols*) is shown in comparison with the elastic free-volume model predictions according to Eq. 12 (*solid lines*). The results of the nonelastic model according to Eq. 9 are shown as dashed lines. Error bars have been estimated as explained in Supporting Materials and Methods, Section S6. The inset shows the equilibrium values of *l*^∗^ and *r*^∗^ obtained for the *hPG-G6* gel. (*C*) Illustration of a disordered pore in the hydrogel that has a mesh size *l*_0_ and consists of more than four linkers is given (see also Fig. 2). To see this figure in color, go online.

The model geometry is sketched in Fig. 6 *A* and consists of a single dextran molecule of radius *r* (*green sphere*) inside a cubic unit cell of the PEG-based hydrogel (*gray cylinders*), similar to previous coarse-grained hydrogel models (18, 19, 20). The presence of the hPG hubs connecting the PEG linkers is neglected in the following. The dextran experiences a reduction of its free volume compared with the bulk solution because of steric interactions with the PEG linkers. In the simple model geometry, the PEG linkers are located at the edges of the cubic unit cell and are modeled as impenetrable cylinders of radius *a* and length *l*. Conformational fluctuations of the PEG linkers are not treated explicitly in this model; instead, the linker length *l* and radius *a* are to be understood as average values over different confirmations of the linker chains. The excluded volume *V*_ex_ for dextran in the cubic unit cell consists of a quarter of each of the 12 cylinders at the edges. The accessible or free volume in the hydrogel *V*_free_ depends on the sum of sphere radius *r* and cylinder radius *a* and is given by

(8) $$\begin{array}{l} V_{\text{free}}=V_{\text{unit}}-V_{\text{ex}}\\ \;=l^{3}-\frac{12}{4}\pi {\left(r+a\right)}^{2}l+2V_{\text{cyl}}. \end{array}$$

Here, *V*_unit_ = *l*^3^ is the volume of the unit cell and *V*_cyt_ = (16/3)(*r* + *a*)^3^ is the volume of two intersecting cylinders (48), which is subtracted from the excluded volume to avoid over counting of the unit-cell corners. The entropic contribution to the total free energy is given by

(9) $$\begin{array}{l} \Delta F_{\text{vol}}=-k_{\text{B}}T\;\text{ln}\left(\frac{V_{\text{free}}}{V_{\text{unit}}}\right)\\ \;=-k_{\text{B}}T\;\text{ln}\left(1-3\pi {\left[\frac{r+a}{l}\right]}^{2}+\frac{32}{3}{\left[\frac{r+a}{l}\right]}^{3}\right). \end{array}$$

Because dextran and the PEG linkers are elastic polymers, they are both flexible and can deform. For small deformations, the polymers behave like Gaussian chains (39,40). The elastic deformation free energy for a cubic unit cell consisting of 12 equally deformed PEG linkers can be written as (for a detailed derivation, see Supporting Materials and Methods, Section S8)

(10) $$\Delta F_{\text{PEG}}=\frac{12}{2}k_{\text{B}}T\left({\left[\frac{l}{l_{0}}\right]}^{2}+\frac{1-4{\left[\frac{l}{l_{0}}\right]}^{2}}{2+{\left[\frac{l}{l_{0}}\right]}^{2}}\right).$$

Here, *l*/*l*_0_ is the relative stretching of the PEG linkers, where *l*_0_ denotes the edge length of the unit cell in the absence of dextran molecules. The elastic deformation energy of dextran is obtained in the same fashion and reads

(11) $$\Delta F_{\text{dex}}=\frac{3}{2}k_{\text{B}}T\left({\left[\frac{r}{r_{0}}\right]}^{2}+{\left[\frac{r_{0}}{r}\right]}^{2}-2\right),$$

where *r* denotes the deformed dextran radius and the unperturbed dextran radius is denoted by *r*_0_ and is taken from Table 2. The complete free energy follows as

(12) $$\Delta F_{\text{gel}}\left(r,l\right)=\Delta F_{\text{vol}}\left(r,l\right)+\Delta F_{\text{PEG}}\left(l\right)+\Delta F_{\text{dex}}\left(r\right).$$

The equilibrium free energy is given by the minimal value of this free-energy expression, obtained for the optimally stretched unit-cell length *l*^∗^ and the optimal dextran radius *r*^∗^, which are determined numerically. The values of the unit-cell length *l*_0_ and the PEG linker thickness *a* are adjusted by fits to the experimental data. The model results are shown in Fig. 6 *B* in terms of the partition coefficient as solid lines and compared with the experiments (*circles* and *squares*) as a function of the length ratio *r*_0_/*l*_0_. The inset shows the obtained equilibrium values for *l*^∗^ and *r*^∗^ for the *hPG-G6* gel. A considerable stretching of PEG linkers and compression of dextran are observed, which shows that elasticity effects of both PEG linkers and dextran molecules are important and cannot be neglected when estimating the free volume.

The fit to the experimental data yields $l_{0}^{\text{hPG}-\text{G}6}$ = 16.7 nm, $l_{0}^{\text{hPG}-\text{G}10}$ = 23.7 nm, *a*_hPG-G6_ = 3.4 nm, and *a*_hPG-G10_ = 5.4 nm. The fit values of *a* certainly represent an effective PEG linker radius and include the layer of tightly bound hydration water. They are indeed, close to the respective equilibrium PEG radii *R*_PEG_ = *b*_fl_ $N_{\text{PEG}}^{3/5}/\sqrt{3}$, given as $R_{\text{PEG}}^{\text{hPG}-\text{G}6}$ = 4.4 nm and $R_{\text{PEG}}^{\text{hPG}-\text{G}10}$ = 5.99 nm, where *b*_fl_ = 0.4 nm denotes the Flory monomer length (49) and *N*_PEG_ is the respective number of PEG monomers. In fact, the free-volume model yields estimates of the number of hydration waters per PEG monomer that scatter around 8, in rough agreement with literature values (see Fig. S8; Supporting Materials and Methods, Section S9).

The fit values for the unit-cell length *l*_0_ are significantly larger than the mean mesh size estimated based on Eq. 1, which for a perfectly ordered cubic lattice predicts $l_{0,\text{ideal}}^{\text{hPG}-\text{G}6}$ = 7.1 nm and $l_{0,\text{ideal}}^{\text{hPG}-\text{G}10}$ = 7.5 nm, but still considerably shorter than the PEG contour lengths *L* = $b_{0}^{\text{PEG}}$ *N*_PEG_, which are *L*_hPG-G6_ = 48.5 nm and *L*_hPG-G10_ = 80.9 nm, where $b_{0}^{\text{PEG}}$ = 0.356 nm is the PEG monomer length (49). Although the large unit-cell lengths obtained from the fit to the elastic free-volume model could reflect a substantial stretching of individual PEG polymers, there is no a priori reason why the linkers should be stretched to such a considerable fraction of their contour length. We therefore rationalize this surprising result in terms of a broad distribution of pore sizes that exhibit different topologies. To illustrate this, a random pore is schematically shown in Fig. 6 *C*. Based on the 3:1 number ratio of linkers/cross-linkers in the hydrogel formulation (cf. Hydrogel Preparation and Fig. 2), a perfectly cubic lattice could form, in which each hub is connected to six different linkers. Such an ideal cubic connectivity is, of course, entropically highly unfavorable, and the connectivity distribution of hubs, i.e., the distribution of the number of linkers that connect to one hub, will be rather broad and the network topology disordered, in which case the PEG end-to-end distance *R*_PEG_ will be significantly smaller than the pore size *l*_0_ (cf. also Estimate of Mean Hydrogel Mesh Size). Whereas in a cubic lattice, each cubic facet consists of four hubs and four linkers, the pores present in the actual hydrogel will show a broad distribution of the number of participating linkers. For illustration, the pore shown in Fig. 6 *C* consists of eight linkers. Clearly, dextran molecules will tend to be located in larger pores to maximize their free volume, and therefore, the fit parameters of our model will be dominated by the tail of the pore-size distribution, which explains the large fit values for *l*_0_. This finding also allows us to rationalize the larger extracted free energy in the hydrogel in the case of the *hPG-G6* gel, even though the *hPG-G10* gel mass density is higher (cf. Fig. 5 *C*). The tail of the pore-size distribution of the *hPG-G10* gel presumably contains larger pores that can stretch even further to minimize the unfavorable dextran-PEG interactions. Clearly, the precise topology and compositional distribution of pores cannot be predicted by our analysis; our results should thus be merely interpreted as an indication of the presence of large pores and a disordered network topology.

An approximate nonelastic version of the free-volume model is obtained by neglecting the polymer deformation term and just keeping the excluded volume term, Eq. 9, which becomes accurate in the limit of *l*_0_ ≫ *r*_0_, where *r*^∗^ ≈ *r*_0_ and *l*^∗^ ≈ *l*_0_. These approximate results are shown as broken lines in Fig. 6 *B* and describe the experimental data only for small values of *r*_0_/*l*_0_. When additionally approximating the logarithm in Eq. 9, the obtained expression for the free energy is similar to results derived for a random-fiber network (50). Our free-volume model is valid only for short-ranged steric and hydration repulsive interactions between diffusor and linkers; if long-ranged and, in particular, attractive interactions are present—for example, electrostatic interactions for low salt concentrations—the model would need to be adjusted accordingly.

### Derivation of particle permeabilities through hydrogel barriers

Permeation through biological barriers is quantified by the permeability coefficient *P*, which is defined as (51)

(13) $$P\left(z_{1},z_{2}\right)=\frac{J}{c\left(z_{1}\right)-c\left(z_{2}\right)},$$

where *c*(*z*_1_) and *c*(*z*_2_) are the particle concentrations at the two sides *z*_1_ and *z*_2_ of the barrier and *J* denotes the particle flux through the barrier. Based on the diffusion equation (Eq. 6), the inverse permeability can be written as (for a detailed derivation, see Supporting Materials and Methods, Section S10)

(14) $$\frac{1}{P\left(z_{1},z_{2}\right)}=\int_{z_{1}}^{z_{2}}\frac{e^{\beta F\left(z\right)}}{D\left(z\right)}dz.$$

For a step-like barrier, one obtains

(15) $$\frac{1}{P}=\frac{e^{\beta \text{Δ}F_{\text{gel}}}}{D_{\text{gel}}}L.$$

Here, Δ*F*_gel_ and *D*_gel_ are the particle free energy relative to the solution and the diffusivity inside the hydrogel, and *L* denotes the width of the hydrogel barrier.

**Figure 7:**
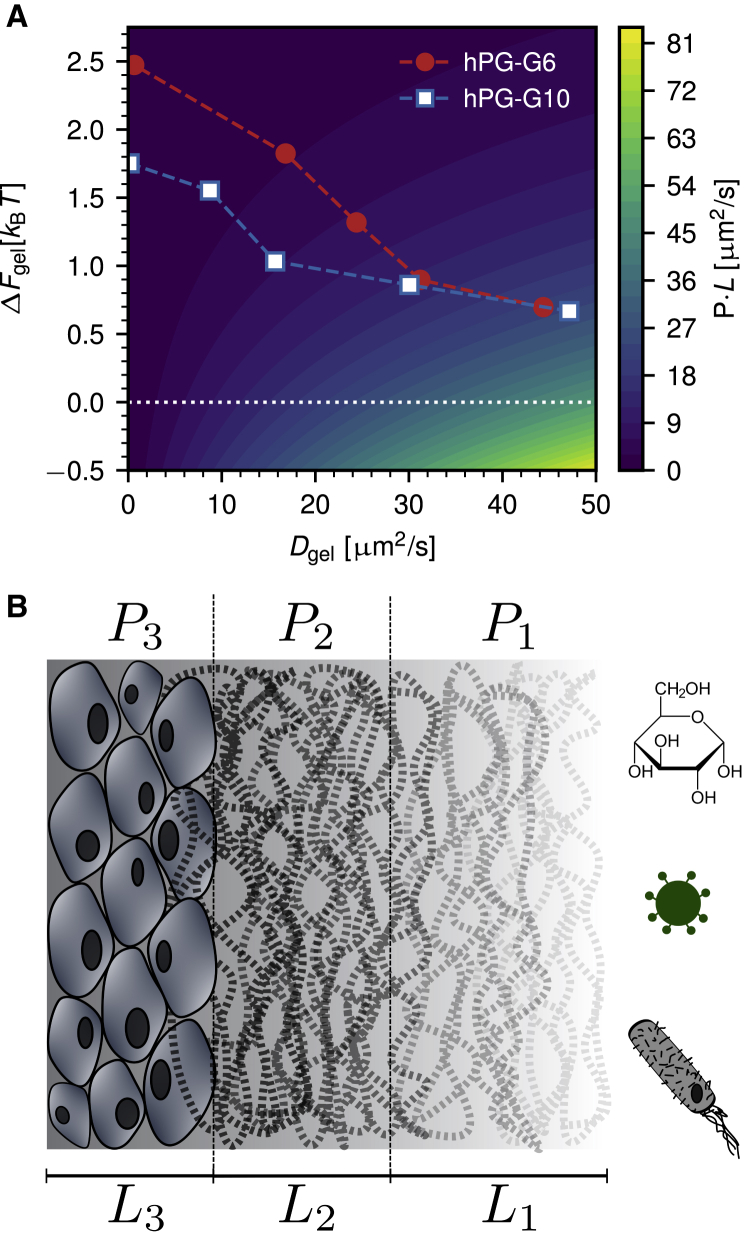
(*A*) Normalized permeability coefficient *PL* through a single box-like hydrogel barrier of width *L* as a function of the hydrogel free energy *ΔF*_gel_ and the hydrogel diffusivity *D*_gel_ from Eq. 15. High permeability is observed for low free-energy barriers and high diffusivities in the hydrogel. The symbols denote the experimental data from Fig. 5. Because of opposing trends in the free-energy barrier and the diffusivity, both hydrogels display comparable permeability coefficients. (*B*) Schematic layered structure of a mucous membrane, as found in the stomach, is given. Examples for different diffusors are shown, including nutrients such as glucose and pathogens such as virions or bacteria. The diffusors have to penetrate different layers of varying permeabilities to enter the tissue below the mucous membranes, the total permeability of a layered structure follows from Eq. 16. To see this figure in color, go online.

Fig. 7 *A* shows normalized permeability coefficients *PL* for a single step-like barrier according to Eq. 15, which are independent of the thickness of the barrier *L*, as a function of the gel free energy and the gel diffusivity. The values extracted from the experimental data for different dextran molecules in the two gels from Fig. 5 are indicated by data points. Obviously, the highest permeability is observed for a low free-energy barrier and a high particle diffusivity, as is the case for the smallest dextran molecules (*lower right corner* in Fig. 7 *A*). On the other hand, permeation is hindered by either a high free-energy barrier or a low diffusivity in the hydrogel, both of which are observed for dextran molecules with larger molecular weights. Because of counterbalancing effects of stronger exclusion from the *hPG-G6* gel and increased immobilization in the case of *hPG-G10*, both hydrogels display comparable permeability coefficients for the chosen dextran molecular masses.

## Conclusions

The method introduced in this work allows for the simultaneous extraction of diffusivity and free-energy profiles of particles that permeate into spatially inhomogeneous hydrogel systems; we demonstrate the method using concentration profile measurements of fluorescently labeled dextran molecules permeating into PEG-hPG-based hydrogels. The advantage over alternative methods is that both diffusivity and free-energy profiles are obtained from a single experimental setup. This is important because only the combination of diffusivity and free-energy profiles completely determines the diffusion of particles.

The extracted diffusivities and free energies are analyzed in terms of empirical scaling laws as a function of the dextran mass, and a modified elastic free-volume model is developed that quantitatively accounts for the particle free energy in the hydrogel. Although the free volume accessible to a diffusor inside a hydrogel has been previously shown to determine diffusion properties in biological systems, such as crowded cellular membranes (52), our modified free-volume model additionally includes the elasticity of linkers and of the diffusing molecules and thereby quantitatively accounts for the free energies we extracted from the experimental data of dextran diffusing in PEG-based hydrogels. This demonstrates that elastic deformations of both the diffusor and the hydrogel network are important, in line with previous computational (53, 54, 55) and experimental studies (56). Our model furthermore unveils significant topological disorder of the hydrogel pores and suggests that the dextran molecules preferentially partition into exceptionally large pores, which are locally even more enlarged because of PEG strand elasticity.

Diffusional barriers in biological systems often show a layered structure, as previously demonstrated for skin (29, 30, 31) and also known to be true for mucous membranes, which are found, for instance, in the gastrointestinal tract, schematically indicated in Fig. 7 *B*. For a layered system, Eq. 14 shows that the individual piecewise constant permeability coefficients *P*_*i*_ add up inversely as

(16) $$\frac{1}{P_{\text{tot}}}=\sum_{i}\frac{1}{P_{i}}=\sum_{i}\frac{e^{\beta \text{Δ}F_{i}}}{D_{i}}L_{i}=\sum_{i}\frac{L_{i}}{D_{i}K_{i}},$$

where the sum goes over all layers, represented by their respective diffusion constants *D*_*i*_, free-energy values *ΔF*_*i*_ or partition coefficients *K*_*i*_, and thicknesses *L*_*i*_. Here, *P*_tot_ denotes the total permeability, which is dominated by the smallest permeability in the inverse sum.

Fig. 7 *B* schematically illustrates permeation through a layered system which represents the mammalian stomach (57). The outermost layer of mucus is only loosely bound and characterized by the permeability *P*_1_; it is followed by a layer of more tightly bound mucus, characterized by *P*_2_, and adheres onto the first layer of epithelial cells, characterized by *P*_3_. The total thickness of this diffusional barrier is about a millimeter, with the two mucus layers spanning a few hundred micrometers only (58). Measurements in rat gastrointestinal mucosa suggest typical values of *L*_1_ = 109 *μ*m, *L*_2_ = 80 *μ*m, and *L*_3_ ≈ *L*_2_ (59), which are close to the range of gel thicknesses studied in this work.

The total permeability is determined by the free energies and the mobilities inside all layers. Nutrients, for instance, can easily penetrate through the epithelia of the gastrointestinal tract, displaying large permeabilities in the different layers. Pathogens, on the other hand, are in healthy environments kept from reaching the epithelium because of low permeability in the tightly bound mucus layer (*P*_2_ ≪ *P*_1_) (57). From Eq. 16, it is apparent that the lowest permeability in such a layered system dominates the total permeability, leading to an effective barrier function that for different particles can be caused by different parts of the layered barrier structure.

The method introduced in this work determines free-energy and diffusivity profiles from experimental data and thereby can be used to predict effective permeabilities of different kinds of molecules, particles, or even organisms into various layered systems, including systems that contain hydrogels and mucus. A multilayered structure, as shown in Fig. 7 *B*, can be produced by cultivating mucous-producing cells in vitro and can be studied using the framework introduced in this work. This would allow the detailed analysis of permeabilities of different diffusors through an in vitro representation of an actual biological barrier. We believe that the technical advances described in this work will help to shed light on the underlying mechanisms of the function of general biological barriers including mucous membranes.

## Author Contributions

A.H., S.B., and R.H. designed and performed the CLSM experiments for the determination of the dextran concentration profiles. A.P. and M.G. designed and performed the FCS experiments. A.W.-K. and R.R.N. designed the models, analyzed the experimental data, and wrote the manuscript.

## Supporting Citations

References (60, 61, 62, 63, 64, 65, 66, 67, 68) appear in the Supporting Material.

## Supporting Material

Document S1. Supporting Materials and Methods, Figs. S1–S6, and Tables S1 and S2

Document S2. Article plus Supporting Material
