# Supplementary material for: Particle Diffusivity and Free-Energy Profiles in Hydrogels from Time-Resolved Penetration Data
Source: Biophys J. 2021 Jan 7;120(3):463–75. doi: 10.1016/j.bpj.2020.12.020 (PMC7896003; doi:10.1016/j.bpj.2020.12.020)
Supplement: Document S1. Supporting Materials and Methods, Figs. S1–S6, and Tables S1 and S2 [file mmc1.pdf]

Biophysical Journal, Volume *120*

Supplemental Information

Particle Diffusivity and Free-Energy Profiles in Hydrogels from Time-Resolved Penetration Data

Amanuel Wolde-Kidan, Anna Herrmann, Albert Prause, Michael Gradzielski, Rainer Haag, Stephan Block, and Roland R. Netz

S1 Molecular Mass Distributions of PEG linkers and Dextran Molecules

The dextran molecular mass distribution is characterized using gel permeation chromatography (GPC), the results of which are presented in Table S1. GPC measurements are performed on an *Agilent* device (1100er series) with a PSS Suprema column (pre-column, 1x with poresize of 30 Å, 2x with poresize of 1000 Å, all of them with a particle size of 10 μm), with pullulan as calibration standard, and ethylene glycol as internal standard. As solvent, H_2O with 0.1 M $NaNO_3$ is used. For the characterization of the PEG linker mass distribution matrix assisted laser desorption ionization (MALDI) on a *Bruker Ultraflex* II is performed. The obtained values are shown in Table S2. From the number average molecular mass M_n and the weight average molecular mass M_w the polydispersity index PDI is determined as

$$PDI = \frac{M_\mathrm{w}}{M_\mathrm{n}}. \tag{S1}$$

The dextran molecules show a considerable dispersion regarding the molecular mass, while the PEG linker mass distribution is rather uniform.

Table S1: Results obtained from GPC measurements of the different dextran molecules.

M_dex [kDa]	M_w [kDa]	M_n [kDa]	PDI
4	3.55	2.32	1.53
10	9.55	5.55	1.72
20	16.5	9.42	1.75
40	35.7	19.5	1.84
70	61.9	50.1	1.24

Table S2: Molecular weights and polydispersity of the PEG linkers as measured in MALDI experiments.

M_PEG [kDa]	M_w [kDa]	M_n [kDa]	PDI
6	6.29	5.90	1.07
10	11.3	11.3	1.00

S2 Hydrogel Volume Reconstruction

The hydrogel volume is determined using confocal laser scanning microscopy as follows. The hydrogels are first equilibrated using PBS buffer, followed by injection of the $M_{\text{dex}} = 70$ kDa FITC-labeled dextran and recording of 3D dextran concentration profiles using confocal microscopy (covering an imaged volume of 2,304 x 2,304 x 0.5 mm^3). The permeation measurements (shown in the main text) reveal that this dextran is too large to penetrate the hydrogel, so that the hydrogel can be identified in these 3D concentration profiles based on an exclusion of FITC-labeled dextran (i.e., absence of FITC fluorescence; see Figure S1). In order to quantify the hydrogel volume, the (2D) hyperplane at which the FITC intensity has dropped to 50% of its bulk value is determined using home-written scripts in *Matlab* (*MathWorks*, Natick, MA). This hyperplane indicates the positions, at which the point spread function of the confocal microscope is equally filled by the FITC-dextran bulk solution and either the hydrogel or the supporting substrate, and therefore allows for extraction of the exact locations of the substrate and hydrogel interface within the sample. Furthermore, as the substrate is a flat glass slide, the hydrogel-substrate interface is extracted from this data by first fitting a plane to the regions corresponding to the substrate-bulk interface (i.e., in regions far away from the hydrogel spot) and by interpolating the position of this plane underneath the hydrogel. This procedure allows for extraction of the entire hydrogel boundary, allowing determination of the hydrogel volume by numerical integration. This yields volumes of $V_{\text{tot}}^{\text{hPG-G6}} = 0.42 \pm 0.03$ μL and $V_{\text{tot}}^{\text{hPG-G10}} = 0.31 \pm 0.04$ μL for the two hydrogels.

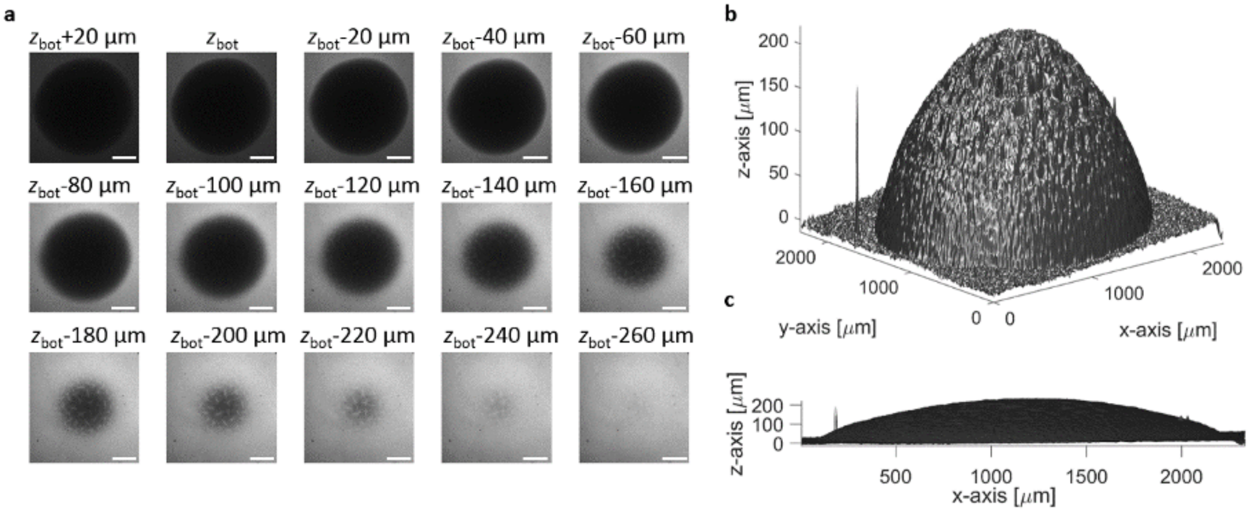

Supplementary Figure S1: In these experiments, the hydrogels have been equilibrated using PBS buffer (as described in Methods Section of the main text) followed by application of a $M_{\mathrm{dex}} = 70$ kDa dextran solution. The dextran is too large to penetrate into the hydrogel as evidenced by using confocal fluorescence microscopy to record multiple sample sections (at different z-heights as indicated in (a)). In these images, the hydrogel appears as a black circle, the radius of which decreases with increasing distance to the glass interface (located at the z-height z_{bot}). The bright areas correspond to FITC-dextran solution in the bulk. These images can be used to extract the position of the glass interface and hydrogel in space. A 3D representation and a side view of this hyperplane are given in (b) and (c), respectively, showing that the hydrogels possess a semi-elliptical shape with radii being on the order of 1050 μm (major axis) and 150 – 210 μm (minor axis). This figure shows a representative measurement for the *hPG-G6* gel. The scale bars in (a) correspond to 500 μm.

S3 Fitting Procedure for FCS Measurements

An accurate description of the dextran autocorrelation functions (ACFs) G(τ) required to use at least 2 components, which originate from the fluorescence emission of the FITC-labeled dextrans and, in addition, from residuals of free FITC molecules (i.e., not being conjugated to dextran). The FCS ACF G(τ) was therefore described using the equation

$$G(\tau) = T(\tau) \cdot \left(G_{\mathrm{F}}(\tau) + G_{\mathrm{DF}}(\tau)\right), \tag{S2}$$

in which $G_{\mathrm{F}}(\tau)$ and $G_{\mathrm{DF}}(\tau)$ give the contributions from free FITC molecules and FITC-labeled dextrans, respectively, while the term $T(\tau)$ accounts for triplet state dynamics according to

$$T(\tau) = \frac{\left(1 - \Phi_{\mathrm{T}} + \Phi_{\mathrm{T}} e^{-\frac{\tau}{\tau_{\mathrm{T}}}}\right)}{1 - \Phi_{\mathrm{T}}}, \tag{S3}$$

with Φ_{T} denoting the fraction of molecules in the triplet state and τ_{T} the corresponding decay time of triplet states.[1]

The contribution of the free FITC molecules was modeled using the theoretically derived ACF for free diffusion in 3D

$$G_{\mathrm{F}}(\tau) = \rho_{\mathrm{F}} \left(1 + \frac{\tau}{\tau_{\mathrm{F}}}\right)^{-1} \left(1 + \frac{\tau}{\tau_{\mathrm{F}} \kappa^2}\right)^{-\frac{1}{2}}, \tag{S4}$$

in which ρ_{F} denotes the average number density of free FITC molecules in the confocal readout volume, τ the lag time of the ACF, κ the ratio of axial r_{z} to radial extension r_{xy} of the confocal readout volume, and τ_{F} the decay time.[2] The value of τ_{F} was determined from calibration measurements on FITC molecules diffusing in buffer and κ was fixed to 6,[3] so that only the density ρ_{F} was a free parameter when fitting the component $G_{\mathrm{F}}(\tau)$ to experimentally determined ACF.

Since the dextrans showed a log-normal size distribution, as observed in the GPC mea-

surements, the component of FITC-labeled dextrans of the ACF, $G_{\mathrm{DF}}(\tau)$, was modeled by the superposition

$$G_{\mathrm{DF}}(\tau) = \sum_{i} p_{\mathrm{i}} G_{\mathrm{DF,i}}(\tau), \tag{S5}$$

using the log-normally distributed weights

$$p_{\mathrm{i}} := \frac{1}{\tau_{\mathrm{DF,i}} \sigma_{\mathrm{DF}} \sqrt{2\pi}} e^{-\frac{\left(\ln(\tau_{\mathrm{DF,i}}) - \mu_{\mathrm{DF}}\right)^2}{2\sigma_{\mathrm{DF}}^2}}, \tag{S6}$$

and the corresponding ACF contributions

$$G_{\mathrm{DF,i}}(\tau) := \rho_{\mathrm{DF}} \left(1 + \frac{\tau}{\tau_{\mathrm{DF,i}}}\right)^{-1} \left(1 + \frac{\tau}{\tau_{\mathrm{DF,i}} \kappa^2}\right)^{-\frac{1}{2}}. \tag{S7}$$

The parameter σ_{DF}, which determines the broadness of the log-normal distribution, was determined by matching the FCS-related polydispersity index (PDI), defined by

$$PDI_{\mathrm{FCS}} = \frac{\sum_i p_i M_i^2}{\left(\sum_i p_i M_i\right)^2}, \tag{S8}$$

using

$$M_i \propto R_i^3 \tag{S9a}$$

$$R_i = \frac{k_{\mathrm{B}} T}{6\pi\eta D_i} \tag{S9b}$$

$$D_i = \frac{r_{\mathrm{xy}}^2}{4\tau_{\mathrm{DF,i}}}, \tag{S9c}$$

where the PDI of the dextran mass distribution was determined using GPC. The parameters ρ_{DF} and $\tau_{\mathrm{DF}} = e^{\mu_{\mathrm{DF}}}$ denote the average number density of FITC-labeled dextran molecules in the confocal readout volume and their average decay time, respectively, and were determined when fitting the component $G_{\mathrm{DF}}(\tau)$ to the experimentally determined ACF.

Fitting the 2-component model therefore yields information about the number densities of free FITC molecules and FITC-labeled dextran molecules in the confocal readout volume (ρ_{F} and ρ_{DF}, respectively) and their decay times τ_{F} and τ_{DF}, which can be translated into diffusion coefficients using

$$D_{\mathrm{F}} = \frac{r_{\mathrm{xy}}^2}{4\tau_{\mathrm{F}}} \tag{S10a}$$

$$D_{\mathrm{DF}} = \frac{r_{\mathrm{xy}}^2}{4\tau_{\mathrm{DF}}}, \tag{S10b}$$

and into hydrodynamic radii using the Stokes-Einstein relation.[2]

S4 Measured Fluorescence Intensity Data

The experimentally measured fluorescence intensity data displays a continuous drift in the signal in all recorded measurements, which is likely due to an automatic re-adjustment of the laser intensity in the used setup. An example of the observed drift in the raw unscaled signal is shown in Figure S2A, recorded for $M_{\mathrm{dex}} = 70$ kDa dextran molecules at the *hPG-G10* interface. Even though almost no penetration of the large dextran molecules into the hydrogel is observed, the fluorescence intensity in the probed part of the bulk solution changes significantly over time. In order to obtain physical values for the dextran concentration, the measured profiles are being re-scaled during the fitting procedure. The obtained re-scaling factors for every measured concentration profile $\vec{f}$ decline over time, thus overcoming the constant increase of signal intensity due to the drift (see Figure S3A). Additionally, smaller changes in the fluorescence intensity are apparent. Since robust results are obtained by employing this re-scaling routine, this suggests that the entire information about the diffusion process is present in the relative shape of the concentration profiles.

The obtained re-scaling factor can additionally be used, to estimate the experimental bulk concentration c_{bulk} far away from the hydrogel interface, based on the experimentally measured profiles alone, without using the numerically computed dextran distributions. The total amount of dextran in the system C_{tot} is computed from the first concentration profile as $C_{\mathrm{tot}} = \int_{-\infty}^{\infty} c(z, t = 0)\, dz$, where $c(z, t = 0)$ was approximated by c_i^{init} according to eq. (4) in the main text. An average experimental concentration in the bulk region $\overline{c}_{\mathrm{bulk}}(t_{\mathrm{j}})$ can then be estimated from the fitted re-scaling factors as

$$\overline{c}_{\mathrm{bulk}}(t_{\mathrm{j}}) = \frac{C_{\mathrm{tot}} - \sum_{i=1}^{M} f_{\mathrm{j}} \cdot c_i^{\exp}(t_j) \cdot \Delta z_i}{z_{\mathrm{top}}}. \tag{S11}$$

Values for $\overline{c}_{\mathrm{bulk}}(t_{\mathrm{j}})$ are shown in Figure S3B and are virtually constant for the exemplary measurement of $M_{\mathrm{dex}} = 70$ kDa dextrans, as is expected due to the absence of penetration into the hydrogel.

The re-scaling procedure is additionally validated by a comparison of the concentration profiles obtained for the M_{dex} = 40 kDa dextran molecules, diffusing into the *hPG-G10* hydrogel, which is presented in Fig. S2B. The concentration profiles were measured in two different experiments, with a period of one month in between. The observed good agreement between the data sets, with significant deviations being only present in the transition region between the hydrogel and the bulk solution, corroborates the re-scaling method used to obtain concentration profiles from the fluorescence intensity data, as well as the experimental setup.

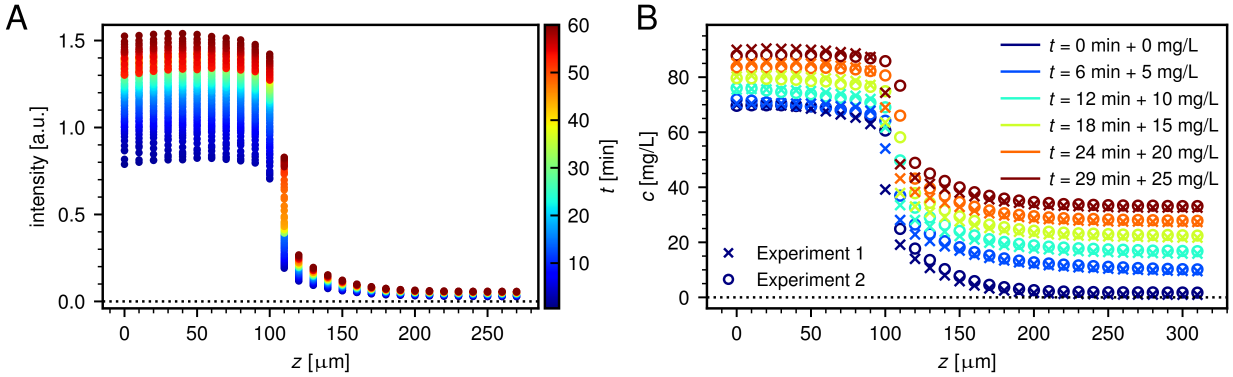

Supplementary Figure S2: A: Raw fluorescence intensity data from experiments of M_{dex} = 70 kDa dextrans in combination with the *hPG-G10* hydrogel. A significant change in the signal over time is observed in the probed part of the bulk solution, even though almost no penetration of the dextrans into the hydrogel is apparent. This drift in the experimentally measured signal is overcome by the numerically determined re-scaling factors. B: Rescaled concentration profiles measured one month apart in two different experiments for the M_{dex} = 40 kDa dextran molecules, penetrating into the *hPG-G10* gel. Good reproducibility is observed in the data. Note that profiles at different timepoints are shifted for an easier comparison.

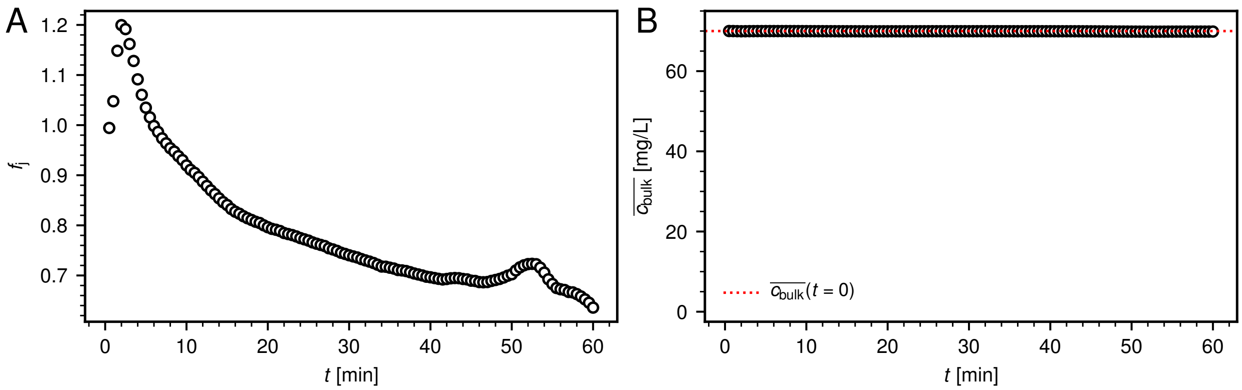

Supplementary Figure S3: A: Set of re-scaling factors $\vec{f}$ for every measured concentration profile obtained from numerical analysis of experimental data from $M_{\mathrm{dex}} = 70$ kDa dextrans in contact with the *hPG-G10* hydrogel. Decreasing re-scaling factors counteract the drift observed in the raw experimental data. Additionally, peaks in the re-scaling factor distribution are observed, counteracting shorter fluctuations in the fluorescence intensity. B: Average bulk concentration $\overline{c_{\mathrm{bulk}}}$ computed according to eq. (S11) for the same measurements. The bulk concentration remains constant in this measurement, since almost no dextran penetrates into the hydrogel.

S5 Analytical Solution for Two-Segment System

Simplifying the hydrogel-water setup as a two-box system with piece-wise constant values of the free-energy and diffusion constant in the two regions allows for an analytical solution of the diffusion problem. The modeled system with the corresponding boundary conditions is sketched in Figure S4A.

We solve the following diffusion equation in each of the two segments

$$\frac{\partial}{\partial t}c(z,t) = D(z)\frac{\partial^2}{\partial z^2}c(z,t), \tag{S12}$$

where the diffusion constant $D(z)$ has a different value in each of the two regions

$$D(z) = \begin{cases} D_0, & \text{if } 0 \leq z \leq z_{\text{int}} \\ D_1, & \text{if } z_{\text{int}} < z \leq z_{\text{bot}} \end{cases} \tag{S13}$$

as does the free-energy $F(z)$, which we set to zero in the left segment as reference

$$F(z) = \begin{cases} F_0 = 0, & \text{if } 0 \leq z \leq z_{\text{int}} \\ F_1, & \text{if } z_{\text{int}} < z \leq z_{\text{bot}}. \end{cases} \tag{S14}$$

At the interface z_{int}, the flux needs to be continuous due to mass conservation, while the jump in the free-energy leads to a jump in the concentration profile $c(z = z_{\text{int}}, t)$. This defines the boundary conditions at z_{int} as

$$\lim_{z \nearrow z_{\text{int}}} D_0 \frac{\partial}{\partial z}c(z,t) = \lim_{z \searrow z_{\text{int}}} D_1 \frac{\partial}{\partial z}c(z,t) \tag{S15a}$$

$$\lim_{z \nearrow z_{\text{int}}} c(z,t)e^{-\beta F_1} = \lim_{z \searrow z_{\text{int}}} c(z,t), \tag{S15b}$$

Since we are modeling a closed system, the edges at $z = 0$ and $z = z_{\text{bot}}$ are reflecting

boundaries with

$$\frac{\partial}{\partial z}c(z=0,t)=0 \tag{S16a}$$

$$\frac{\partial}{\partial z}c(z=z_{\mathrm{bot}},t)=0. \tag{S16b}$$

Initially, the diffusors are only present in the left segment, modeling the bulk solution. This defines our initial condition as

$$c(z,t=0)=\begin{cases} c_0, & \text{if } 0\le z\le z_{\mathrm{int}} \\ 0, & \text{if } z_{\mathrm{int}}<z\le z_{\mathrm{bot}}. \end{cases} \tag{S17}$$

We now solve eq. (S12) by means of Laplace transformation. To this end, we use the single sided Laplace transform in time, defined as $\hat{f}(s):=\int_0^\infty f(t)e^{-st}dt$, where s is the complex variable in Laplace space $s=\sigma+i\omega$. This converts the partial differential equation (S12) into an ordinary differential equation of second order

$$\left[s-D(z)\frac{\partial^2}{\partial z^2}\right]\hat{c}(z,s)=c(z,t=0). \tag{S18}$$

The general solution of eq. (S18) for the two regions reads

$$\hat{c}(z,s)=\begin{cases} a_1e^{\lambda_0 z}+a_2e^{-\lambda_0 z}+\hat{c}_{\mathrm{p}}, & \text{if } 0\le z\le z_{\mathrm{int}} \\ a_3e^{\lambda_1 z}+a_4e^{-\lambda_1 z}, & \text{if } z_{\mathrm{int}}<z\le z_{\mathrm{bot}} \end{cases} \tag{S19}$$

where we define $\lambda_{\mathrm{i}}:=\sqrt{\frac{s}{D_{\mathrm{i}}}}, i=0,1$ and $\hat{c}_{\mathrm{p}}:=\frac{c_0}{s}$. The coefficients a_i of eq. (S19) are determined by solving the system of linear equations obtained by Laplace transforming the boundary conditions of eq. (S15) and eq. (S16) and substituting the general solution (eq. (S19)). After some algebra, the solution to the posed problem is obtained as

$$\hat{c}(z,s) = \begin{cases} \hat{c}_\mathrm{p} \frac{K \cdot \tanh(\lambda_1(z_\mathrm{bot}-z_\mathrm{int}))[\cosh(\lambda_0 z_\mathrm{int})-\cosh(\lambda_0 z)]+\sinh(\lambda_0 z_\mathrm{int})\sqrt{\delta}}{K \cdot \tanh(\lambda_1(z_\mathrm{bot}-z_\mathrm{int}))\cosh(\lambda_0 z_\mathrm{int})+\sinh(\lambda_0 z_\mathrm{int})\sqrt{\delta}}, & \text{if } 0 \leq z \leq z_\mathrm{int} \\ \hat{c}_\mathrm{p} \frac{K \cdot \cosh(\lambda_1(z_\mathrm{bot}-z))\tanh(\lambda_0 z_\mathrm{int})\sqrt{\delta}}{K \cdot \sinh(\lambda_1(z_\mathrm{bot}-z_\mathrm{int}))+\tanh(\lambda_0 z_\mathrm{int})\cosh(\lambda_1(z_\mathrm{bot}-z_\mathrm{int}))\sqrt{\delta}}, & \text{if } z_\mathrm{int} < z \leq z_\mathrm{bot} \end{cases} \tag{S20}$$

where $\delta := \frac{D_0}{D_1}$ and $K := e^{-\beta F_1}$.

The solution in Laplace space (eq. (S20)) is then transformed into real space by use of the Mellin integral

$$\begin{aligned} c(z,t) &= \frac{1}{2\pi i} \int_{s=\sigma-i\infty}^{s=\sigma+i\infty} \hat{c}(z,s) e^{st}\, ds \\ &= \frac{e^{\sigma t}}{2\pi} \int_{-\infty}^{+\infty} \hat{c}(z, \sigma + i\omega) e^{i\omega t}\, dw, \end{aligned} \tag{S21}$$

where the last integral was solved numerically through the inverse discrete Fourier transform.

Figure S4B shows a comparison of the analytical solution and the numerical model for an exemplary parameter set of $z_\mathrm{int} = 100$ μm, $z_\mathrm{bot} = 300$ μm, $c_0 = 1$ mg/L, $D_0 = 50$ $\mu\mathrm{m}^2/s$, $D_1 = 100$ $\mu\mathrm{m}^2/s$ and $F_1 = 0.5$ $k_\mathrm{B}T$, mimicking a slight immobilization and repulsion in the right segment, as observed for the smaller dextrans in the experiments (see main text). The stationary state is reached faster in the approximate system compared to the actual measurements in the main text, due to the much smaller z-dimension. Perfect agreement between the numerical model and the analytical solution is obtained.

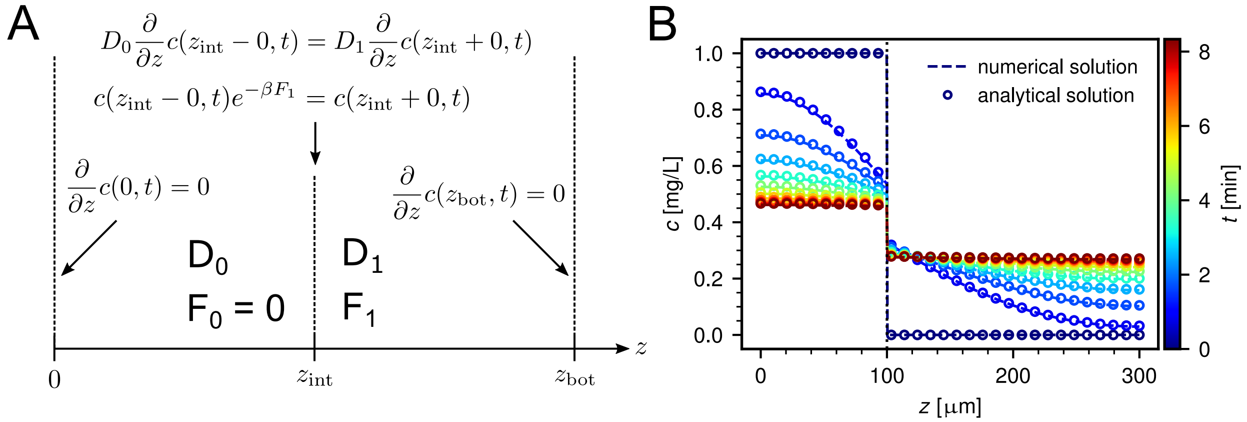

Supplementary Figure S4: Approximation of the dextran hydrogel setup as a two-segment system, which can be solved analytically. A: Two-segment system with different diffusion constants and a jump in the free-energy at the interface z_{int}. The used boundary conditions are also indicated. B: Comparison of the numerical model and the analytical solution of the system explained in A, with values for the parameters of $z_{\mathrm{int}} = 100$ μm, $z_{\mathrm{bot}} = 300$ μm, $c_0 = 1$, $D_0 = 50$ $\mu\mathrm{m}^2/s$, $D_1 = 100$ $\mu\mathrm{m}^2/s$ and $F_1 = 0.5$ $k_{\mathrm{B}}T$.

S6 Error Estimate for Numerical Analysis

In order to determine confidence intervals for the fitted parameters of D_{sol}, D_{gel} and ΔF_{gel}, the values are varied from the optimum until the agreement with the experimental data is 50% worse than for the optimal parameter values. Figure S5 shows an exemplary analysis of the fitted parameters influence on the error. All parameters are varied independently, meaning that the error is always computed while keeping all other parameters fixed at their optimal values. Also, the fitted values for d_{int} and z_{int} are not changed but kept at their optimum. It is apparent that increasing the fitted diffusion constants does not affect the agreement with the experimental data as strongly as a decrease (see Figure S5A and B). Changing the free-energy difference influences the numerical error σ more symmetrically, meaning increasing ΔF_{gel} has the same influence on the error as decreasing it.

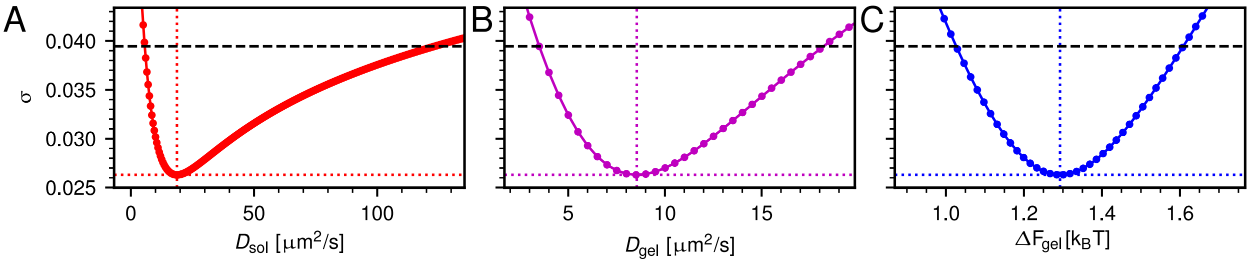

Supplementary Figure S5: Error estimation of the fitted values for D_{sol} (A), D_{gel} (B) and ΔF_{gel} (C) for measurements of $M_{\text{dex}} = 40$ kDa dextran molecules diffusing into the *hPG-G10* hydrogel. Fitted optimal values for the parameters are indicated by dotted lines, while a 50% change in σ is shown by the dashed black line. A larger value of the diffusion constants does not affect the agreement with the experimental data as strongly as a smaller value.

S7 Scaling of Diffusion Constant with Dextran Size

According to the Stokes-Einstein relation, the diffusion constant of dextran molecules in the bulk solution D_{sol} is expected to scale with the dextran radius r_0 as

$$D_{\mathrm{sol}} \propto r_0^{-1}. \tag{S22}$$

Assuming the dextran polymer behaves like a freely jointed chain, its radius is related to the number of monomers N_{dex} as[4,5]

$$r_0 = b_0^{\mathrm{dex}} \sqrt{N_{\mathrm{dex}}}, \tag{S23}$$

where b_0^{dex} is the dextran monomer length and N_{dex} can be estimated from the total molecular mass of a dextran molecule M_{dex}, when the monomer mass $M_{\mathrm{dex}}^{\mathrm{mono}}$ is known

$$N_{\mathrm{dex}} = \frac{M_{\mathrm{dex}}}{M_{\mathrm{dex}}^{\mathrm{mono}}}. \tag{S24}$$

This leads to the following equality

$$D_{\mathrm{sol}} = \frac{k_{\mathrm{B}} T}{6\pi\eta_{\mathrm{w}} \frac{b_0^{\mathrm{dex}}}{\sqrt{M_{\mathrm{dex}}^{\mathrm{mono}}}} \sqrt{M_{\mathrm{dex}}}}, \tag{S25}$$

which gives rise to a scaling of $D_{\mathrm{sol}} \propto M_{\mathrm{dex}}^{-1/2}$.

S8 Expression for the Elastic Deformation Free Energy

The free-energy cost for stretching a polymer chain from an initial equilibrium mean square end-to-end distance $\langle \vec{R}_0^2 \rangle$ to a larger end-to-end distance $\langle \vec{R}^2 \rangle$ can be written as[4,5]

$$\Delta F_{\text{stretch}} = \frac{3}{2} k_{\text{B}} T \frac{\langle \vec{R}^2 \rangle - \langle \vec{R}_0^2 \rangle}{\langle \vec{R}_0^2 \rangle}, \tag{S26}$$

where $k_{\text{B}}T$ denotes the thermal energy. In the case of the PEG linkers we define the z-component of the squared end-to-end distance as $l^2 := \langle R_z^2 \rangle$, so that $l_0^2 := \langle R_{0,z}^2 \rangle$. The PEG polymer chain is only stretched in the z-direction, thus eq. (S26) reduces to

$$\Delta F_{\text{stretch}} = \frac{3}{2} k_{\text{B}} T \frac{\langle R_x^2 \rangle + \langle R_y^2 \rangle + l^2 - \langle R_{0,x}^2 \rangle - \langle R_{0,y}^2 \rangle - l_0^2}{\langle \vec{R}_0^2 \rangle} = \frac{3}{2} k_{\text{B}} T \frac{l^2 - l_0^2}{\langle \vec{R}_0^2 \rangle}, \tag{S27}$$

since $\langle R_x^2 \rangle = \langle R_{0,x}^2 \rangle$ and $\langle R_y^2 \rangle = \langle R_{0,y}^2 \rangle$. As the PEG polymer chain performs a random walk in all three spatial dimensions, all components of the mean squared end-to-end distance contribute equally and so

$$\frac{\langle \vec{R}_0^2 \rangle}{3} = \langle R_{0,x}^2 \rangle = \langle R_{0,y}^2 \rangle = \langle R_{0,z}^2 \rangle = l_0^2. \tag{S28}$$

Together with eq. (S27), eq. (S28) leads to the stretching free-energy for a single PEG linker polymer chain

$$\Delta F_{\text{stretch}} = \frac{1}{2} k_{\text{B}} T \left[\frac{l^2}{l_0^2} - 1 \right]. \tag{S29}$$

For the compression of a polymer chain from an initially larger mean square end-to-end distance $\langle \vec{R}_0^2 \rangle$ to a smaller one $\langle \vec{R}^2 \rangle$ we write[4,5]

$$\Delta F_{\text{compress}} = \frac{3}{2} k_{\text{B}} T \frac{\langle \vec{R}_0^2 \rangle - \langle \vec{R}^2 \rangle}{\langle \vec{R}^2 \rangle}. \tag{S30}$$

In the same way as above, we only allow compression along the z-axis, which leads to

$$\Delta F_{\text{compress}} = \frac{3}{2}k_{\text{B}}T\frac{\langle R_{0,x}^2\rangle + \langle R_{0,y}^2\rangle + l_0^2 - \langle R_x^2\rangle - \langle R_y^2\rangle - l^2}{\langle R_x^2\rangle + \langle R_y^2\rangle + l^2} = \frac{3}{2}k_{\text{B}}T\frac{l_0^2 - l^2}{\langle R_{0,x}^2\rangle + \langle R_{0,y}^2\rangle + l^2}. \tag{S31}$$

Using eq. (S28) to substitute the x- and y-components of the equilibrium end-to-end distance gives the expression for the compression free-energy of a single PEG linker

$$\Delta F_{\text{compress}} = \frac{1}{2}k_{\text{B}}T\frac{3l_0^2 - 3l^2}{2l_0^2 + l^2}. \tag{S32}$$

The total elastic deformation free-energy per PEG linker is the sum of eq. (S29) and eq. (S32)

$$\Delta F_{\text{PEG}} = \frac{1}{2}k_{\text{B}}T\left(\left[\frac{l}{l_0}\right]^2 + \frac{l_0^2 - 4l^2}{2l_0^2 + l^2}\right). \tag{S33}$$

For the twelve PEG linkers of the hydrogel unit cell this leads to eq. (10) of the main text.

S9 Estimating PEG-Monomer Hydration Number

Based on eq. (7) and eq. (12) from the main text, we obtain the following relation between the partition coefficient K_{gel} and the volume accessible to the dextran diffusors V_{free}

$$K_{\text{gel}} = \frac{V_{\text{free}}}{V_{\text{unit}}} e^{-\beta(\Delta F_{\text{dex}} + \Delta F_{\text{PEG}})}. \tag{S34}$$

The volume inaccessible to the dextran molecules V_{ex} (see eq. (8) of the main text) is composed of a part occupied by the gel or dextran directly and a part due to tightly bound hydration water, so that

$$V_{\text{ex}} = V_{\text{unit}} - V_{\text{free}} = V_{\text{gel}} + V_{\text{hyd}} + V_{\text{dex}}, \tag{S35}$$

where $V_{\text{gel}} = V_{\text{PEG}} + V_{\text{hPG}}$ denotes the excluded volume due to both gel components, $V_{\text{dex}} = 3\pi r^2 l - \frac{32}{3}\pi r^3$ (with r and l as explained in Figure 6A in the main text) denotes the excluded volume due to dextran, $V_{\text{unit}} = l^3$ is the unit cell volume and V_{hyd} is the volume occupied by hydration water. Since r denotes the hydrodynamic radius of the spherical dextran, we assume V_{hyd} to be the volume of hydration water only binding to the gel components.

The mass fraction Φ_{gel} of the gel components inside the hydrogel is defined as the ratio of the mass of the gel components m_{gel} to the total mass m_{tot}, but since the mass density of the gel components is comparable to that of water, it also represents the fraction of inaccessible volume due to the gel components

$$\Phi_{\text{gel}} := \frac{m_{\text{gel}}}{m_{\text{tot}}} \approx \frac{V_{\text{gel}}}{V_{\text{unit}}}. \tag{S36}$$

In the same fashion we can also estimate the fraction of inaccessible volume due to only the PEG linkers as

$$\Phi_{\text{PEG}} = \frac{m_{\text{PEG}}}{m_{\text{tot}}} = \frac{n_{\text{PEG}} M_{\text{PEG}}}{m_{\text{tot}}} \frac{V_{\text{app}}}{V_{\text{gel}}^{\text{sol}}} \approx \frac{V_{\text{PEG}}}{V_{\text{unit}}}, \tag{S37}$$

where we use the values for the number n_{PEG} and the molar mass M_{PEG} of the PEG linkers given in Table 1 of the Methods Section. The factor of $V_{\mathrm{app}}/V_{\mathrm{gel}}^{\mathrm{sol}}$ accounts for the fact that only $V_{\mathrm{app}} = 1\ \mu$L of the total volume of prepared gel solution $V_{\mathrm{gel}}^{\mathrm{sol}}$ are actually placed on the gel spot for the experiments. The total mass of the hydrogel m_{tot} is estimated from the measured hydrogel volumes by using the water mass density (cf. also Section S2). Combining eq. (S34), eq. (S35), and eq. (S36) from above gives the following expression for volume fraction occupied by hydration water

$$\frac{V_{\mathrm{hyd}}}{V_{\mathrm{unit}}} = 1 - \Phi_{\mathrm{gel}} - K_{\mathrm{gel}} e^{\beta(\Delta F_{\mathrm{dex}} + \Delta F_{\mathrm{PEG}})} - 3\pi \left(\frac{r}{l}\right)^2 + \frac{32}{3}\left(\frac{r}{l}\right)^3. \tag{S38}$$

The hydration water of eq. (S38) in principle binds to the entire hydrogel, meaning the hPG hubs and the PEG linkers. For a rough estimate, we neglect the presence of the hPG hubs and assume V_{hyd} is the volume of water molecules hydrating only the PEG linkers. We can compute the fraction of hydration water per unit PEG volume from eq. (S37) and eq. (S38) as

$$\frac{V_{\mathrm{hyd}}}{V_{\mathrm{PEG}}} = \frac{n_{\mathrm{hyd}} v_{\mathrm{w}}}{n_{\mathrm{PEG}} v_{\mathrm{PEG}}} = \frac{1 - \Phi_{\mathrm{gel}} - K_{\mathrm{gel}} e^{\beta(\Delta F_{\mathrm{dex}} + \Delta F_{\mathrm{PEG}})} - 3\pi \left(\frac{r}{l}\right)^2 + \frac{32}{3}\left(\frac{r}{l}\right)^3}{\Phi_{\mathrm{PEG}}}, \tag{S39}$$

where n_{hyd} is the number of hydration water molecules, v_{w} is their partial volume, n_{PEG} is the number of PEG linkers and v_{PEG} is the PEG linker partial volume. The ratio between the partial volumes of water and the PEG linkers is approximated by the ratio of their molar masses as

$$\frac{v_{\mathrm{w}}}{v_{\mathrm{PEG}}} \approx \frac{M_{\mathrm{w}}}{M_{\mathrm{PEG}}}, \tag{S40}$$

with the water molar mass $M_{\mathrm{w}} = 18$ g/mol and the molar mass of the respective PEG linker M_{PEG} (see Methods Section). From eq. (S39), we can now compute the number of hydration waters per PEG linker molecule as

$$\frac{n_{\mathrm{hyd}}}{n_{\mathrm{PEG}}}=\frac{1-\Phi_{\mathrm{gel}}-K_{\mathrm{gel}}e^{\beta(\Delta F_{\mathrm{dex}}+\Delta F_{\mathrm{PEG}})}-3\pi\left(\frac{r}{l}\right)^2+\frac{32}{3}\left(\frac{r}{l}\right)^3}{\Phi_{\mathrm{PEG}}}\frac{M_{\mathrm{PEG}}}{M_{\mathrm{w}}}. \tag{S41}$$

In order to obtain the number of hydration waters per PEG-monomer we simply divide eq. (S41) by the respective number of PEG-monomers per linker N_{PEG}, which we obtain from the ratio of total linker mass M_{PEG} and PEG-monomer mass $M_{\mathrm{PEG}}^{\mathrm{mono}}=44$ g/mol, as $N_{\mathrm{PEG}}=M_{\mathrm{PEG}}/M_{\mathrm{PEG}}^{\mathrm{mono}}$. The number of hydration waters per PEG-monomer can thus be obtained as

$$\frac{n_{\mathrm{hyd}}}{n_{\mathrm{PEG}}^{\mathrm{mono}}}=\frac{1-\Phi_{\mathrm{gel}}-K_{\mathrm{gel}}e^{\beta(\Delta F_{\mathrm{dex}}+\Delta F_{\mathrm{PEG}})}-3\pi\left(\frac{r}{l}\right)^2+\frac{32}{3}\left(\frac{r}{l}\right)^3}{\Phi_{\mathrm{PEG}}}\frac{M_{\mathrm{PEG}}^{\mathrm{mono}}}{M_{\mathrm{w}}}. \tag{S42}$$

With the values of $K_{\mathrm{gel}}e^{\beta(\Delta F_{\mathrm{dex}}+\Delta F_{\mathrm{PEG}})}$, $V_{\mathrm{dex}}/V_{\mathrm{unit}}=3\pi\left(\frac{r}{l}\right)^2-\frac{32}{3}\left(\frac{r}{l}\right)^3$, Φ_{gel} and Φ_{PEG} for the two hydrogels from the main text, eq. (S42) allows us to estimate the number of hydration waters per PEG monomer for all measurements. Figure S6 shows the results of the calculation for each of the two hydrogels. Estimated values scatter around 8 water molecules per PEG monomer, with a slight dependence on the PEG linker length and dextran mass. Depending on the employed experimental method, values reported in the literature vary, ranging from 2 to 11 water molecules per PEG monomer.[6–10] Additionally, an increase of the hydration waters per monomer has been observed, as a function of the polymerization degree.[11] The values obtained from our estimates, all lie within the range of values reported in the literature, as indicated by the grey shaded area in Figure S6. This further corroborates our methodology and specifically the model for the free-energy of eq. (12), since the estimate of eq. (S42) is based on this model.

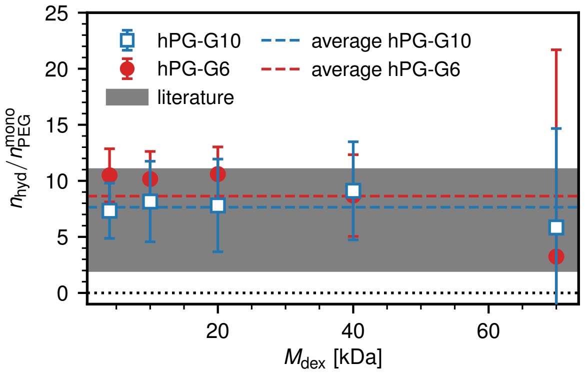

Supplementary Figure S6: Estimated number of water molecules per PEG monomer $n_{\mathrm{hyd}}/n_{\mathrm{PEG}}^{\mathrm{mono}}$ from the obtained values of K_{gel} for the two hydrogels based on eq. (S42). The estimated values scatter around $n_{\mathrm{hyd}}/n_{\mathrm{PEG}}^{\mathrm{mono}} = 8$ and agree with the range of values reported in the literature indicated as the grey shaded area, ranging from $n_{\mathrm{hyd}}/n_{\mathrm{PEG}}^{\mathrm{mono}} = 2$ to $n_{\mathrm{hyd}}/n_{\mathrm{PEG}}^{\mathrm{mono}} = 11$.[6–10]

S10 Permeability Coefficient

The definition of the permeability coefficient P is, as stated in the main text[12]

$$P(z_1, z_2) := \frac{J}{c(z_1) - c(z_2)}, \tag{S43}$$

with the stationary flux J and the equilibrium concentrations on both sides of the barrier $c(z_1)$ and $c(z_2)$. From the generalized diffusion equation (6) in the main text, one obtains the stationary flux for the case of $\partial c(z,t)/\partial t = 0$ as

$$J = D(z) e^{-\beta F(z)} \frac{\partial}{\partial z} \left(c(z,t) e^{\beta F(z)} \right), \tag{S44}$$

as a function of the diffusion constant $D(z)$ and the free-energy landscape $F(z)$ across the barrier. After rearranging eq. (S44) and integrating from one side of the barrier from z_1 to the other z_2, we obtain the following relation

$$J \int_{z_1}^{z_2} \frac{e^{\beta F(z)}}{D(z)} \, dz = c(z_1,t) e^{\beta F(z_1)} - c(z_2,t) e^{\beta F(z_2)}. \tag{S45}$$

We now assume that the free-energy value is the same on both sides of the barrier and additionally set it to zero as reference so that $F(z_1) = F(z_2) = 0$ and thus

$$J \int_{z_1}^{z_2} \frac{e^{\beta F(z)}}{D(z)} \, dz = c(z_1,t) - c(z_2,t), \tag{S46}$$

which, in combination with eq. (S43), gives eq. (14) used in the main text

$$\frac{1}{P} = \int_{z_1}^{z_2} \frac{e^{\beta F(z)}}{D(z)} \, dz. \tag{S47}$$

References

(1) Widengren, J.; Rigler, R.; Mets, Ü. Triplet-state monitoring by fluorescence correlation spectroscopy. *Journal of Fluorescence* **1994**, *4*, 255–258, DOI: 10.1007/BF01878460.

(2) Rigler, R.; Elson, E. S. *Fluorescence Correlation Spectroscopy: Theory and Applications*; Springer Series in Chemical Physics; Springer Berlin Heidelberg, 2012.

(3) Rüttinger, S.; Buschmann, V.; Krämer, B.; Erdmann, R.; MacDonald, R.; Koberling, F. Comparison and accuracy of methods to determine the confocal volume for quantitative fluorescence correlation spectroscopy. *Journal of Microscopy* **2008**, *232*, 343–352, DOI: 10.1111/j.1365-2818.2008.02105.x.

(4) Rubinstein, M.; Colby, R. H. *Polymer Physics*; OUP Oxford, 2003.

(5) Netz, R. R.; Andelman, D. Neutral and charged polymers at interfaces. *Physics Reports* **2003**, *380*, 1–95, DOI: 10.1016/S0370-1573(03)00118-2.

(6) Huang, L.; Nishinari, K. Interaction between poly(ethylene glycol) and water as studied by differential scanning calorimetry. *Journal of Polymer Science, Part B: Polymer Physics* **2001**, *39*, 496–506, DOI: 10.1002/1099-0488(20010301)39:5<496::AID-POLB1023>3.0.CO;2-H.

(7) Shikata, T.; Takahashi, R.; Sakamoto, A. Hydration of Poly(ethylene oxide)s in Aqueous Solution As Studied by Dielectric Relaxation Measurements. *The Journal of Physical Chemistry B* **2006**, *110*, 8941–8945, DOI: 10.1021/jp060356i.

(8) Kaatze, U.; Gottmann, O.; Podbielski, R.; Pottel, R.; Terveer, U. Dielectric relaxation in aqueous solutions of some oxygen-containing linear hydrocarbon polymers. *The Journal of Physical Chemistry* **1978**, *82*, 112–120, DOI: 10.1021/j100490a025.

(9) Bieze, T. W. N.; Barnes, A. C.; Huige, C. J. M.; Enderby, J. E.; Leyte, J. C. Distribution

of Water around Poly(ethylene oxide): A Neutron Diffraction Study. *The Journal of Physical Chemistry* **1994**, *98*, 6568–6576, DOI: 10.1021/j100077a024.

(10) Żwirbla, W.; Sikorska, A.; Linde, B. B. Ultrasonic investigations of water mixtures with polyethylene glycols 200, 400 and ethylene glycol. *Journal of Molecular Structure* **2005**, *743*, 49–52, DOI: 10.1016/j.molstruc.2005.02.019.

(11) Branca, C.; Magazù, S.; Maisano, G.; Migliardo, F.; Migliardo, P.; Romeo, G. Hydration Study of PEG/Water Mixtures by Quasi Elastic Light Scattering, Acoustic and Rheological Measurements. *The Journal of Physical Chemistry B* **2002**, *106*, 10272–10276, DOI: 10.1021/jp014345v.

(12) Diamond, J. M.; Katz, Y. Interpretation of nonelectrolyte partition coefficients between dimyristoyl lecithin and water. *The Journal of Membrane Biology* **1974**, *17*, 121–154, DOI: 10.1007/BF01870176.
