# Supplementary material for: Particle Diffusivity and Free-Energy Profiles in Hydrogels from Time-Resolved Penetration Data
Source: Biophys J. 2021 Jan 7;120(3):463–75. doi: 10.1016/j.bpj.2020.12.020 (PMC7896003; doi:10.1016/j.bpj.2020.12.020)
Supplement: Document S2. Article plus Supporting Material [file mmc2.pdf]

Biophysical Journal
Article

Biophysical Society

Particle Diffusivity and Free-Energy Profiles in Hydrogels from Time-Resolved Penetration Data

Amanuel Wolde-Kidan,[1] Anna Herrmann,[2] Albert Prause,[3] Michael Gradzielski,[3] Rainer Haag,[2] Stephan Block,[2] and Roland R. Netz[1,*]

[1]Fachbereich Physik and [2]Institut für Chemie und Biochemie, Freie Universität Berlin, Berlin, Germany; and [3]Institut für Chemie, Technische Universität Berlin, Berlin, Germany

ABSTRACT A combined experimental and theoretical method to simultaneously determine diffusivity and free-energy profiles of particles that penetrate into inhomogeneous hydrogel systems is presented. As the only input, arbitrarily normalized concentration profiles from fluorescence intensity data of labeled tracer particles for different penetration times are needed. The method is applied to dextran molecules of varying size that penetrate into hydrogels of polyethylene-glycol chains with different lengths that are covalently cross-linked by hyperbranched polyglycerol hubs. Extracted dextran bulk diffusivities agree well with fluorescence correlation spectroscopy data obtained separately. Empirical scaling laws for dextran diffusivities and free energies inside the hydrogel are identified as a function of the dextran mass. An elastic free-volume model that includes dextran as well as polyethylene-glycol linker flexibility quantitively describes the repulsive dextran-hydrogel interaction free energy, which is of steric origin, and furthermore suggests that the hydrogel mesh-size distribution is rather broad and particle penetration is dominated by large hydrogel pores. Particle penetration into hydrogels for steric particle-hydrogel interactions is thus suggested to be governed by an elastic size-filtering mechanism that involves the tail of the hydrogel pore-size distribution.

SIGNIFICANCE The barrier function of mucus and other biological hydrogels against particles and pathogens depends on their diffusivity and free-energy profiles. We introduce a method that allows for simultaneous extraction of these quantities from non-normalized concentration profiles measured in penetration experiments. We apply our method to fluorescently labeled dextran polymers diffusing into polyethylene-glycol-based hydrogels and explain the results by an elastic free-volume model. We conclude that the penetration is governed by the large pores of the broad pore-size distribution, which is most likely a general characteristic of hydrogels. Our method is generally applicable to various kinds of labeled particles, including bacteria and virions, and can be used to help unravel the mechanisms behind mucus barrier function.

INTRODUCTION

The penetration of particles into hydrogels is relevant for technological applications (1,2), drug delivery (3), and biological systems such as biofilms (4), the extracellular matrix (5), and mucus (6). Mucus, which is the most common biological hydrogel, lines the epithelial tissues of different organs, such as the respiratory, gastrointestinal, and urogenital tracts. Mucus is mainly composed of mucins, which are glycoproteins of varying length that absorb large amounts of water and thereby lend mucus its hydrogel nature, and additional components such as enzymes and ions (7). Mucins are relevant in the cell signaling context and presumably also play a role in the development of cancer (8). But primarily, mucus is a penetration barrier against pathogens, e.g., virions or bacteria, whereas it allows the permeation of many nonpathogens, e.g., nutrients, that are absorbed through the mucosa of the small intestine (9). Studies have suggested that based on the type of mucus, the combination of different mechanisms gives rise to the protective barrier function (10,11), in addition to the advective transport of pathogens through mucus shedding or clearance (12,13), which is not considered here. One typically distinguishes steric size-filtering mechanisms from interaction-filtering mechanisms (6,14); the latter

Submitted September 29, 2020, and accepted for publication December 23, 2020.

*Correspondence: rnetz@physik.fu-berlin.de

Amanuel Wolde-Kidan and Anna Herrmann contributed equally to this work.

Editor: Jennifer Curtis.

https://doi.org/10.1016/j.bpj.2020.12.020

© 2021 Biophysical Society.

This is an open access article under the CC BY license (http://creativecommons.org/licenses/by/4.0/).

presumably play a major role in the defense of organisms against pathogens because they allow for precise regulation of the passage of wanted and unwanted particles and molecules (15,16). Recent studies demonstrated that attractive electrostatic interactions reduce the particle diffusivity inside hydrogels substantially and much more than repulsive electrostatic interactions (17,18) and that salt concentration and the distribution of charges and pore sizes are important parameters that influence the permeation properties of charged hydrogels (19,20).

Particle penetration into mucus and biofilms has been studied by single-particle tracking techniques (21,22) as well as by methods in which a diffusor ensemble is observed (15,16,23,24). On short timescales, transient particle binding to the hydrogel (16–18) is important and leads to anomalous particle diffusion (25). On spatial length scales larger than the hydrogel mesh size and on timescales larger than typical binding escape times, particle diffusion is in a continuum description determined by the free-energy and diffusivity profiles across an inhomogeneous hydrogel system. In this framework, particle binding is effectively taken into account via a reduction of the diffusivity and a lowering of the free energy. If the free-energy and diffusivity profiles are known, particle penetration can be quantitatively predicted, provided the particle concentration is low and the particles do not modify the hydrogel properties in an irreversible manner. In this context, it should be noted that both profiles depend on the interactions between particle and hydrogel and therefore are different for each distinct hydrogel-particle pair. Because of method restrictions, experiments primarily focus on determining either the particle diffusivity inside the hydrogel (6,10,21) or on the partitioning between hydrogel and the bulk solution (26), from which the free energy inside the hydrogel (relative to the bulk solution) can be determined. However, for prediction of the penetration or permeation speed of particles into the hydrogel, both the diffusivity and the free energy in the hydrogel are needed.

In this work, we study synthetic hydrogels that consist of polyethylene-glycol (PEG) linkers of different molecular masses that are permanently cross-linked by hyperbranched polyglycerol (hPG) hubs (2). Such synthetic hydrogels can be regarded as simple models for mucus because they display size-dependent particle permeabilities (14,27) similar to mucus. As diffusing particles, we employ fluorescently labeled dextran molecules of varying sizes. When using confocal laser-scanning fluorescence microscopy to investigate particle penetration into hydrogels, the sample can be oriented such that the hydrogel-bulk interface is either parallel (16) or perpendicular (28) to the optical axis, which makes no significant difference from a scanning perspective. However, for laterally extended samples like cell cultures that grow on a substrate, the parallel alignment causes the light path to span substantially larger distances, making this setup more prone to distortions in the imaging process. A perpendicular alignment, as employed in this work and sketched in Fig. 1, is therefore preferable for biological samples (28) and is also compatible with future extensions of such penetration assays to mucus-producing cell cultures.

We investigate the filtering function of hydrogels by theoretical analysis of time-resolved concentration profiles of the labeled dextran molecules as they penetrate into the hydrogel. The employed numerical method allows for simultaneous extraction of free-energy and diffusivity profiles from relative concentration profiles at different times and is a significant extension of earlier methods (29–31) because it does not require absolute concentration profiles but works with relative, i.e., arbitrarily normalized, concentrations. This is a crucial advantage because often fluorescence intensity profiles are subject to significant perturbation due to, e.g., laser light intensity fluctuations or fluorescence dye bleaching over the course of the experiment, and using relative concentrations makes the often-difficult conversion of measured intensity data into absolute particle concentrations obsolete. The analysis framework we introduce here can thus be used for a wide range of experimental setups to simultaneously extract free-energy and diffusivity profiles from a variety of different biological systems. As a check on the robustness of the method, the extracted dextran bulk diffusivities are shown to agree well with fluorescence correlation spectroscopy (FCS) data that are obtained separately. The obtained particle free energies and diffusivities inside the hydrogel are shown to obey empirical scaling laws as a function of the dextran mass. The dextran free energy inside the hydrogel is described by a free-volume model based on repulsive steric interactions between the dextran molecules and the hydrogel linkers, which includes dextran as well as hydrogel linker flexibility. This model constitutes a modified size-filtering mechanism for repulsive particle-hydrogel interactions, according to which particle penetration into hydrogel pores is assisted by the elastic widening of pores and the elastic shrinking of dextran molecules and matches the extracted particle free energies in the hydrogel quantitatively. The model furthermore suggests that the hydrogel mesh-size distribution is rather broad and that particle penetration is dominated by the fraction of large pores in the hydrogel.

MATERIALS AND METHODS

Hydrogel preparation

The hydrogel is formed by cross-linking end-functionalized polyethylene-glycol-bicyclo[6.1.0]non-4-yne (PEG-BCN) linkers with hyperbranched polyglycerol azide (hPG-N_3) hubs via strain-promoted azide-alkyne cycloaddition. The two macromonomers PEG-BCN and hPG-N_3 are synthesized as previously described (2,32). The "click" reaction of binding the PEG-BCN linkers to the hPG-N_3 hubs works in water, at room temperature, without the addition of a catalyst or external activation like heat or ultraviolet radiation and without the formation of byproducts. Two different sizes of PEG-BCN linkers are employed, having a molecular weight of either $M_{PEG} = 6$ or $M_{PEG} = 10$ kDa (for details about the mass distributions,

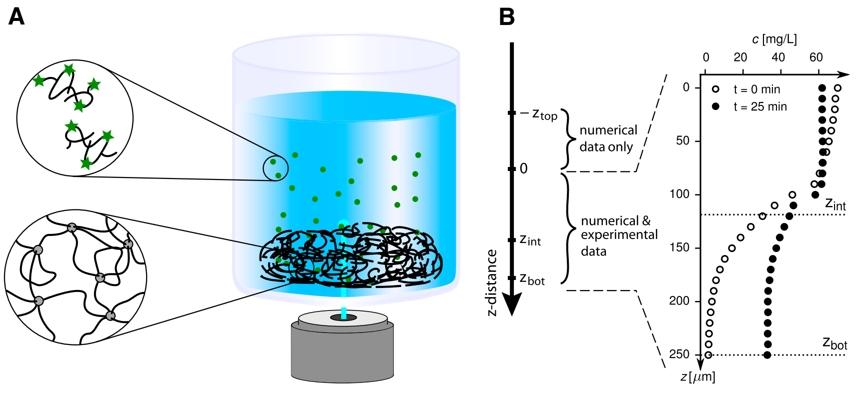

FIGURE 1 (*A*) Schematic drawing of the experimental setup. Concentration profiles of fluorescently labeled dextran molecules (*green*) are measured as they penetrate from the bulk solution (*blue*) into the hydrogel (*black*). The origin of the z axis is positioned such that experimentally measured profiles range from $z = 0$ to $z = z_{\text{bot}}$. The hydrogel-bulk solution interface is located at $z = z_{\text{int}}$. In the range from $z = -z_{\text{top}}$ to $z = 0$, only numerically determined concentration profiles are available. (*B*) Exemplary experimental concentration profiles for two different penetration times for $M_{\text{dex}} = 4$ kDa dextran diffusing into the *hPG-G10* hydrogel are given; positions of the hydrogel-bulk solution interface z_{int} and the hydrogel-glass bottom interface z_{bot} are indicated. To see this figure in color, go online.

see Supporting Materials and Methods, Section S1), the hydrogels are denoted as *hPG-G6* and *hPG-G10*, respectively. The number ratio of the PEG-BCN linkers to the hPG-N_3 hubs ($M_{\text{hPG}} = 3$ kDa, 20% azide) is kept constant at 3:1 for both *hPG-G6* and *hPG-G10*. This ratio can ideally lead to a cubic lattice structure if each hPG-hub exactly binds to six PEG linkers. The chemical structure of the hPG-N_3 hubs, however, allows on average for eight binding sites, making the hydrogel presumably quite disordered.

The two components of the hydrogel are stored as aqueous stock solutions at concentrations of 8.5 wt% (6 kDa PEG-BCN), 8.4 wt% (10 kDa PEG-BCN), and 5 wt% (hPG-N_3). After very long storage times of the stock solutions of about 1 year, the cross-linking click reaction of PEG linkers and hPG hubs starts to become impaired, which is why storage times are kept short. To minimize aging effects of the hydrogels, hydrogel formation is always initiated shortly before the start of the experiments by mixing the components according to Table 1. The resulting gel solution is thoroughly vortexed before being placed as 1 μL drops on the glass substrate. Both hydrogel solutions are adjusted to have the same mass concentration. However, after drying and reswelling on the glass substrate, volumes of the formed hydrogels are different and measured as $V_{\text{tot}}^{\text{hPG-G6}} = 0.42 \pm 0.03$ μL and $V_{\text{tot}}^{\text{hPG-G10}} = 0.31 \pm 0.04$ μL for *hPG-G6* and *hPG-G10*, respectively (see Fig. S1 in Supporting Materials and Methods, Section S2). This results in a final hydrogel concentration of 9 wt% (~90 mg/mL) for *hPG-G6* and 12 wt% (~120 mg/mL) for *hPG-G10*.

Estimate of mean hydrogel mesh size

Assuming an idealized cubic hydrogel network structure, the mean mesh size can be easily estimated. The length of a cubic unit cell $l_{0,\text{ideal}}$ follows from the total gel volume V_{tot} and the total number of hPG hubs $n_{\text{hPG}}^{\text{tot}}$ in mol as

$$l_{0,\text{ideal}} = \sqrt[3]{\frac{V_{\text{tot}}}{n_{\text{hPG}}^{\text{tot}} N_{\text{A}}}}, \tag{1}$$

where N_{A} is the Avogadro constant. The total volumes for the rehydrated gels are $V_{\text{tot}}^{\text{hPG-G6}} = 0.42$ μL and $V_{\text{tot}}^{\text{hPG-G10}} = 0.31$ μL as mentioned above. The total number of hPG hubs is given as $n_{\text{hPG}}^{\text{tot}} = n_{\text{hPG}} \times V_{\text{app}}/V_{\text{gel}}^{\text{sol}}$, with the values from Table 1 for the respective gel and in which we account for the fact that only $V_{\text{app}} = 1$ μL of the total gel solution $V_{\text{gel}}^{\text{sol}}$ is applied onto the gel substrate. This results in rough estimates for the mesh size of $l_{0,\text{ideal}}^{\text{hPG-G6}} = 7.1$ nm and $l_{0,\text{ideal}}^{\text{hPG-G10}} = 7.5$ nm, which shows that even though PEG linkers of significantly different masses were used, the mesh sizes of the two gels differ only slightly. In deriving Eq. 1, one assumes an ideal hydrogel pore connectivity that corresponds to a perfect cubic lattice. There is no reason why the hydrogel should consist of a perfect cubic lattice; on the contrary, entropy favors a disordered network topology. For cubic pores with lower connectivity, Fig. 2 illustrates how the pore size l_0 can increase for a fixed PEG end-to-end distance R_{PEG}. Thus, except for the case of an ideal cubic lattice, the pore size l_0 will be larger than the estimate of Eq. 1, as indeed suggested by our elastic free-volume model.

Dextran preparation

Dextrans conjugated with the dye fluorescein isothiocyanate (FITC) are obtained from *Sigma-Aldrich* as d4-FITC, d10-FITC, d20-FITC, d40-FITC, and d70-FITC, the number stating the molecular weight in kDa of the commercial product. To remove unbound FITC from the dextran solutions, all batches are subjected to a desalting PD-10 column, which eliminates low-molecular weight compounds such as free FITC dye. This step is done according to the manufacturer's recommendations, and the column is equilibrated using phosphate-buffered saline (PBS). Afterwards, the molecular weight distribution of all dextrans is determined by gel permeation chromatography (see Supporting Materials and Methods, Section S1).

Penetration assay of FITC-labeled dextrans

After preparation of the hydrogel solutions and purification of the dextrans (see above), penetration assays are performed with five different dextran solutions and two different gels. For these assays, coverslips (Menzel #1;

TABLE 1 Composition of the Hydrogels Used in this Study

	n_{PEG}	$V_{\text{PEG}}^{\text{sol}}$ [a]	n_{hPG}	$V_{\text{hPG}}^{\text{sol}}$ [b]	V_{H_2O}	$V_{\text{gel}}^{\text{sol}}$	V_{app}	m_{app}	n_{app}
hPG-G6	142 nmol	10 μL	47 nmol	2.8 μL	13.0 μL	25.8 μL	1 μL	38 μg	7.3 nmol
hPG-G10	84 nmol	10 μL	28 nmol	1.7 μL	12.7 μL	24.4 μL	1 μL	38 μg	4.6 nmol

Here, $V_{\text{PEG}}^{\text{sol}}$ and $V_{\text{hPG}}^{\text{sol}}$ denote the volumes of the stock solutions, V_{H_2O} is the volume of purified water added to the resulting gel solutions, and n_{PEG} and n_{hPG} denote the amount of PEG linkers and hPG hubs in the gel solutions. From the total resulting volume of the gel solutions $V_{\text{gel}}^{\text{sol}}$, only $V_{\text{app}} = 1$ μL was placed as a gel spot on the glass substrate, leading to the combined applied amount n_{app} and the combined applied mass m_{app} of PEG linkers and hPG hubs.
[a]Solution is of 8.5 wt% for 6 kDa PEG and 8.4 wt% for 10 kDa PEG.
[b]hPG solution is of 5 wt%.

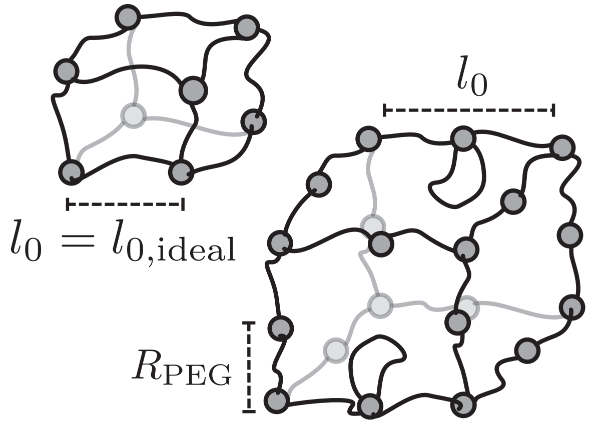

FIGURE 2 A cubic pore with lower connectivity to the right, containing two PEG linkers per edge instead of one, leads to an effectively larger unit-cell length l_0 at the same PEG end-to-end distance R_{PEG}. Only for a perfect cubic lattice to the left is the estimate of Eq. 1 valid and $l_0 = l_{0,\text{ideal}} = R_{\text{PEG}}$.

VWR, Darmstadt, Germany) with a diameter of 25 mm and a thickness of 0.13–0.16 mm are thoroughly washed with water and absolute ethanol and subsequently dried under a stream of nitrogen. For every experiment, 1 μL of the respective hydrogel solution is placed on the center of the coverslip. The substrates with the applied gel spots are kept in a humid environment overnight, allowing hydrogel formation to be completed before the hydrogel spots are left to dry for 30 min at ambient conditions. Permeation experiments are performed within 1 day after hydrogel formation. To start a permeation experiment, a home-made polydimethylsiloxane stamp (1 $\times$ 1 cm) prepared with a cylindrical cavity in the middle (5 mm diameter) is placed on the coverslip, so that the dried hydrogel is located in the middle of the stamp's cavity. The polydimethylsiloxane surrounding the dried hydrogel allows for the addition of solutions such as buffer or dextran. Before the measurement, 30 μL of PBS buffer are added to reswell the hydrogel for 30 min, which typically creates hydrogel volumes of semispheroid shape with a base radius of 1050 μm and heights of $\sim$150 μm for *hPG-G10* and $\sim$210 μm for *hPG-G6* (see Supporting Materials and Methods, Section S2). Afterwards, the coverslip is mounted on a Leica SP8 confocal laser-scanning microscope (CLSM; Leica, Wetzlar, Germany) and imaged using a 20$\times$ objective (0.75 HC PL APO water immersion objective with correction ring). In a first step, the hydrogel is visually identified by imaging the sample with a 488 nm laser and collecting the transmitted light using the transmission photomultiplier tube of the CLSM, allowing us to place the optical axis of the CLSM in the center of the hydrogel and to place the focal plane 30 μm below the glass-hydrogel interface. After aligning the sample like this, the PBS buffer is removed from the cavity and replaced by 35 μL of the FITC-dextran solution (0.07 mg/mL for all dextrans). This fixes the total length from the bottom of the glass dish at $z = z_{\text{bot}}$ to the air-water interface at $z = z_{\text{top}}$, where $z = 0$ corresponds to the end of the measurement region (see Fig. 1 *A*). The total length of the solution is thus $z_{\text{tot}} = z_{\text{top}} + z_{\text{bot}} = 1780$ μm. The individual contributions to z_{tot} vary because of different gel thicknesses changing the extent of the measured region, ranging from $z = 0$ to $z = z_{\text{bot}}$ (cf. also Fig. 1 *A*).

About 10 s after the application of the dextran solution, the spatial distribution of the FITC-based fluorescence intensity is measured using a z-stack that starts 30 μm below and ends 410 μm above the glass-hydrogel interface (with 10 μm increments). The recorded intensities are afterwards truncated to probe the spatial FITC distribution within the hydrogel starting from the glass bottom (located at z_{bot}) and extending $\sim$100 μm into the bulk solution, away from the gel-water interface located at $z = z_{\text{int}}$ (cf. Fig. 1 *A*). In these measurements, the sample is excited at $\lambda = 488$ nm, and the emission is recorded between 500 and 550 nm using a photomultiplier tube. For the $M_{\text{dex}} = 4$ kDa to the $M_{\text{dex}} = 40$ kDa dextrans, one z-stack is recorded every $\Delta t = 10$ s, yielding time-resolved FITC distributions after the penetration of the dextran molecules into the hydrogel network over time. For the $M_{\text{dex}} =$ 70 kDa dextrans, a period of $\Delta t = 30$ s is used instead to account for the much smaller diffusion coefficient of the larger dextran molecules. The employed temporal resolutions can be easily estimated to be larger than timescales on which effects of anomalous diffusion are present; for diffusion over length scales larger than the mesh size of the hydrogel, normal diffusion is expected. An upper bound for the corresponding crossover timescale can be estimated as $\tau = l_0^2/D_{\text{gel}}$, where $l_0 = 24$ nm is an upper estimate for the hydrogel mesh size and $D_{\text{gel}} = 0.15$ μm^2/s is the smallest obtained diffusion constant in the hydrogel (see below for explicit results). The resulting value of $\tau \approx 0.2$ ms, beyond which normal diffusion is expected, is several orders of magnitude lower than the experimental temporal resolution. Thus, anomalous diffusion cannot be observed in the experimental data, and the normal diffusion equation that is used to model the time-dependent experimental concentration profiles should be valid.

For all dextran types, measurements are performed at least three times with total measurement times of $\sim$30 min, with the exception of the $M_{\text{dex}} = 70$ kDa dextrans. Here, only one measurement is performed for each gel, but with a longer recording time of $\sim$1 h.

FCS of FITC-labeled dextrans

Reference diffusion coefficients for the FITC-labeled dextran molecules in the bulk solution are obtained using FCS. The measurements are performed on a Leica TCS SP5 II CLSM with an FCS setup from PicoQuant (Berlin, Germany). The CLSM is equipped with an HCX PL APO 63$\times$/1.20 W CORR CS water immersion objective. Samples are put on high-precision cover glasses (18 $\times$ 18 mm, 170 $\pm$ 5 μm thick) and excited with the 488 nm Argon laser line. The fluorescent light is passed through a 50/50 beam splitter with a lower wavelength cutoff of $\lambda = 515$ nm. Both channels are detected separately with a single photon avalanche diode. Afterwards, a pseudo-cross correlation is performed between both channels to eliminate the influence of detector afterpulsing. Before a measurement, the optical setup is calibrated with the water-soluble Alexa-Fluor 488 dye. The correlated signal is fitted with two components and accounting for triplet states. The first component is fixed to a freely diffusing FITC dye molecule for which only the fraction is a fit parameter. The second component is set to a log-normal distributed species. The component fractions and means of distribution are fitted, and the width of distribution is taken from previously performed gel permeation chromatography measurements (for details about the fitting procedure, see Supporting Materials and Methods, Section S3). The fitted diffusion times are used to calculate the diffusion coefficients and hydrodynamic radii using the Stokes-Einstein relation.

Numerical model and discretization

Extending a previously introduced method (29–31), spatially resolved diffusivity and free-energy profiles are estimated from experimentally measured concentration profiles. Numerical profiles are computed by discretizing the entire experimental setup from the glass bottom of the substrate to the air-water interface (z_{bot} to $-z_{\text{top}}$ in Fig. 1 *A*). In the regime in which concentration profiles are measured ($z = 0$ to $z = z_{\text{bot}}$), the experimental resolution is used as the numerical discretization width $\Delta z = 10$ μm. For the range without experimental data ($z = 0$ to $z = -z_{\text{top}}$), in total, six bins are employed. Two of those bins are spaced with $\Delta z =$ 10 μm; for the other four bins, discretization spacings between $\Delta z = 300$ and 400 μm are used, depending on the z-length measured in the respective experiment z_{bot}. The z-dimension of the total system is the same for all experiments and given as $z_{\text{tot}} = z_{\text{top}} + z_{\text{bot}} = 1780$ μm. The experimentally measured region always extends from the glass bottom through the gel and at least 100 μm into the bulk solution, away from the hydrogel-bulk interface, which leads to values of $z_{\text{bot}} \approx 300$ μm, depending on the exact thickness of the hydrogel in the respective measurement.

The numerical optimization problem is given by the cost function, which is defined as

$$\sigma^2\left(D,F,\vec{f}\right) := \frac{1}{N \times M} \sum_{j=1}^{N} \sum_{i=1}^{M} \left[c_i^{\text{num}}\left(t_j\right) - f_j \times c_i^{\text{exp}}\left(t_j\right)\right]^2, \tag{2}$$

with N the total number of experimental profiles, M the total number of experimental data points per concentration profile and $\sigma^2(D, F, \vec{f})$ being the mean squared deviation between the experimental and numerical profiles. The diffusivity profile $D = D(z)$, the free-energy landscape $F = F(z)$, and the vector containing all scaling factors (see below for details) $\vec{f} = (f_1, \ldots, f_j, \ldots, f_N)$ are all optimized to find the minimal value of σ^2. This nonlinear regression is performed using the trust region method implemented in Python's *scipy* package (33).

The numerical profiles

$$\vec{c}_{\text{num}}\left(t_j\right) = \left(c_1^{\text{num}}\left(t_j\right), \ldots, c_i^{\text{num}}\left(t_j\right), \ldots, c_M^{\text{num}}\left(t_j\right)\right)^T$$

are computed from the diffusivity and free-energy profiles as

$$\vec{c}_{\text{num}}\left(t_j\right) = e^{Wt_j} \times \vec{c}_{\text{init}}, \tag{3}$$

where the rate matrix $W(D, F)$ is defined as

$$W_{i,k} = \frac{D_i + D_k}{2\Delta z^2} e^{-\frac{F_i - F_k}{2k_B T}}, \quad \text{with} \quad k = i \pm 1$$

as explained previously (29). Numerical profiles at time t_j depend on the initial profile $\vec{c}_{\text{init}}$ at $t = 0$, which is determined as explained below.

The numerically computed profiles are fitted to the rescaled experimental profiles $\vec{c}_{\text{exp}}(t_j)$ at time $t_j > 0$. The scaling factors $\vec{f}$ are obtained simultaneously from the fitting procedure and correct drifts in the experimentally measured fluorescence intensity profiles (see Supporting Materials and Methods, Section S4). As a check, the numerical model is compared to the analytical solution for a model with piecewise constant values of the diffusivity and free energy in the respective regions. Results from the numerical model agree perfectly with those from the analytical solution (see Supporting Materials and Methods, Section S5).

Construction of the initial concentration profile

The initial profile $\vec{c}_{\text{init}}$, used for the computation of all later profiles according to Eq. 3, needs to cover the entire computational domain and is generated by extending the first experimentally measured profile $\vec{c}_{\text{exp}}$ $(t = 0)$ into the bulk regime (from $z = 0$ to $z = -z_{\text{top}}$, cf. Fig. 1 *A*). We define $t = 0$ as the time of the first measurement, which is performed ~10 s after application of the dextran solution onto the gel-loaded substrate. For the spatial extension of the profile, a constant initial concentration is assumed in the bulk, the value of which is taken as the experimentally measured value furthest into the bulk $c_0 := c_1^{\text{exp}}$ $(t = 0)$ at $z = 0$. This leads to the following expression used for the initial profile

$$c_i^{\text{init}} := \begin{cases} c_0, & \text{if } -z_{\text{top}} \le z_i \le 0 \\ c_i^{\text{exp}}(t = 0), & \text{if } 0 < z_i \le z_{\text{bot}} \end{cases}, \tag{4}$$

which by construction is continuous at $z = 0$. The initial profiles used for the fit procedure are shown in Fig. 3, *B* and *F* as black lines. To obtain concentration profiles in physical units, we set the first measured value furthest into the bulk equal to the applied dextran concentration $c_0 = 70$ mg/L.

Free-energy and diffusivity profiles

Because the experimental system consists of two regions, namely the hydrogel and the bulk solution, and to reduce the number of parameters of the numerical model to avoid overfitting, we employ sigmoidal profiles for the diffusivity $D(z)$ and free energy $F(z)$, which transition continuously from the value in the bulk solution to their values in the hydrogel. This sigmoidal shape is modeled using the following expressions:

$$D(z) = \frac{D_{\text{sol}} + D_{\text{gel}}}{2} + \frac{D_{\text{sol}} - D_{\text{gel}}}{2} \operatorname{erf}\left(\frac{z - z_{\text{int}}}{\sqrt{2} d_{\text{int}}}\right),$$

$$F(z) = \frac{\Delta F_{\text{gel}}}{2} + \frac{\Delta F_{\text{gel}}}{2} \operatorname{erf}\left(\frac{z - z_{\text{int}}}{\sqrt{2} d_{\text{int}}}\right), \tag{5}$$

where $\operatorname{erf}(z) := 1/\sqrt{\pi} \int_{-z}^{z} e^{-z'^2} dz'$ is the error function. The fit parameters z_{int} and d_{int} determine the transition position and width, respectively, and are the same for the free-energy and diffusivity profiles. Because only free-energy differences carry physical meaning, the free energy in the bulk solution is set to zero so that $F_{\text{sol}} = 0$. The values of the diffusivity and free energy in the hydrogel and in the bulk solution are thus determined by fitting the five parameters of Eq. 5, namely D_{gel}, ΔF_{gel}, D_{sol}, z_{int}, and d_{int}, to the experimentally measured concentration profiles.

Confidence intervals for the obtained parameters of D_{sol}, D_{gel}, and ΔF_{gel} are estimated by determining the parameter values that change σ by not more than 50% (for details, see Supporting Materials and Methods, Section S6). The error bars shown in Fig. 5 are then obtained by averaging the confidence intervals over all measurements.

RESULTS AND DISCUSSION

Fluorescence intensity profiles of FITC-labeled dextran molecules penetrating into PEG-based hydrogels are analyzed using the procedure explained in the Materials and Methods. The analysis is based on numerical solutions of the one-dimensional generalized diffusion equation (35)

$$\frac{\partial c(z,t)}{\partial t} = \frac{\partial}{\partial z}\left[D(z) e^{-\beta F(z)} \frac{\partial}{\partial z}\left(c(z,t) e^{\beta F(z)}\right)\right], \tag{6}$$

where $c(z, t)$ is the concentration at time t and depth z (see Fig. 1), $D(z)$ and $F(z)$ are the spatially resolved diffusivity and free-energy profiles that the dextran molecules experience, and $\beta = 1/k_B T$ is the inverse thermal energy. Whereas the diffusivity $D(z)$ describes the mobility of dextran molecules at position z, the free-energy profile $F(z)$ uniquely determines the equilibrium partitioning of dextran molecules. The numerical solution of Eq. 6 provides a complete model of the penetration process into the hydrogel and at the same time allows for extraction of the diffusivity and free-energy profiles by comparison with experimentally measured concentration profiles. A direct conversion of measured fluorescence intensities into absolute concentrations is often difficult because of drifts of various kinds. The method developed here circumvents this problem and allows for in-depth analysis of arbitrarily normalized concentration profiles, as explained in Numerical Model and Discretization. Complete profiles of free energies and diffusivities,

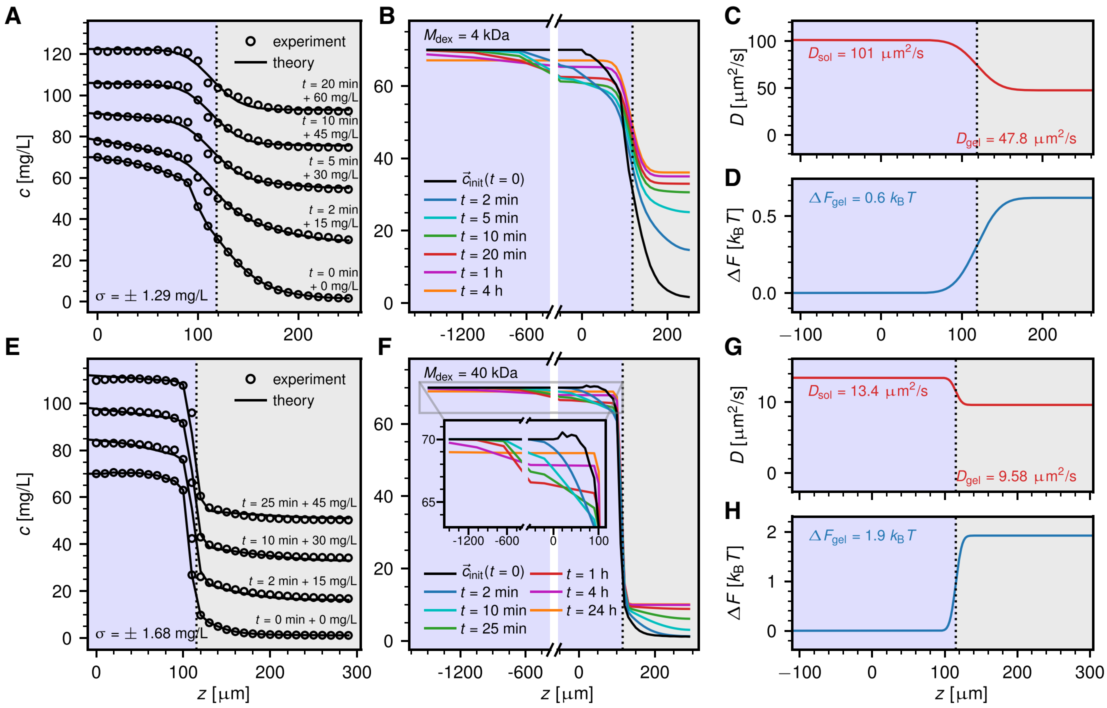

FIGURE 3 Exemplary time-dependent dextran concentration profiles from experimental measurements (*circles*) and numerical modeling (*solid lines*) for the *hPG-G10* hydrogel. Results for the smallest dextran with $M_{dex} = 4$ kDa in (*A*)–(*D*) are compared with results for $M_{dex} = 40$ kDa in (*E*)–(*H*). (*A* and *E*) Experimental and modeled concentration profiles agree very accurately; note that concentration profiles are shifted vertically for better visibility. The initial bulk concentration of dextran is $c_0 = 70$ mg/L. (*B* and *F*) Modeled concentration profiles are presented for a wide range of penetration times. The initial profile $\vec{c}_{init}$ (*black line*) is based on experimental data (see Construction of the Initial Concentration Profile). (*C* and *G*) Extracted diffusivity profiles are given, showing that the diffusivity in the hydrogel is only slightly reduced compared to the bulk solution. (*D* and *H*) Extracted free-energy profiles are shown. Significant exclusion of dextran from the hydrogel is observed, with a stronger effect for the larger dextran. To see this figure in color, go online.

both in the bulk and in the PEG hydrogel, are obtained, and the results for different hydrogels and dextran molecules of varying sizes will be analyzed in the following.

Comparison between experimental and modeled concentration profiles

Fig. 3, *A* and *E* shows exemplary concentration profiles for dextran molecules with molecular masses of $M_{dex} = 4$ kDa and $M_{dex} = 40$ kDa penetrating into the *hPG-G10* hydrogel (see Hydrogel Preparation). Measurements are performed over a total time span of ~30 min, and concentration profiles are recorded every 10 s, leading to a total of ~180 concentration profiles as input for the numerical extraction of the diffusivity and free-energy profiles. The first measured concentration profile at $t = 0$ min represents the start of the experiment, ~10 s after the dextran solution was applied onto the gel (see Penetration Assay of FITC-Labeled Dextrans). The numerically determined concentration profiles (*lines*) reproduce the experimental data (data points) very accurately, as seen in Fig. 3, *A* and *E*. The deviation is estimated from the normalized sum of residuals, σ (according to Eq. 2), which is below 2 mg/L for both measurements. A stationary concentration profile is obtained in the theoretical model only after 4 h of penetration for the smaller 4 kDa dextran (see Fig. 3 *B*); for the larger dextran molecule, the stationary profile is reached only after an entire day (see Fig. 3 *F*). These times significantly exceed the duration of the experiments.

The extracted diffusivity and free-energy profiles in Fig. 3, *C*, *D*, *G*, and *H* reveal the selective hydrogel permeability for dextran molecules of varying size. The free-energy difference in the hydrogel is positive $\Delta F_{gel} > 0$ for both dextran sizes, indicating that dextran is repelled from the hydrogel. The dextran partition coefficient K_{gel} between the hydrogel and the bulk solution is related to the change in the free energy ΔF_{gel} as

$$K_{gel} = e^{-\beta \Delta F_{gel}}. \tag{7}$$

According to Eq. 7, the obtained free-energy differences $\Delta F_{gel} = 0.6\ k_B T$ and $\Delta F_{gel} = 1.9\ k_B T$ correspond to partition coefficients of about $K_{gel} \approx 1/2$ and $K_{gel} \approx 1/7$ for the smaller and the larger dextran molecules, respectively, which illustrates a significant exclusion in particular for the larger dextran. Compared with the partition coefficients, the diffusion constants in the hydrogel decrease only slightly as a function of the dextran mass. This suggests

that the dextran molecules are only modestly hindered in their motion, a conclusion that will be rationalized by our elastic free-volume model further below.

Fig. 4 shows the temporal evolution of the average dextran concentration $\bar{c}$ in three different regions, namely inside the gel for $z_{\mathrm{int}} < z < z_{\mathrm{bot}}$, in the near solution for $0 < z < z_{\mathrm{int}}$, and in the far solution for $-z_{\mathrm{top}} < z < 0$ for the same data shown in Fig. 3. The lines show the predictions based on the extracted diffusivity and free-energy profiles and the circles the experimental data, which are not available in the far solution range. The average concentration in the gel (*black*) increases monotonically and saturates after about 1 h for both dextran sizes. Note that the stationary final concentration in the hydrogel is considerably less for the larger dextran with $M_{\mathrm{dex}} = 40$ kDa. In contrast, the average concentration in the far solution saturates more slowly and shows a slight nonmonotonicity for both dextran masses (*blue*). This nonmonotonicity is more pronounced in the near solution (*red*) and is caused by the fact that dextran molecules diffuse quickly into the hydrogel from the near solution in the beginning of the experiment, whereas the replenishment from the bulk solution takes a certain time, as also seen in the concentration profiles in Fig. 3, *B* and *F*. Very good agreement between experiments and modeling results is observed.

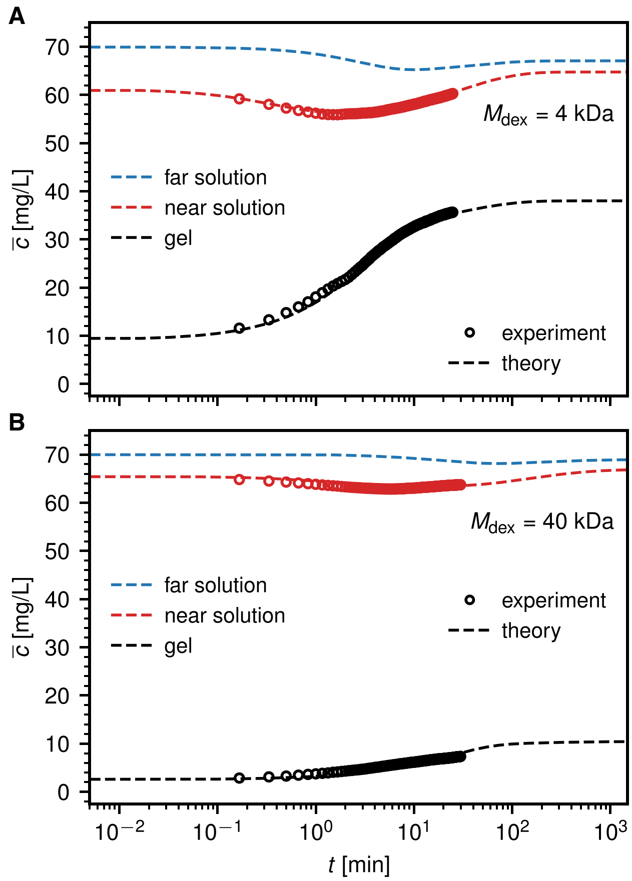

FIGURE 4 Comparison of experimental results (*circles*) and modeling results based on the extracted diffusivity and free-energy profiles (*lines*) for the mean dextran concentration $\bar{c}$ over time in three different regions, the far solution ($-z_{\mathrm{top}} < z < 0$), the near solution ($0 < z < z_{\mathrm{int}}$), and the gel ($z_{\mathrm{int}} < z < z_{\mathrm{bot}}$); see Fig. 1. The systems are the same as shown in Fig. 3. A nonmonotonic dextran concentration is measured over time in the near and far solution regions. The fact that $\bar{c}$ in the gel does not vanish for $t \rightarrow 0$ reflects that the first measurement at $t = 0$ is done ~10 s after the application of the dextran solution onto the gel. The initially employed bulk dextran concentration is $c_0 = 70$ mg/L. To see this figure in color, go online.

Influence of dextran size on hydrogel penetration

The same analysis is performed for dextran molecules of molecular masses ranging from $M_{\mathrm{dex}} = 4$ kDa to $M_{\mathrm{dex}} = 70$ kDa that penetrate into PEG hydrogels with two different linker lengths, namely *hPG-G6* with a PEG linker size of $M_{\mathrm{PEG}} = 6$ kDa and *hPG-G10* with $M_{\mathrm{PEG}} = 10$ kDa. Fig. 5 shows the extracted diffusivities and free energies, which result from averages over at least three experiments for each system, except for $M_{\mathrm{dex}} = 70$ kDa dextran, for which only one experiment was performed.

Fig. 5 *A* shows the bulk diffusivities D_{sol} extracted from measured concentration profiles as colored symbols; in principle, there should be no difference between results for *hPG-G6* and *hPG-G10*. A power-law relation between the dextran mass and the diffusivity according to $D_{\mathrm{sol}} \propto M_{\mathrm{dex}}^{-\nu}$ is shown as straight lines for $\nu = 1$ (*broken line*) and for $\nu = 1/2$ (*dotted line*). An exponent of $\nu = 1/2$ agrees nicely with our FCS data (*solid black triangles*; see FCS of FITC-Labeled Dextrans) as well as with literature fluorescence recovery after photobleaching (FRAP) measurements (34) (*open black triangles*). The value $\nu = 1/2$ follows from combining the generally applicable Stokes-Einstein relation $D_{\mathrm{sol}} = k_B T / 6\pi\eta_w r_0$ (36) with the scaling of the dextran hydrodynamic radius according to $r_0 \propto M_{\mathrm{dex}}^{\nu}$ (37,38) by assuming that the bulk solution is a theta solvent for dextran polymers (39,40) (see Supporting Materials and Methods, Section S7 for details). The exponent $\nu = 1/2$ is only expected for linear polymers, whereas dextran is in fact a branched polymer. The good agreement of FCS and FRAP data with the power law for $\nu = 1/2$ suggests that the degree of branching is low (41) or that branching effectively compensates self-avoidance effects. The dextran hydrodynamic radii estimated from the FCS measurements compare well with the values reported by the supplier (see Table 2). The data for D_{sol} obtained from the time-dependent dextran concentration profiles show rather large uncertainties, which is due to the fact that the concentration profiles are rather insensitive to the bulk diffusivities; they are within error bars consistent with our FCS results but do not allow extraction of the power-law scaling with any reasonable confidence.

Values for the diffusion constant in the hydrogel D_{gel} are compared with power laws with exponents $\nu = 1/2$ and $\nu = 1$ in Fig. 5 *B*. The difference of the diffusion constants between the two different hydrogels is within the error bars,

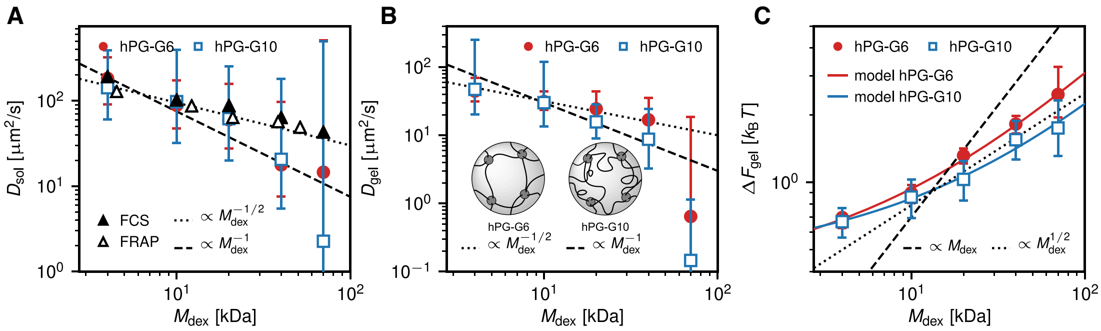

FIGURE 5 Results for the diffusivity and free energy obtained from the experimental measurements as a function of dextran mass. (*A*) Fitted diffusivities in the bulk solution (*squares* and *circles*) agree within the error with FCS data measured in this work (*solid black triangles*) and with FRAP measurements from literature (34) (*open black triangles*). (*B*) Fitted diffusivities in the hydrogel are reduced relative to the bulk values and are compared to different power laws. (*C*) Dextran molecules are excluded from the hydrogel and $\Delta F_{gel} > 0$ for all dextran masses. For larger dextran molecules, ΔF_{gel} increases as a square root with the dextran mass. The results from the free-volume model of Eq. 12 (*continuous lines*) agree nicely with the measurements. Error bars have been estimated as explained in Supporting Materials and Methods, Section S6. The inset in (*B*) presents a schematic depiction of the two different gels. Even though the *hPG-G10* gel is composed of larger linkers, the mass density is larger than in the *hPG-G6* gel, which results in an effectively smaller pore size. To see this figure in color, go online.

which reflects the fact that the estimated mean hydrogel mesh sizes, using a very simplistic hydrogel network model with a perfect cubic structure, are $l_{0,ideal}^{hPG-G6} = 7.1$ nm and $l_{0,ideal}^{hPG-G10} = 7.5$nm (see Estimate of Mean Hydrogel Mesh Size) and thus quite similar to each other. It is to be noted that for $M_{dex} \leq 20$ kDa, the estimated mesh sizes are larger than twice the dextran hydrodynamic radii from Table 2, which would not suggest any dramatic confinement effect on the diffusion constant (42). Interestingly, for the data for which $M_{dex} \gtrsim 20$ kDa, the hydrogel with the larger linker length (*hPG-G10*), which has a slightly higher mesh size, is seen to reduce the diffusion constant slightly more, which at first sight is counterintuitive. This finding can be rationalized by the fact that the *hPG-G10* gel has a higher mass density compared to the *hPG-G6* gel (see Hydrogel Preparation), and thus, the effective pore size is presumably substantially smaller. This is schematically illustrated in the inset in Fig. 5 *B*. A diffusivity scaling with an exponent $\nu = 1$, which describes the data for *hPG-G10* slightly better, could be rationalized by screened hydrodynamic interactions or by reptation-like diffusion (43). In fact, a crossover in the scaling of the diffusivity with increasing hydrogel density from $\nu = 1/2$ to $\nu = 1$ has been described before for dextran penetrating into hydroxypropyl cellulose (38). However, because of the large error bars, extraction of the diffusivity scaling with respect to dextran mass in the two gels is not uniquely possible. This is mostly due to the fact that the diffusivities change rather mildly with varying dextran mass. This is why we do not attempt to model the scaling of the extracted diffusivities, as was done elsewhere before (18,19,44), but rather focus on the mechanism behind the extracted free-energy differences in the following.

TABLE 2 Dextran Radii

M_{dex}	r_0	r_{FCS}
4 kDa	1.4 nm	1.5 nm
10 kDa	2.3 nm	2.7 nm
20 kDa	3.3 nm	3.2 nm
40 kDa	4.5 nm	4.3 nm
70 kDa	6.0 nm	6.4 nm

Hydrodynamic radius r_0 as reported by the supplier, in comparison to estimated hydrodynamic radius r_{FCS} based on our FCS measurements using the Stokes-Einstein relation and the viscosity of water as $\eta_w = 0.8 \times 10^{-3}$ Pa s.

Fig. 5 *C* shows the extracted values of ΔF_{gel} for the two hydrogels as a function of the dextran mass. In all measurements, we find $\Delta F_{gel} > 0$, which suggests exclusion of the dextran molecules from the hydrogel. Also, the value of ΔF_{gel} increases with the dextran mass. Because dextran, as well as the PEG-hPG based hydrogels, is uncharged (45), this exclusion must be due to steric repulsion, possibly enhanced by hydration repulsion (46,47).

Elastic free-volume model for dextran penetration in hydrogels

For the larger dextran molecules, the hydrogel with the smaller PEG linkers, *hPG-G6*, displays a slightly stronger exclusion. The power-law relation between the hydrogel free energy and dextran mass according to $\Delta F_{gel} \propto M_{dex}^{\alpha}$ with an exponent of $\alpha = 1/2$ describes the data well for larger dextran masses $M_{dex} \gtrsim 20$ kDa, as shown by the dotted black line in Fig. 5 *C*. This power-law behavior is in fact compatible with a simplistic elastic free-volume model for the penetration of dextran molecules into hydrogels, which yields the solid lines and will be derived in the following.

The model geometry is sketched in Fig. 6 *A* and consists of a single dextran molecule of radius *r* (*green sphere*) inside a cubic unit cell of the PEG-based hydrogel (*gray cylinders*), similar to previous coarse-grained hydrogel models (18–20). The presence of the hPG hubs connecting the PEG linkers is neglected in the following. The dextran

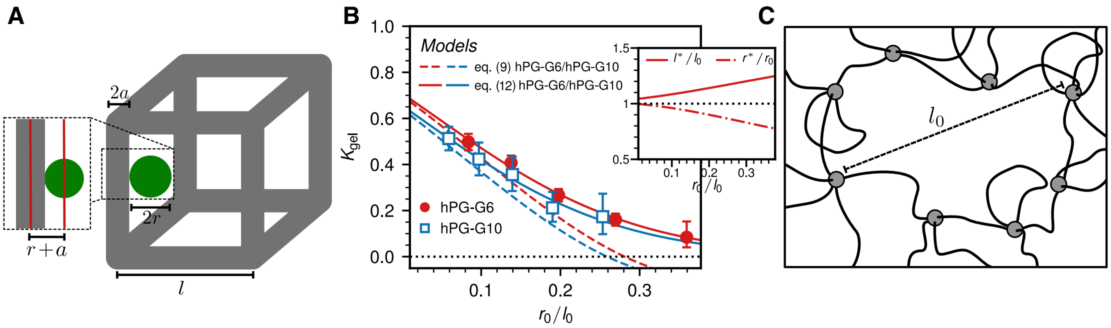

FIGURE 6 Elastic free-volume model for the partitioning of a particle in a hydrogel. (*A*) Schematic sketch of the cubic unit-cell model for the hydrogel is given, made up of connected linkers of length l and a finite radius of a. The diffusing particle is modeled as a sphere of radius r. Both the particle and the linkers are elastic and can stretch or contract. (*B*) Partition coefficient K_{gel} extracted from the experimentally measured dextran concentration profiles (*symbols*) is shown in comparison with the elastic free-volume model predictions according to Eq. 12 (*solid lines*). The results of the nonelastic model according to Eq. 9 are shown as dashed lines. Error bars have been estimated as explained in Supporting Materials and Methods, Section S6. The inset shows the equilibrium values of l^* and r^* obtained for the *hPG-G6* gel. (*C*) Illustration of a disordered pore in the hydrogel that has a mesh size l_0 and consists of more than four linkers is given (see also Fig. 2). To see this figure in color, go online.

experiences a reduction of its free volume compared with the bulk solution because of steric interactions with the PEG linkers. In the simple model geometry, the PEG linkers are located at the edges of the cubic unit cell and are modeled as impenetrable cylinders of radius a and length l. Conformational fluctuations of the PEG linkers are not treated explicitly in this model; instead, the linker length l and radius a are to be understood as average values over different confirmations of the linker chains. The excluded volume V_{ex} for dextran in the cubic unit cell consists of a quarter of each of the 12 cylinders at the edges. The accessible or free volume in the hydrogel V_{free} depends on the sum of sphere radius r and cylinder radius a and is given by

$$\begin{aligned} V_{free} &= V_{unit} - V_{ex} \\ &= l^3 - \frac{12}{4}\pi(r+a)^2 l + 2V_{cyl}. \end{aligned} \tag{8}$$

Here, $V_{unit} = l^3$ is the volume of the unit cell and $V_{cyt} = (16/3)(r+a)^3$ is the volume of two intersecting cylinders (48), which is subtracted from the excluded volume to avoid over counting of the unit-cell corners. The entropic contribution to the total free energy is given by

$$\begin{aligned} \Delta F_{vol} &= -k_B T \ln\left(\frac{V_{free}}{V_{unit}}\right) \\ &= -k_B T \ln\left(1 - 3\pi\left[\frac{r+a}{l}\right]^2 + \frac{32}{3}\left[\frac{r+a}{l}\right]^3\right). \end{aligned} \tag{9}$$

Because dextran and the PEG linkers are elastic polymers, they are both flexible and can deform. For small deformations, the polymers behave like Gaussian chains (39,40). The elastic deformation free energy for a cubic unit cell consisting of 12 equally deformed PEG linkers can be written as (for a detailed derivation, see Supporting Materials and Methods, Section S8)

$$\Delta F_{PEG} = \frac{12}{2}k_B T\left(\left[\frac{l}{l_0}\right]^2 + \frac{1-4\left[\frac{l}{l_0}\right]^2}{2+\left[\frac{l}{l_0}\right]^2}\right). \tag{10}$$

Here, l/l_0 is the relative stretching of the PEG linkers, where l_0 denotes the edge length of the unit cell in the absence of dextran molecules. The elastic deformation energy of dextran is obtained in the same fashion and reads

$$\Delta F_{dex} = \frac{3}{2}k_B T\left(\left[\frac{r}{r_0}\right]^2 + \left[\frac{r_0}{r}\right]^2 - 2\right), \tag{11}$$

where r denotes the deformed dextran radius and the unperturbed dextran radius is denoted by r_0 and is taken from Table 2. The complete free energy follows as

$$\Delta F_{gel}(r,l) = \Delta F_{vol}(r,l) + \Delta F_{PEG}(l) + \Delta F_{dex}(r). \tag{12}$$

The equilibrium free energy is given by the minimal value of this free-energy expression, obtained for the optimally stretched unit-cell length l^* and the optimal dextran radius r^*, which are determined numerically. The values of the unit-cell length l_0 and the PEG linker thickness a are adjusted by fits to the experimental data. The model results are shown in Fig. 6 *B* in terms of the partition coefficient as solid lines and compared with the experiments (*circles* and *squares*) as a function of the length ratio r_0/l_0. The inset shows the obtained equilibrium values for l^* and r^* for the *hPG-G6* gel. A considerable stretching of PEG linkers and compression of dextran are observed, which shows that elasticity effects of both PEG linkers and dextran molecules are important and cannot be neglected when estimating the free volume.

The fit to the experimental data yields $l_0^{hPG-G6} = 16.7$ nm, $l_0^{hPG-G10} = 23.7$ nm, $a_{hPG\text{-}G6} = 3.4$ nm, and $a_{hPG\text{-}G10} = 5.4$ nm. The fit values of a certainly represent an effective

PEG linker radius and include the layer of tightly bound hydration water. They are indeed, close to the respective equilibrium PEG radii $R_{PEG} = b_{fl} N_{PEG}^{3/5}/\sqrt{3}$, given as $R_{PEG}^{hPG-G6} = 4.4$ nm and $R_{PEG}^{hPG-G10} = 5.99$ nm, where $b_{fl} = 0.4$ nm denotes the Flory monomer length (49) and N_{PEG} is the respective number of PEG monomers. In fact, the free-volume model yields estimates of the number of hydration waters per PEG monomer that scatter around 8, in rough agreement with literature values (see Fig. S8; Supporting Materials and Methods, Section S9).

The fit values for the unit-cell length l_0 are significantly larger than the mean mesh size estimated based on Eq. 1, which for a perfectly ordered cubic lattice predicts $l_{0,ideal}^{hPG-G6} = 7.1$ nm and $l_{0,ideal}^{hPG-G10} = 7.5$ nm, but still considerably shorter than the PEG contour lengths $L = b_0^{PEG} N_{PEG}$, which are $L_{hPG\text{-}G6} = 48.5$ nm and $L_{hPG\text{-}G10} = 80.9$ nm, where $b_0^{PEG} = 0.356$ nm is the PEG monomer length (49). Although the large unit-cell lengths obtained from the fit to the elastic free-volume model could reflect a substantial stretching of individual PEG polymers, there is no a priori reason why the linkers should be stretched to such a considerable fraction of their contour length. We therefore rationalize this surprising result in terms of a broad distribution of pore sizes that exhibit different topologies. To illustrate this, a random pore is schematically shown in Fig. 6 *C*. Based on the 3:1 number ratio of linkers/cross-linkers in the hydrogel formulation (cf. Hydrogel Preparation and Fig. 2), a perfectly cubic lattice could form, in which each hub is connected to six different linkers. Such an ideal cubic connectivity is, of course, entropically highly unfavorable, and the connectivity distribution of hubs, i.e., the distribution of the number of linkers that connect to one hub, will be rather broad and the network topology disordered, in which case the PEG end-to-end distance R_{PEG} will be significantly smaller than the pore size l_0 (cf. also Estimate of Mean Hydrogel Mesh Size). Whereas in a cubic lattice, each cubic facet consists of four hubs and four linkers, the pores present in the actual hydrogel will show a broad distribution of the number of participating linkers. For illustration, the pore shown in Fig. 6 *C* consists of eight linkers. Clearly, dextran molecules will tend to be located in larger pores to maximize their free volume, and therefore, the fit parameters of our model will be dominated by the tail of the pore-size distribution, which explains the large fit values for l_0. This finding also allows us to rationalize the larger extracted free energy in the hydrogel in the case of the *hPG-G6* gel, even though the *hPG-G10* gel mass density is higher (cf. Fig. 5 *C*). The tail of the pore-size distribution of the *hPG-G10* gel presumably contains larger pores that can stretch even further to minimize the unfavorable dextran-PEG interactions. Clearly, the precise topology and compositional distribution of pores cannot be predicted by our analysis; our results should thus be merely interpreted as an indication of the presence of large pores and a disordered network topology.

An approximate nonelastic version of the free-volume model is obtained by neglecting the polymer deformation term and just keeping the excluded volume term, Eq. 9, which becomes accurate in the limit of $l_0 >> r_0$, where $r^* \approx r_0$ and $l^* \approx l_0$. These approximate results are shown as broken lines in Fig. 6 *B* and describe the experimental data only for small values of r_0/l_0. When additionally approximating the logarithm in Eq. 9, the obtained expression for the free energy is similar to results derived for a random-fiber network (50). Our free-volume model is valid only for short-ranged steric and hydration repulsive interactions between diffusor and linkers; if long-ranged and, in particular, attractive interactions are present—for example, electrostatic interactions for low salt concentrations—the model would need to be adjusted accordingly.

Derivation of particle permeabilities through hydrogel barriers

Permeation through biological barriers is quantified by the permeability coefficient P, which is defined as (51)

$$P(z_1, z_2) = \frac{J}{c(z_1) - c(z_2)}, \tag{13}$$

where $c(z_1)$ and $c(z_2)$ are the particle concentrations at the two sides z_1 and z_2 of the barrier and J denotes the particle flux through the barrier. Based on the diffusion equation (Eq. 6), the inverse permeability can be written as (for a detailed derivation, see Supporting Materials and Methods, Section S10)

$$\frac{1}{P(z_1, z_2)} = \int_{z_1}^{z_2} \frac{e^{\beta F(z)}}{D(z)} dz. \tag{14}$$

For a step-like barrier, one obtains

$$\frac{1}{P} = \frac{e^{\beta \Delta F_{gel}}}{D_{gel}} L. \tag{15}$$

Here, ΔF_{gel} and D_{gel} are the particle free energy relative to the solution and the diffusivity inside the hydrogel, and L denotes the width of the hydrogel barrier.

Fig. 7 *A* shows normalized permeability coefficients PL for a single step-like barrier according to Eq. 15, which are independent of the thickness of the barrier L, as a function of the gel free energy and the gel diffusivity. The values extracted from the experimental data for different dextran molecules in the two gels from Fig. 5 are indicated by data points. Obviously, the highest permeability is observed for a low free-energy barrier and a high particle diffusivity, as is the case for the smallest dextran molecules (*lower right corner* in Fig. 7 *A*). On the other hand, permeation is hindered by either a high free-energy barrier or a low diffusivity in the hydrogel, both of which are observed

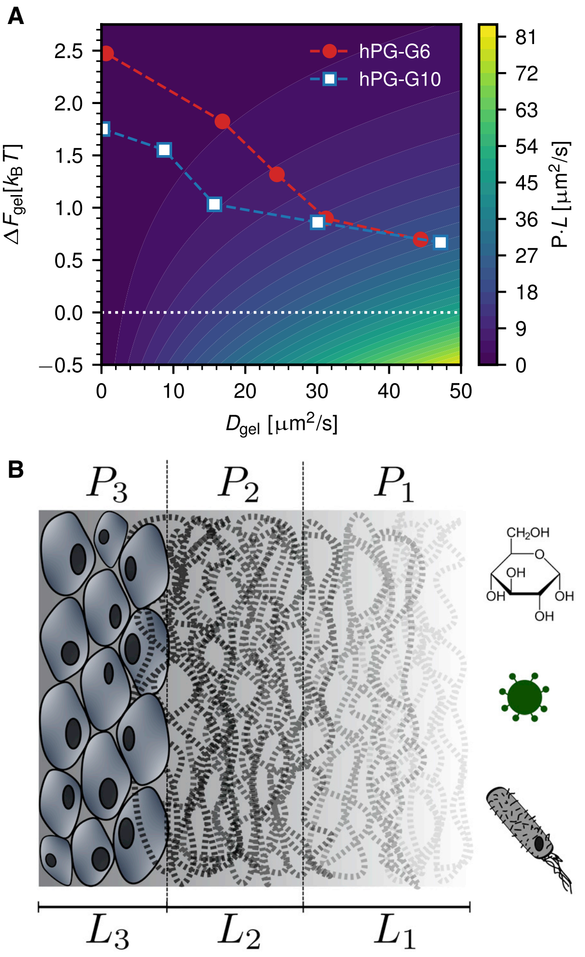

FIGURE 7 (*A*) Normalized permeability coefficient *PL* through a single box-like hydrogel barrier of width *L* as a function of the hydrogel free energy ΔF_{gel} and the hydrogel diffusivity D_{gel} from Eq. 15. High permeability is observed for low free-energy barriers and high diffusivities in the hydrogel. The symbols denote the experimental data from Fig. 5. Because of opposing trends in the free-energy barrier and the diffusivity, both hydrogels display comparable permeability coefficients. (*B*) Schematic layered structure of a mucous membrane, as found in the stomach, is given. Examples for different diffusors are shown, including nutrients such as glucose and pathogens such as virions or bacteria. The diffusors have to penetrate different layers of varying permeabilities to enter the tissue below the mucous membranes, the total permeability of a layered structure follows from Eq. 16. To see this figure in color, go online.

for dextran molecules with larger molecular weights. Because of counterbalancing effects of stronger exclusion from the *hPG-G6* gel and increased immobilization in the case of *hPG-G10*, both hydrogels display comparable permeability coefficients for the chosen dextran molecular masses.

CONCLUSIONS

The method introduced in this work allows for the simultaneous extraction of diffusivity and free-energy profiles of particles that permeate into spatially inhomogeneous hydrogel systems; we demonstrate the method using concentration profile measurements of fluorescently labeled dextran molecules permeating into PEG-hPG-based hydrogels. The advantage over alternative methods is that both diffusivity and free-energy profiles are obtained from a single experimental setup. This is important because only the combination of diffusivity and free-energy profiles completely determines the diffusion of particles.

The extracted diffusivities and free energies are analyzed in terms of empirical scaling laws as a function of the dextran mass, and a modified elastic free-volume model is developed that quantitatively accounts for the particle free energy in the hydrogel. Although the free volume accessible to a diffusor inside a hydrogel has been previously shown to determine diffusion properties in biological systems, such as crowded cellular membranes (52), our modified free-volume model additionally includes the elasticity of linkers and of the diffusing molecules and thereby quantitatively accounts for the free energies we extracted from the experimental data of dextran diffusing in PEG-based hydrogels. This demonstrates that elastic deformations of both the diffusor and the hydrogel network are important, in line with previous computational (53–55) and experimental studies (56). Our model furthermore unveils significant topological disorder of the hydrogel pores and suggests that the dextran molecules preferentially partition into exceptionally large pores, which are locally even more enlarged because of PEG strand elasticity.

Diffusional barriers in biological systems often show a layered structure, as previously demonstrated for skin (29–31) and also known to be true for mucous membranes, which are found, for instance, in the gastrointestinal tract, schematically indicated in Fig. 7 *B*. For a layered system, Eq. 14 shows that the individual piecewise constant permeability coefficients P_i add up inversely as

$$\frac{1}{P_{tot}} = \sum_i \frac{1}{P_i} = \sum_i \frac{e^{\beta \Delta F_i}}{D_i} L_i = \sum_i \frac{L_i}{D_i K_i}, \qquad (16)$$

where the sum goes over all layers, represented by their respective diffusion constants D_i, free-energy values ΔF_i or partition coefficients K_i, and thicknesses L_i. Here, P_{tot} denotes the total permeability, which is dominated by the smallest permeability in the inverse sum.

Fig. 7 *B* schematically illustrates permeation through a layered system which represents the mammalian stomach (57). The outermost layer of mucus is only loosely bound and characterized by the permeability P_1; it is followed by a layer of more tightly bound mucus, characterized by P_2, and adheres onto the first layer of epithelial cells, characterized by P_3. The total thickness of this diffusional barrier is about a millimeter, with the two mucus layers spanning a few hundred micrometers only (58). Measurements in rat gastrointestinal mucosa suggest typical values

of $L_1 = 109$ μm, $L_2 = 80$ μm, and $L_3 \approx L_2$ (59), which are close to the range of gel thicknesses studied in this work.

The total permeability is determined by the free energies and the mobilities inside all layers. Nutrients, for instance, can easily penetrate through the epithelia of the gastrointestinal tract, displaying large permeabilities in the different layers. Pathogens, on the other hand, are in healthy environments kept from reaching the epithelium because of low permeability in the tightly bound mucus layer ($P_2 << P_1$) (57). From Eq. 16, it is apparent that the lowest permeability in such a layered system dominates the total permeability, leading to an effective barrier function that for different particles can be caused by different parts of the layered barrier structure.

The method introduced in this work determines free-energy and diffusivity profiles from experimental data and thereby can be used to predict effective permeabilities of different kinds of molecules, particles, or even organisms into various layered systems, including systems that contain hydrogels and mucus. A multilayered structure, as shown in Fig. 7 *B*, can be produced by cultivating mucous-producing cells in vitro and can be studied using the framework introduced in this work. This would allow the detailed analysis of permeabilities of different diffusors through an in vitro representation of an actual biological barrier. We believe that the technical advances described in this work will help to shed light on the underlying mechanisms of the function of general biological barriers including mucous membranes.

SUPPORTING MATERIAL

Supporting Material can be found online at https://doi.org/10.1016/j.bpj.2020.12.020.

AUTHOR CONTRIBUTIONS

A.H., S.B., and R.H. designed and performed the CLSM experiments for the determination of the dextran concentration profiles. A.P. and M.G. designed and performed the FCS experiments. A.W.-K. and R.R.N. designed the models, analyzed the experimental data, and wrote the manuscript.

ACKNOWLEDGMENTS

The authors acknowledge funding by the Deutsche Forschungsgemeinschaft via grant SFB 1449.

SUPPORTING CITATIONS

References (60–68) appear in the Supporting Material.

REFERENCES

1. Wirthl, D., R. Pichler, ..., M. Kaltenbrunner. 2017. Instant tough bonding of hydrogels for soft machines and electronics. *Sci. Adv.* 3:e1700053.
2. Herrmann, A., L. Kaufmann, ..., U. Schedler. 2018. Bioorthogonal in situ hydrogels based on polyether polyols for new biosensor materials with high sensitivity. *ACS Appl. Mater. Interfaces.* 10:11382–11390.
3. Li, J., and D. J. Mooney. 2016. Designing hydrogels for controlled drug delivery. *Nat. Rev. Mater.* 1:16071.
4. Billings, N., A. Birjiniuk, ..., K. Ribbeck. 2015. Material properties of biofilms-a review of methods for understanding permeability and mechanics. *Rep. Prog. Phys.* 78:036601.
5. Rosales, A. M., and K. S. Anseth. 2016. The design of reversible hydrogels to capture extracellular matrix dynamics. *Nat. Rev. Mater.* 1:15012.
6. Lieleg, O., I. Vladescu, and K. Ribbeck. 2010. Characterization of particle translocation through mucin hydrogels. *Biophys. J.* 98:1782–1789.
7. Wagner, C. E., K. M. Wheeler, and K. Ribbeck. 2018. Mucins and their role in shaping the functions of mucus barriers. *Annu. Rev. Cell Dev. Biol.* 34:189–215.
8. Hollingsworth, M. A., and B. J. Swanson. 2004. Mucins in cancer: protection and control of the cell surface. *Nat. Rev. Cancer.* 4:45–60.
9. McGuckin, M. A., S. K. Lindén, ..., T. H. Florin. 2011. Mucin dynamics and enteric pathogens. *Nat. Rev. Microbiol.* 9:265–278.
10. Dawson, M., D. Wirtz, and J. Hanes. 2003. Enhanced viscoelasticity of human cystic fibrotic sputum correlates with increasing microheterogeneity in particle transport. *J. Biol. Chem.* 278:50393–50401.
11. Lai, S. K., D. E. O'Hanlon, ..., J. Hanes. 2007. Rapid transport of large polymeric nanoparticles in fresh undiluted human mucus. *Proc. Natl. Acad. Sci. USA.* 104:1482–1487.
12. Johansson, M. E. V. 2012. Fast renewal of the distal colonic mucus layers by the surface goblet cells as measured by in vivo labeling of mucin glycoproteins. *PLoS One.* 7:e41009.
13. Button, B., L.-H. Cai, ..., M. Rubinstein. 2012. A periciliary brush promotes the lung health by separating the mucus layer from airway epithelia. *Science.* 337:937–941.
14. Witten, J., and K. Ribbeck. 2017. The particle in the spider's web: transport through biological hydrogels. *Nanoscale.* 9:8080–8095.
15. Li, L. D., T. Crouzier, ..., K. Ribbeck. 2013. Spatial configuration and composition of charge modulates transport into a mucin hydrogel barrier. *Biophys. J.* 105:1357–1365.
16. Marczynski, M., B. T. Käsdorf, ..., O. Lieleg. 2018. Transient binding promotes molecule penetration into mucin hydrogels by enhancing molecular partitioning. *Biomater. Sci.* 6:3373–3387.
17. Zhang, X., J. Hansing, ..., J. E. DeRouchey. 2015. Particle transport through hydrogels is charge asymmetric. *Biophys. J.* 108:530–539.
18. Hansing, J., C. Ciemer, ..., R. R. Netz. 2016. Nanoparticle filtering in charged hydrogels: effects of particle size, charge asymmetry and salt concentration. *Eur Phys J E Soft Matter.* 39:53.
19. Hansing, J., and R. R. Netz. 2018. Particle trapping mechanisms are different in spatially ordered and disordered interacting gels. *Biophys. J.* 114:2653–2664.
20. Hansing, J., J. R. Duke, III, ..., R. R. Netz. 2018. Particle diffusion in polymeric hydrogels with mixed attractive and repulsive interactions. *Nano Lett.* 18:5248–5256.
21. Dawson, M., E. Krauland, ..., J. Hanes. 2004. Transport of polymeric nanoparticle gene carriers in gastric mucus. *Biotechnol. Prog.* 20:851–857.
22. Wagner, C. E., B. S. Turner, ..., K. Ribbeck. 2017. A rheological study of the association and dynamics of MUC5AC gels. *Biomacromolecules.* 18:3654–3664.
23. Wilking, J. N., V. Zaburdaev, ..., D. A. Weitz. 2013. Liquid transport facilitated by channels in Bacillus subtilis biofilms. *Proc. Natl. Acad. Sci. USA.* 110:848–852.
24. Lawrence, J. R., G. M. Wolfaardt, and D. R. Korber. 1994. Determination of diffusion coefficients in biofilms by confocal laser microscopy. *Appl. Environ. Microbiol.* 60:1166–1173.

25. Cherstvy, A. G., S. Thapa, …, R. Metzler. 2019. Non-Gaussian, non-ergodic, and non-Fickian diffusion of tracers in mucin hydrogels. *Soft Matter.* 15:2526–2551.

26. Sassi, A. P., A. J. Shaw, …, J. M. Prausnitz. 1996. Partitioning of proteins and small biomolecules in temperature- and pH-sensitive hydrogels. *Polymer (Guildf.).* 37:2151–2164.

27. Herrmann, A., S. Rödiger, …, U. Schedler. 2019. Spatial separation of microbeads into detection levels by a bioorthogonal porous hydrogel for size-selective analysis and increased multiplexity. *Anal. Chem.* 91:8484–8491.

28. Furter, M., M. E. Sellin, …, W.-D. Hardt. 2019. Mucus architecture and near-surface swimming affect distinct Salmonella typhimurium infection patterns along the murine intestinal tract. *Cell Rep.* 27:2665–2678.e3.

29. Schulz, R., K. Yamamoto, …, R. R. Netz. 2017. Data-based modeling of drug penetration relates human skin barrier function to the interplay of diffusivity and free-energy profiles. *Proc. Natl. Acad. Sci. USA.* 114:3631–3636.

30. Schulz, R., K. Yamamoto, …, R. R. Netz. 2019. Modeling of drug diffusion based on concentration profiles in healthy and damaged human skin. *Biophys. J.* 117:998–1008.

31. Lohan, S. B., S. Saeidpour, …, M. C. Meinke. 2020. Nanocrystals for improved drug delivery of dexamethasone in skin investigated by EPR spectroscopy. *Pharmaceutics.* 12:400.

32. Dey, P., T. Schneider, …, R. Haag. 2016. Mimicking of chondrocyte microenvironment using in situ forming dendritic polyglycerol sulfate-based synthetic polyanionic hydrogels. *Macromol. Biosci.* 16:580–590.

33. Branch, M. A., T. F. Coleman, and Y. Li. 1999. A subspace, interior, and conjugate gradient method for large-scale bound-constrained minimization problems. *SIAM J. Sci. Comput.* 21:1–23.

34. Gribbon, P., and T. E. Hardingham. 1998. Macromolecular diffusion of biological polymers measured by confocal fluorescence recovery after photobleaching. *Biophys. J.* 75:1032–1039.

35. Risken, H., and T. Frank. 2012. The Fokker-Planck Equation: Methods of Solution and Applications. Springer Series in Synergetics: Springer, Berlin, Germany.

36. Yamamoto, E., T. Akimoto, …, R. Metzler. 2020. Universal relation between instantaneous diffusivity and radius of gyration of proteins in aqueous solution 1–15. *arXiv*, arXiv:2009.06829 http://arxiv.org/abs/2009.06829.

37. Cheng, Y., R. K. Prud'homme, and J. L. Thomas. 2002. Diffusion of mesoscopic probes in aqueous polymer solutions measured by fluorescence recovery after photobleaching. *Macromolecules.* 35:8111–8121.

38. Bu, Z., and P. S. Russo. 1994. Diffusion of dextran in aqueous (Hydroxypropyl)cellulose. *Macromolecules.* 27:1187–1194.

39. Rubinstein, M., and R. H. Colby. 2003. Polymer Physics. Oxford University Press, Oxford, UK.

40. Netz, R. R., and D. Andelman. 2003. Neutral and charged polymers at interfaces. *Phys. Rep.* 380:1–95.

41. Granath, K. A. 1958. Solution properties of branched dextrans. *J. Colloid Sci.* 13:308–328.

42. Hansing, J., and R. R. Netz. 2018. Hydrodynamic effects on particle diffusion in polymeric hydrogels with steric and electrostatic particle–gel interactions. *Macromolecules.* 51:7608–7620.

43. de Gennes, P. G. 1971. Reptation of a polymer chain in the presence of fixed obstacles. *J. Chem. Phys.* 55:572–579.

44. Axpe, E., D. Chan, …, E. A. Appel. 2019. A multiscale model for solute diffusion in hydrogels. *Macromolecules.* 52:6889–6897.

45. Wang, Y.-Y., S. K. Lai, …, J. Hanes. 2008. Addressing the PEG mucoadhesivity paradox to engineer nanoparticles that "slip" through the human mucus barrier. *Angew. Chem. Int.Engl.* 47:9726–9729.

46. Kanduč, M., and R. R. Netz. 2015. From hydration repulsion to dry adhesion between asymmetric hydrophilic and hydrophobic surfaces. *Proc. Natl. Acad. Sci. USA.* 112:12338–12343.

47. Schneck, E., F. Sedlmeier, and R. R. Netz. 2012. Hydration repulsion between biomembranes results from an interplay of dehydration and depolarization. *Proc. Natl. Acad. Sci. USA.* 109:14405–14409.

48. Moore, M. 1974. Symmetrical intersections of right circular cylinders. *Math. Gaz.* 58:181.

49. Liese, S., M. Gensler, …, R. R. Netz. 2017. Hydration effects turn a highly stretched polymer from an entropic into an energetic spring. *ACS Nano.* 11:702–712.

50. Giddings, J. C., E. Kucera, …, M. N. Myers. 1968. Statistical theory for the equilibrium distribution of rigid molecules in inert porous networks. Exclusion chromatography. *J. Phys. Chem.* 72:4397–4408.

51. Diamond, J. M., and Y. Katz. 1974. Interpretation of nonelectrolyte partition coefficients between dimyristoyl lecithin and water. *J. Membr. Biol.* 17:121–154.

52. Jeon, J.-H., M. Javanainen, …, I. Vattulainen. 2016. Protein crowding in lipid bilayers gives rise to non-Gaussian anomalous lateral diffusion of phospholipids and proteins. *Phys. Rev. X.* 6:021006.

53. Zhou, H., and S. B. Chen. 2009. Brownian dynamics simulation of tracer diffusion in a cross-linked network. *Phys. Rev. E Stat. Nonlin. Soft Matter Phys.* 79:021801.

54. Godec, A., M. Bauer, and R. Metzler. 2014. Collective dynamics effect transient subdiffusion of inert tracers in flexible gel networks. *New J. Phys.* 16:092002.

55. Kumar, P., L. Theeyancheri, …, R. Chakrabarti. 2019. Transport of probe particles in a polymer network: effects of probe size, network rigidity and probe-polymer interaction. *Soft Matter.* 15:8992–9002.

56. Mohamed, H. F. M., Y. Kobayashi, …, A. Ohira. 2009. Effects of ion exchange on the free volume and oxygen permeation in Nafion for fuel cells. *J. Phys. Chem. B.* 113:2247–2252.

57. Johansson, M. E. V., H. Sjövall, and G. C. Hansson. 2013. The gastrointestinal mucus system in health and disease. *Nat. Rev. Gastroenterol. Hepatol.* 10:352–361.

58. Jordan, N., J. Newton, …, A. Allen. 1998. A novel method for the visualization of the in situ mucus layer in rat and man. *Clin. Sci. (Lond.).* 95:97–106.

59. Atuma, C., V. Strugala, …, L. Holm. 2001. The adherent gastrointestinal mucus gel layer: thickness and physical state in vivo. *Am. J. Physiol. Gastrointest. Liver Physiol.* 280:G922–G929.

60. Widengren, J., R. Rigler, and U. Mets. 1994. Triplet-state monitoring by fluorescence correlation spectroscopy. *J. Fluoresc.* 4:255–258.

61. Rigler, R., and E. S. Elson. 2012. Fluorescence Correlation Spectroscopy: Theory and Applications. Springer Series in Chemical Physics: Springer, Berlin, Germany.

62. Rüttinger, S., V. Buschmann, …, F. Koberling. 2008. Comparison and accuracy of methods to determine the confocal volume for quantitative fluorescence correlation spectroscopy. *J. Microsc.* 232:343–352.

63. Huang, L., and K. Nishinari. 2001. Interaction between poly(ethylene glycol) and water as studied by differential scanning calorimetry. *J. Polym. Sci., B, Polym. Phys.* 39:496–506.

64. Shikata, T., R. Takahashi, and A. Sakamoto. 2006. Hydration of poly(ethylene oxide)s in aqueous solution as studied by dielectric relaxation measurements. *J. Phys. Chem. B.* 110:8941–8945.

65. Kaatze, U., O. Gottmann, …, U. Terveer. 1978. Dielectric relaxation in aqueous solutions of some oxygen-containing linear hydrocarbon polymers. *J. Phys. Chem.* 82:112–120.

66. Bieze, T. W. N., A. C. Barnes, …, J. C. Leyte. 1994. Distribution of water around poly(ethylene oxide): a neutron diffraction study. *J. Phys. Chem.* 98:6568–6576.

67. Żwirbla, W., A. Sikorska, and B. B. Linde. 2005. Ultrasonic investigations of water mixtures with polyethylene glycols 200, 400 and ethylene glycol. *J. Mol. Struct.* 743:49–52.

68. Branca, C., S. Magazù, …, G. Romeo. 2002. Hydration study of PEG/water mixtures by quasi elastic light scattering, acoustic and rheological measurements. *J. Phys. Chem. B.* 106:10272–10276.

Biophysical Journal, Volume *120*

Supplemental Information

Particle Diffusivity and Free-Energy Profiles in Hydrogels from Time-Resolved Penetration Data

Amanuel Wolde-Kidan, Anna Herrmann, Albert Prause, Michael Gradzielski, Rainer Haag, Stephan Block, and Roland R. Netz

S1 Molecular Mass Distributions of PEG linkers and Dextran Molecules

The dextran molecular mass distribution is characterized using gel permeation chromatography (GPC), the results of which are presented in Table S1. GPC measurements are performed on an *Agilent* device (1100er series) with a PSS Suprema column (pre-column, 1x with poresize of 30 Å, 2x with poresize of 1000 Å, all of them with a particle size of 10 μm), with pullulan as calibration standard, and ethylene glycol as internal standard. As solvent, H_2O with 0.1 M $NaNO_3$ is used. For the characterization of the PEG linker mass distribution matrix assisted laser desorption ionization (MALDI) on a *Bruker Ultraflex* II is performed. The obtained values are shown in Table S2. From the number average molecular mass M_n and the weight average molecular mass M_w the polydispersity index PDI is determined as

$$PDI = \frac{M_\mathrm{w}}{M_\mathrm{n}}. \tag{S1}$$

The dextran molecules show a considerable dispersion regarding the molecular mass, while the PEG linker mass distribution is rather uniform.

Table S1: Results obtained from GPC measurements of the different dextran molecules.

M_dex [kDa]	M_w [kDa]	M_n [kDa]	PDI
4	3.55	2.32	1.53
10	9.55	5.55	1.72
20	16.5	9.42	1.75
40	35.7	19.5	1.84
70	61.9	50.1	1.24

Table S2: Molecular weights and polydispersity of the PEG linkers as measured in MALDI experiments.

M_PEG [kDa]	M_w [kDa]	M_n [kDa]	PDI
6	6.29	5.90	1.07
10	11.3	11.3	1.00

S2 Hydrogel Volume Reconstruction

The hydrogel volume is determined using confocal laser scanning microscopy as follows. The hydrogels are first equilibrated using PBS buffer, followed by injection of the $M_{\mathrm{dex}} = 70$ kDa FITC-labeled dextran and recording of 3D dextran concentration profiles using confocal microscopy (covering an imaged volume of 2,304 x 2,304 x 0.5 mm^3). The permeation measurements (shown in the main text) reveal that this dextran is too large to penetrate the hydrogel, so that the hydrogel can be identified in these 3D concentration profiles based on an exclusion of FITC-labeled dextran (i.e., absence of FITC fluorescence; see Figure S1). In order to quantify the hydrogel volume, the (2D) hyperplane at which the FITC intensity has dropped to 50% of its bulk value is determined using home-written scripts in *Matlab* (*MathWorks*, Natick, MA). This hyperplane indicates the positions, at which the point spread function of the confocal microscope is equally filled by the FITC-dextran bulk solution and either the hydrogel or the supporting substrate, and therefore allows for extraction of the exact locations of the substrate and hydrogel interface within the sample. Furthermore, as the substrate is a flat glass slide, the hydrogel-substrate interface is extracted from this data by first fitting a plane to the regions corresponding to the substrate-bulk interface (i.e., in regions far away from the hydrogel spot) and by interpolating the position of this plane underneath the hydrogel. This procedure allows for extraction of the entire hydrogel boundary, allowing determination of the hydrogel volume by numerical integration. This yields volumes of $V_{\mathrm{tot}}^{\mathrm{hPG\text{-}G6}} = 0.42 \pm 0.03$ μL and $V_{\mathrm{tot}}^{\mathrm{hPG\text{-}G10}} = 0.31 \pm 0.04$ μL for the two hydrogels.

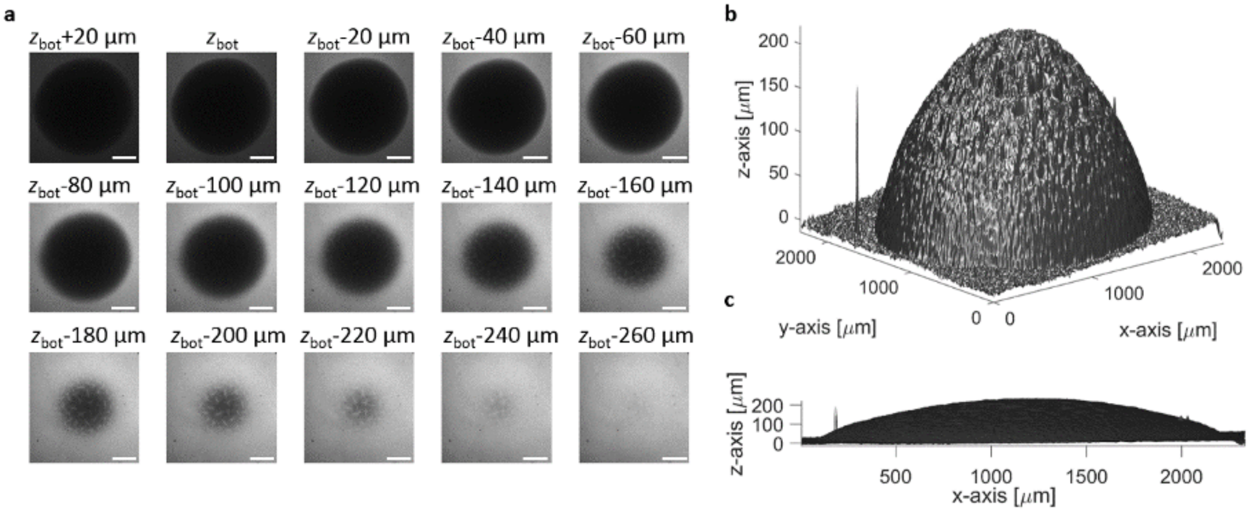

Supplementary Figure S1: In these experiments, the hydrogels have been equilibrated using PBS buffer (as described in Methods Section of the main text) followed by application of a $M_{\mathrm{dex}} = 70$ kDa dextran solution. The dextran is too large to penetrate into the hydrogel as evidenced by using confocal fluorescence microscopy to record multiple sample sections (at different z-heights as indicated in (a)). In these images, the hydrogel appears as a black circle, the radius of which decreases with increasing distance to the glass interface (located at the z-height z_{bot}). The bright areas correspond to FITC-dextran solution in the bulk. These images can be used to extract the position of the glass interface and hydrogel in space. A 3D representation and a side view of this hyperplane are given in (b) and (c), respectively, showing that the hydrogels possess a semi-elliptical shape with radii being on the order of 1050 μm (major axis) and 150 – 210 μm (minor axis). This figure shows a representative measurement for the *hPG-G6* gel. The scale bars in (a) correspond to 500 μm.

S3 Fitting Procedure for FCS Measurements

An accurate description of the dextran autocorrelation functions (ACFs) G(τ) required to use at least 2 components, which originate from the fluorescence emission of the FITC-labeled dextrans and, in addition, from residuals of free FITC molecules (i.e., not being conjugated to dextran). The FCS ACF G(τ) was therefore described using the equation

$$G(\tau) = T(\tau) \cdot \left(G_{\mathrm{F}}(\tau) + G_{\mathrm{DF}}(\tau)\right), \tag{S2}$$

in which $G_{\mathrm{F}}(\tau)$ and $G_{\mathrm{DF}}(\tau)$ give the contributions from free FITC molecules and FITC-labeled dextrans, respectively, while the term $T(\tau)$ accounts for triplet state dynamics according to

$$T(\tau) = \frac{\left(1 - \Phi_{\mathrm{T}} + \Phi_{\mathrm{T}} e^{-\frac{\tau}{\tau_{\mathrm{T}}}}\right)}{1 - \Phi_{\mathrm{T}}}, \tag{S3}$$

with Φ_{T} denoting the fraction of molecules in the triplet state and τ_{T} the corresponding decay time of triplet states.[1]

The contribution of the free FITC molecules was modeled using the theoretically derived ACF for free diffusion in 3D

$$G_{\mathrm{F}}(\tau) = \rho_{\mathrm{F}} \left(1 + \frac{\tau}{\tau_{\mathrm{F}}}\right)^{-1} \left(1 + \frac{\tau}{\tau_{\mathrm{F}} \kappa^2}\right)^{-\frac{1}{2}}, \tag{S4}$$

in which ρ_{F} denotes the average number density of free FITC molecules in the confocal readout volume, τ the lag time of the ACF, κ the ratio of axial r_{z} to radial extension r_{xy} of the confocal readout volume, and τ_{F} the decay time.[2] The value of τ_{F} was determined from calibration measurements on FITC molecules diffusing in buffer and κ was fixed to 6,[3] so that only the density ρ_{F} was a free parameter when fitting the component $G_{\mathrm{F}}(\tau)$ to experimentally determined ACF.

Since the dextrans showed a log-normal size distribution, as observed in the GPC mea-

surements, the component of FITC-labeled dextrans of the ACF, $G_{\mathrm{DF}}(\tau)$, was modeled by the superposition

$$G_{\mathrm{DF}}(\tau) = \sum_i p_{\mathrm{i}} G_{\mathrm{DF,i}}(\tau), \tag{S5}$$

using the log-normally distributed weights

$$p_{\mathrm{i}} := \frac{1}{\tau_{\mathrm{DF,i}} \sigma_{\mathrm{DF}} \sqrt{2\pi}} e^{-\frac{\left(\ln(\tau_{\mathrm{DF,i}}) - \mu_{\mathrm{DF}}\right)^2}{2\sigma_{\mathrm{DF}}^2}}, \tag{S6}$$

and the corresponding ACF contributions

$$G_{\mathrm{DF,i}}(\tau) := \rho_{\mathrm{DF}} \left(1 + \frac{\tau}{\tau_{\mathrm{DF,i}}}\right)^{-1} \left(1 + \frac{\tau}{\tau_{\mathrm{DF,i}} \kappa^2}\right)^{-\frac{1}{2}}. \tag{S7}$$

The parameter σ_{DF}, which determines the broadness of the log-normal distribution, was determined by matching the FCS-related polydispersity index (PDI), defined by

$$PDI_{\mathrm{FCS}} = \frac{\sum_i p_i M_i^2}{\left(\sum_i p_i M_i\right)^2}, \tag{S8}$$

using

$$M_i \propto R_i^3 \tag{S9a}$$

$$R_i = \frac{k_{\mathrm{B}} T}{6\pi\eta D_i} \tag{S9b}$$

$$D_i = \frac{r_{\mathrm{xy}}^2}{4\tau_{\mathrm{DF,i}}}, \tag{S9c}$$

where the PDI of the dextran mass distribution was determined using GPC. The parameters ρ_{DF} and $\tau_{\mathrm{DF}} = e^{\mu_{\mathrm{DF}}}$ denote the average number density of FITC-labeled dextran molecules in the confocal readout volume and their average decay time, respectively, and were determined when fitting the component $G_{\mathrm{DF}}(\tau)$ to the experimentally determined ACF.

Fitting the 2-component model therefore yields information about the number densities of free FITC molecules and FITC-labeled dextran molecules in the confocal readout volume (ρ_{F} and ρ_{DF}, respectively) and their decay times τ_{F} and τ_{DF}, which can be translated into diffusion coefficients using

$$D_{\mathrm{F}} = \frac{r_{\mathrm{xy}}^2}{4\tau_{\mathrm{F}}} \tag{S10a}$$

$$D_{\mathrm{DF}} = \frac{r_{\mathrm{xy}}^2}{4\tau_{\mathrm{DF}}}, \tag{S10b}$$

and into hydrodynamic radii using the Stokes-Einstein relation.[2]

S4 Measured Fluorescence Intensity Data

The experimentally measured fluorescence intensity data displays a continuous drift in the signal in all recorded measurements, which is likely due to an automatic re-adjustment of the laser intensity in the used setup. An example of the observed drift in the raw unscaled signal is shown in Figure S2A, recorded for $M_{\mathrm{dex}} = 70$ kDa dextran molecules at the *hPG-G10* interface. Even though almost no penetration of the large dextran molecules into the hydrogel is observed, the fluorescence intensity in the probed part of the bulk solution changes significantly over time. In order to obtain physical values for the dextran concentration, the measured profiles are being re-scaled during the fitting procedure. The obtained re-scaling factors for every measured concentration profile $\vec{f}$ decline over time, thus overcoming the constant increase of signal intensity due to the drift (see Figure S3A). Additionally, smaller changes in the fluorescence intensity are apparent. Since robust results are obtained by employing this re-scaling routine, this suggests that the entire information about the diffusion process is present in the relative shape of the concentration profiles.

The obtained re-scaling factor can additionally be used, to estimate the experimental bulk concentration c_{bulk} far away from the hydrogel interface, based on the experimentally measured profiles alone, without using the numerically computed dextran distributions. The total amount of dextran in the system C_{tot} is computed from the first concentration profile as $C_{\mathrm{tot}} = \int_{-\infty}^{\infty} c(z, t = 0)\, dz$, where $c(z, t = 0)$ was approximated by c_i^{init} according to eq. (4) in the main text. An average experimental concentration in the bulk region $\bar{c}_{\mathrm{bulk}}(t_{\mathrm{j}})$ can then be estimated from the fitted re-scaling factors as

$$\bar{c}_{\mathrm{bulk}}(t_{\mathrm{j}}) = \frac{C_{\mathrm{tot}} - \sum_{i=1}^{M} f_{\mathrm{j}} \cdot c_i^{\mathrm{exp}}(t_j) \cdot \Delta z_i}{z_{\mathrm{top}}}. \tag{S11}$$

Values for $\bar{c}_{\mathrm{bulk}}(t_{\mathrm{j}})$ are shown in Figure S3B and are virtually constant for the exemplary measurement of $M_{\mathrm{dex}} = 70$ kDa dextrans, as is expected due to the absence of penetration into the hydrogel.

The re-scaling procedure is additionally validated by a comparison of the concentration profiles obtained for the M_{dex} = 40 kDa dextran molecules, diffusing into the *hPG-G10* hydrogel, which is presented in Fig. S2B. The concentration profiles were measured in two different experiments, with a period of one month in between. The observed good agreement between the data sets, with significant deviations being only present in the transition region between the hydrogel and the bulk solution, corroborates the re-scaling method used to obtain concentration profiles from the fluorescence intensity data, as well as the experimental setup.

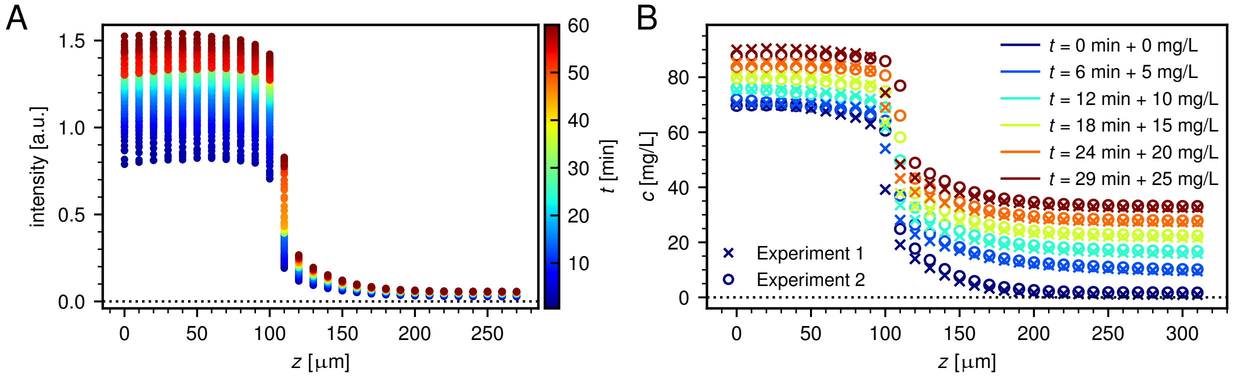

Supplementary Figure S2: A: Raw fluorescence intensity data from experiments of M_{dex} = 70 kDa dextrans in combination with the *hPG-G10* hydrogel. A significant change in the signal over time is observed in the probed part of the bulk solution, even though almost no penetration of the dextrans into the hydrogel is apparent. This drift in the experimentally measured signal is overcome by the numerically determined re-scaling factors. B: Re-scaled concentration profiles measured one month apart in two different experiments for the M_{dex} = 40 kDa dextran molecules, penetrating into the *hPG-G10* gel. Good reproducibility is observed in the data. Note that profiles at different timepoints are shifted for an easier comparison.

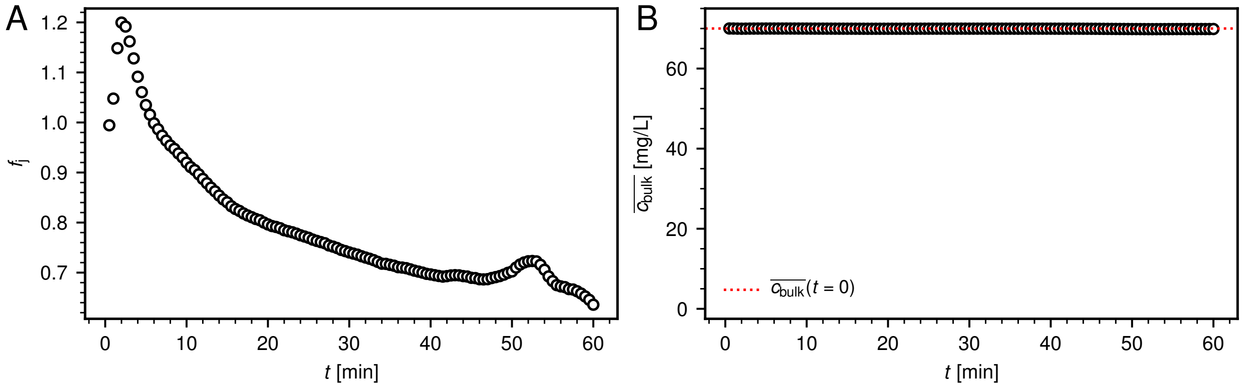

Supplementary Figure S3: A: Set of re-scaling factors $\vec{f}$ for every measured concentration profile obtained from numerical analysis of experimental data from $M_{\mathrm{dex}} = 70$ kDa dextrans in contact with the *hPG-G10* hydrogel. Decreasing re-scaling factors counteract the drift observed in the raw experimental data. Additionally, peaks in the re-scaling factor distribution are observed, counteracting shorter fluctuations in the fluorescence intensity. B: Average bulk concentration $\overline{c_{\mathrm{bulk}}}$ computed according to eq. (S11) for the same measurements. The bulk concentration remains constant in this measurement, since almost no dextran penetrates into the hydrogel.

S5 Analytical Solution for Two-Segment System

Simplifying the hydrogel-water setup as a two-box system with piece-wise constant values of the free-energy and diffusion constant in the two regions allows for an analytical solution of the diffusion problem. The modeled system with the corresponding boundary conditions is sketched in Figure S4A.

We solve the following diffusion equation in each of the two segments

$$\frac{\partial}{\partial t}c(z,t) = D(z)\frac{\partial^2}{\partial z^2}c(z,t), \tag{S12}$$

where the diffusion constant $D(z)$ has a different value in each of the two regions

$$D(z) = \begin{cases} D_0, & \text{if } 0 \leq z \leq z_{\text{int}} \\ D_1, & \text{if } z_{\text{int}} < z \leq z_{\text{bot}} \end{cases} \tag{S13}$$

as does the free-energy $F(z)$, which we set to zero in the left segment as reference

$$F(z) = \begin{cases} F_0 = 0, & \text{if } 0 \leq z \leq z_{\text{int}} \\ F_1, & \text{if } z_{\text{int}} < z \leq z_{\text{bot}}. \end{cases} \tag{S14}$$

At the interface z_{int}, the flux needs to be continuous due to mass conservation, while the jump in the free-energy leads to a jump in the concentration profile $c(z = z_{\text{int}}, t)$. This defines the boundary conditions at z_{int} as

$$\lim_{z \nearrow z_{\text{int}}} D_0 \frac{\partial}{\partial z}c(z,t) = \lim_{z \searrow z_{\text{int}}} D_1 \frac{\partial}{\partial z}c(z,t) \tag{S15a}$$

$$\lim_{z \nearrow z_{\text{int}}} c(z,t)e^{-\beta F_1} = \lim_{z \searrow z_{\text{int}}} c(z,t), \tag{S15b}$$

Since we are modeling a closed system, the edges at $z = 0$ and $z = z_{\text{bot}}$ are reflecting

boundaries with

$$\frac{\partial}{\partial z}c(z=0,t)=0 \tag{S16a}$$
$$\frac{\partial}{\partial z}c(z=z_{\text{bot}},t)=0. \tag{S16b}$$

Initially, the diffusors are only present in the left segment, modeling the bulk solution. This defines our initial condition as

$$c(z,t=0)=\begin{cases} c_0, & \text{if } 0\le z\le z_{\text{int}} \\ 0, & \text{if } z_{\text{int}}<z\le z_{\text{bot}}. \end{cases} \tag{S17}$$

We now solve eq. (S12) by means of Laplace transformation. To this end, we use the single sided Laplace transform in time, defined as $\hat{f}(s) := \int_0^\infty f(t)e^{-st}dt$, where s is the complex variable in Laplace space $s=\sigma+i\omega$. This converts the partial differential equation (S12) into an ordinary differential equation of second order

$$\left[s-D(z)\frac{\partial^2}{\partial z^2}\right]\hat{c}(z,s)=c(z,t=0). \tag{S18}$$

The general solution of eq. (S18) for the two regions reads

$$\hat{c}(z,s)=\begin{cases} a_1e^{\lambda_0 z}+a_2e^{-\lambda_0 z}+\hat{c}_{\text{p}}, & \text{if } 0\le z\le z_{\text{int}} \\ a_3e^{\lambda_1 z}+a_4e^{-\lambda_1 z}, & \text{if } z_{\text{int}}<z\le z_{\text{bot}} \end{cases} \tag{S19}$$

where we define $\lambda_{\text{i}} := \sqrt{\frac{s}{D_{\text{i}}}}, i=0,1$ and $\hat{c}_{\text{p}} := \frac{c_0}{s}$. The coefficients a_i of eq. (S19) are determined by solving the system of linear equations obtained by Laplace transforming the boundary conditions of eq. (S15) and eq. (S16) and substituting the general solution (eq. (S19)). After some algebra, the solution to the posed problem is obtained as

$$\hat{c}(z,s) = \begin{cases} \hat{c}_\mathrm{p} \frac{K \cdot \tanh(\lambda_1 (z_\mathrm{bot} - z_\mathrm{int}))[\cosh(\lambda_0 z_\mathrm{int}) - \cosh(\lambda_0 z)] + \sinh(\lambda_0 z_\mathrm{int})\sqrt{\delta}}{K \cdot \tanh(\lambda_1 (z_\mathrm{bot} - z_\mathrm{int}))\cosh(\lambda_0 z_\mathrm{int}) + \sinh(\lambda_0 z_\mathrm{int})\sqrt{\delta}}, & \text{if } 0 \leq z \leq z_\mathrm{int} \\ \hat{c}_\mathrm{p} \frac{K \cdot \cosh(\lambda_1 (z_\mathrm{bot} - z))\tanh(\lambda_0 z_\mathrm{int})\sqrt{\delta}}{K \cdot \sinh(\lambda_1 (z_\mathrm{bot} - z_\mathrm{int})) + \tanh(\lambda_0 z_\mathrm{int})\cosh(\lambda_1 (z_\mathrm{bot} - z_\mathrm{int}))\sqrt{\delta}}, & \text{if } z_\mathrm{int} < z \leq z_\mathrm{bot} \end{cases} \tag{S20}$$

where $\delta := \frac{D_0}{D_1}$ and $K := e^{-\beta F_1}$.

The solution in Laplace space (eq. (S20)) is then transformed into real space by use of the Mellin integral

$$\begin{aligned} c(z,t) &= \frac{1}{2\pi i} \int_{s=\sigma - i\infty}^{s=\sigma + i\infty} \hat{c}(z,s) e^{st} \, ds \\ &= \frac{e^{\sigma t}}{2\pi} \int_{-\infty}^{+\infty} \hat{c}(z, \sigma + i\omega) e^{i\omega t} \, dw, \end{aligned} \tag{S21}$$

where the last integral was solved numerically through the inverse discrete Fourier transform.

Figure S4B shows a comparison of the analytical solution and the numerical model for an exemplary parameter set of $z_\mathrm{int} = 100$ μm, $z_\mathrm{bot} = 300$ μm, $c_0 = 1$ mg/L, $D_0 = 50$ $\mu\mathrm{m}^2/s$, $D_1 = 100$ $\mu\mathrm{m}^2/s$ and $F_1 = 0.5$ $k_\mathrm{B}T$, mimicking a slight immobilization and repulsion in the right segment, as observed for the smaller dextrans in the experiments (see main text). The stationary state is reached faster in the approximate system compared to the actual measurements in the main text, due to the much smaller z-dimension. Perfect agreement between the numerical model and the analytical solution is obtained.

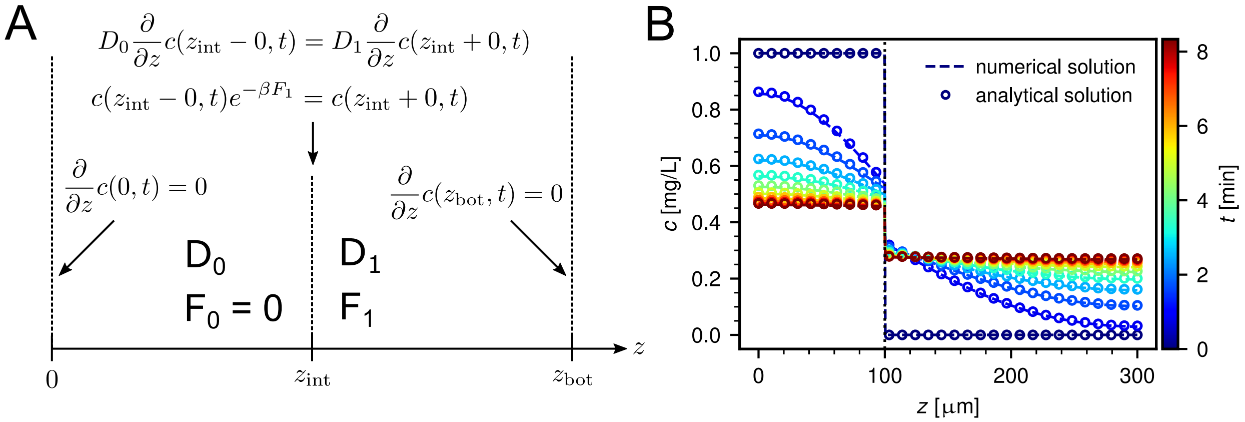

Supplementary Figure S4: Approximation of the dextran hydrogel setup as a two-segment system, which can be solved analytically. A: Two-segment system with different diffusion constants and a jump in the free-energy at the interface $z_{\rm int}$. The used boundary conditions are also indicated. B: Comparison of the numerical model and the analytical solution of the system explained in A, with values for the parameters of $z_{\rm int} = 100\ \mu$m, $z_{\rm bot} = 300\ \mu$m, $c_0 = 1$, $D_0 = 50\ \mu{\rm m}^2/s$, $D_1 = 100\ \mu{\rm m}^2/s$ and $F_1 = 0.5\ k_{\rm B}T$.

S6 Error Estimate for Numerical Analysis

In order to determine confidence intervals for the fitted parameters of D_{sol}, D_{gel} and ΔF_{gel}, the values are varied from the optimum until the agreement with the experimental data is 50% worse than for the optimal parameter values. Figure S5 shows an exemplary analysis of the fitted parameters influence on the error. All parameters are varied independently, meaning that the error is always computed while keeping all other parameters fixed at their optimal values. Also, the fitted values for d_{int} and z_{int} are not changed but kept at their optimum. It is apparent that increasing the fitted diffusion constants does not affect the agreement with the experimental data as strongly as a decrease (see Figure S5A and B). Changing the free-energy difference influences the numerical error σ more symmetrically, meaning increasing ΔF_{gel} has the same influence on the error as decreasing it.

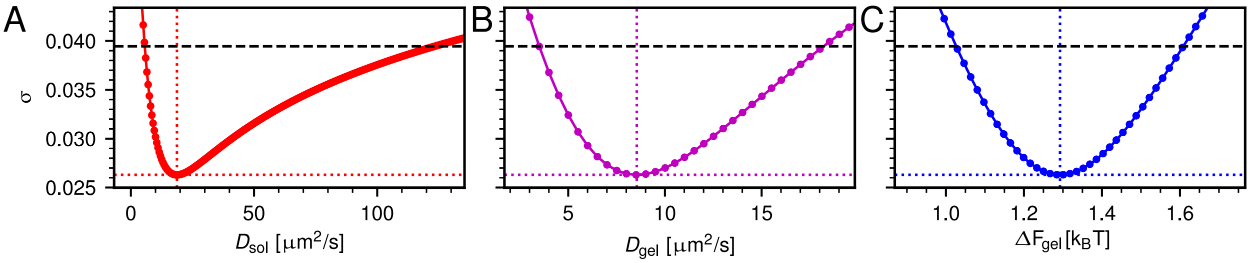

Supplementary Figure S5: Error estimation of the fitted values for D_{sol} (A), D_{gel} (B) and ΔF_{gel} (C) for measurements of $M_{\mathrm{dex}} = 40$ kDa dextran molecules diffusing into the *hPG-G10* hydrogel. Fitted optimal values for the parameters are indicated by dotted lines, while a 50% change in σ is shown by the dashed black line. A larger value of the diffusion constants does not affect the agreement with the experimental data as strongly as a smaller value.

S7 Scaling of Diffusion Constant with Dextran Size

According to the Stokes-Einstein relation, the diffusion constant of dextran molecules in the bulk solution D_{sol} is expected to scale with the dextran radius r_0 as

$$D_{\mathrm{sol}} \propto r_0^{-1}. \tag{S22}$$

Assuming the dextran polymer behaves like a freely jointed chain, its radius is related to the number of monomers N_{dex} as[4,5]

$$r_0 = b_0^{\mathrm{dex}} \sqrt{N_{\mathrm{dex}}}, \tag{S23}$$

where b_0^{dex} is the dextran monomer length and N_{dex} can be estimated from the total molecular mass of a dextran molecule M_{dex}, when the monomer mass $M_{\mathrm{dex}}^{\mathrm{mono}}$ is known

$$N_{\mathrm{dex}} = \frac{M_{\mathrm{dex}}}{M_{\mathrm{dex}}^{\mathrm{mono}}}. \tag{S24}$$

This leads to the following equality

$$D_{\mathrm{sol}} = \frac{k_{\mathrm{B}}T}{6\pi\eta_{\mathrm{w}} \frac{b_0^{\mathrm{dex}}}{\sqrt{M_{\mathrm{dex}}^{\mathrm{mono}}}} \sqrt{M_{\mathrm{dex}}}}, \tag{S25}$$

which gives rise to a scaling of $D_{\mathrm{sol}} \propto M_{\mathrm{dex}}^{-1/2}$.

S8 Expression for the Elastic Deformation Free Energy

The free-energy cost for stretching a polymer chain from an initial equilibrium mean square end-to-end distance $\langle \vec{R}_0^2 \rangle$ to a larger end-to-end distance $\langle \vec{R}^2 \rangle$ can be written as[4,5]

$$\Delta F_{\mathrm{stretch}} = \frac{3}{2} k_{\mathrm{B}} T \frac{\langle \vec{R}^2 \rangle - \langle \vec{R}_0^2 \rangle}{\langle \vec{R}_0^2 \rangle}, \tag{S26}$$

where $k_{\mathrm{B}}T$ denotes the thermal energy. In the case of the PEG linkers we define the z-component of the squared end-to-end distance as $l^2 := \langle R_z^2 \rangle$, so that $l_0^2 := \langle R_{0,z}^2 \rangle$. The PEG polymer chain is only stretched in the z-direction, thus eq. (S26) reduces to

$$\Delta F_{\mathrm{stretch}} = \frac{3}{2} k_{\mathrm{B}} T \frac{\langle R_x^2 \rangle + \langle R_y^2 \rangle + l^2 - \langle R_{0,x}^2 \rangle - \langle R_{0,y}^2 \rangle - l_0^2}{\langle \vec{R}_0^2 \rangle} = \frac{3}{2} k_{\mathrm{B}} T \frac{l^2 - l_0^2}{\langle \vec{R}_0^2 \rangle}, \tag{S27}$$

since $\langle R_x^2 \rangle = \langle R_{0,x}^2 \rangle$ and $\langle R_y^2 \rangle = \langle R_{0,y}^2 \rangle$. As the PEG polymer chain performs a random walk in all three spatial dimensions, all components of the mean squared end-to-end distance contribute equally and so

$$\frac{\langle \vec{R}_0^2 \rangle}{3} = \langle R_{0,x}^2 \rangle = \langle R_{0,y}^2 \rangle = \langle R_{0,z}^2 \rangle = l_0^2. \tag{S28}$$

Together with eq. (S27), eq. (S28) leads to the stretching free-energy for a single PEG linker polymer chain

$$\Delta F_{\mathrm{stretch}} = \frac{1}{2} k_{\mathrm{B}} T \left[\frac{l^2}{l_0^2} - 1 \right]. \tag{S29}$$

For the compression of a polymer chain from an initially larger mean square end-to-end distance $\langle \vec{R}_0^2 \rangle$ to a smaller one $\langle \vec{R}^2 \rangle$ we write[4,5]

$$\Delta F_{\mathrm{compress}} = \frac{3}{2} k_{\mathrm{B}} T \frac{\langle \vec{R}_0^2 \rangle - \langle \vec{R}^2 \rangle}{\langle \vec{R}^2 \rangle}. \tag{S30}$$

In the same way as above, we only allow compression along the z-axis, which leads to

$$\Delta F_{\text{compress}} = \frac{3}{2} k_{\text{B}} T \frac{\langle R_{0,x}^2 \rangle + \langle R_{0,y}^2 \rangle + l_0^2 - \langle R_x^2 \rangle - \langle R_y^2 \rangle - l^2}{\langle R_x^2 \rangle + \langle R_y^2 \rangle + l^2} = \frac{3}{2} k_{\text{B}} T \frac{l_0^2 - l^2}{\langle R_{0,x}^2 \rangle + \langle R_{0,y}^2 \rangle + l^2}. \tag{S31}$$

Using eq. (S28) to substitute the x- and y-components of the equilibrium end-to-end distance gives the expression for the compression free-energy of a single PEG linker

$$\Delta F_{\text{compress}} = \frac{1}{2} k_{\text{B}} T \frac{3l_0^2 - 3l^2}{2l_0^2 + l^2}. \tag{S32}$$

The total elastic deformation free-energy per PEG linker is the sum of eq. (S29) and eq. (S32)

$$\Delta F_{\text{PEG}} = \frac{1}{2} k_{\text{B}} T \left(\left[\frac{l}{l_0} \right]^2 + \frac{l_0^2 - 4l^2}{2l_0^2 + l^2} \right). \tag{S33}$$

For the twelve PEG linkers of the hydrogel unit cell this leads to eq. (10) of the main text.

S9 Estimating PEG-Monomer Hydration Number

Based on eq. (7) and eq. (12) from the main text, we obtain the following relation between the partition coefficient K_{gel} and the volume accessible to the dextran diffusors V_{free}

$$K_{\text{gel}} = \frac{V_{\text{free}}}{V_{\text{unit}}} e^{-\beta(\Delta F_{\text{dex}} + \Delta F_{\text{PEG}})}. \tag{S34}$$

The volume inaccessible to the dextran molecules V_{ex} (see eq. (8) of the main text) is composed of a part occupied by the gel or dextran directly and a part due to tightly bound hydration water, so that

$$V_{\text{ex}} = V_{\text{unit}} - V_{\text{free}} = V_{\text{gel}} + V_{\text{hyd}} + V_{\text{dex}}, \tag{S35}$$

where $V_{\text{gel}} = V_{\text{PEG}} + V_{\text{hPG}}$ denotes the excluded volume due to both gel components, $V_{\text{dex}} = 3\pi r^2 l - \frac{32}{3}\pi r^3$ (with r and l as explained in Figure 6A in the main text) denotes the excluded volume due to dextran, $V_{\text{unit}} = l^3$ is the unit cell volume and V_{hyd} is the volume occupied by hydration water. Since r denotes the hydrodynamic radius of the spherical dextran, we assume V_{hyd} to be the volume of hydration water only binding to the gel components.

The mass fraction Φ_{gel} of the gel components inside the hydrogel is defined as the ratio of the mass of the gel components m_{gel} to the total mass m_{tot}, but since the mass density of the gel components is comparable to that of water, it also represents the fraction of inaccessible volume due to the gel components

$$\Phi_{\text{gel}} := \frac{m_{\text{gel}}}{m_{\text{tot}}} \approx \frac{V_{\text{gel}}}{V_{\text{unit}}}. \tag{S36}$$

In the same fashion we can also estimate the fraction of inaccessible volume due to only the PEG linkers as

$$\Phi_{\text{PEG}} = \frac{m_{\text{PEG}}}{m_{\text{tot}}} = \frac{n_{\text{PEG}} M_{\text{PEG}}}{m_{\text{tot}}} \frac{V_{\text{app}}}{V_{\text{gel}}^{\text{sol}}} \approx \frac{V_{\text{PEG}}}{V_{\text{unit}}}, \tag{S37}$$

where we use the values for the number n_{PEG} and the molar mass M_{PEG} of the PEG linkers given in Table 1 of the Methods Section. The factor of $V_{\mathrm{app}}/V_{\mathrm{gel}}^{\mathrm{sol}}$ accounts for the fact that only $V_{\mathrm{app}} = 1\ \mu\mathrm{L}$ of the total volume of prepared gel solution $V_{\mathrm{gel}}^{\mathrm{sol}}$ are actually placed on the gel spot for the experiments. The total mass of the hydrogel m_{tot} is estimated from the measured hydrogel volumes by using the water mass density (cf. also Section S2). Combining eq. (S34), eq. (S35), and eq. (S36) from above gives the following expression for volume fraction occupied by hydration water

$$\frac{V_{\mathrm{hyd}}}{V_{\mathrm{unit}}} = 1 - \Phi_{\mathrm{gel}} - K_{\mathrm{gel}} e^{\beta(\Delta F_{\mathrm{dex}} + \Delta F_{\mathrm{PEG}})} - 3\pi \left(\frac{r}{l}\right)^2 + \frac{32}{3}\left(\frac{r}{l}\right)^3. \tag{S38}$$

The hydration water of eq. (S38) in principle binds to the entire hydrogel, meaning the hPG hubs and the PEG linkers. For a rough estimate, we neglect the presence of the hPG hubs and assume V_{hyd} is the volume of water molecules hydrating only the PEG linkers. We can compute the fraction of hydration water per unit PEG volume from eq. (S37) and eq. (S38) as

$$\frac{V_{\mathrm{hyd}}}{V_{\mathrm{PEG}}} = \frac{n_{\mathrm{hyd}} v_{\mathrm{w}}}{n_{\mathrm{PEG}} v_{\mathrm{PEG}}} = \frac{1 - \Phi_{\mathrm{gel}} - K_{\mathrm{gel}} e^{\beta(\Delta F_{\mathrm{dex}} + \Delta F_{\mathrm{PEG}})} - 3\pi \left(\frac{r}{l}\right)^2 + \frac{32}{3}\left(\frac{r}{l}\right)^3}{\Phi_{\mathrm{PEG}}}, \tag{S39}$$

where n_{hyd} is the number of hydration water molecules, v_{w} is their partial volume, n_{PEG} is the number of PEG linkers and v_{PEG} is the PEG linker partial volume. The ratio between the partial volumes of water and the PEG linkers is approximated by the ratio of their molar masses as

$$\frac{v_{\mathrm{w}}}{v_{\mathrm{PEG}}} \approx \frac{M_{\mathrm{w}}}{M_{\mathrm{PEG}}}, \tag{S40}$$

with the water molar mass $M_{\mathrm{w}} = 18$ g/mol and the molar mass of the respective PEG linker M_{PEG} (see Methods Section). From eq. (S39), we can now compute the number of hydration waters per PEG linker molecule as

$$\frac{n_{\mathrm{hyd}}}{n_{\mathrm{PEG}}} = \frac{1 - \Phi_{\mathrm{gel}} - K_{\mathrm{gel}} e^{\beta(\Delta F_{\mathrm{dex}} + \Delta F_{\mathrm{PEG}})} - 3\pi \left(\frac{r}{l}\right)^2 + \frac{32}{3}\left(\frac{r}{l}\right)^3}{\Phi_{\mathrm{PEG}}} \frac{M_{\mathrm{PEG}}}{M_{\mathrm{w}}}. \tag{S41}$$

In order to obtain the number of hydration waters per PEG-monomer we simply divide eq. (S41) by the respective number of PEG-monomers per linker N_{PEG}, which we obtain from the ratio of total linker mass M_{PEG} and PEG-monomer mass $M_{\mathrm{PEG}}^{\mathrm{mono}} = 44$ g/mol, as $N_{\mathrm{PEG}} = M_{\mathrm{PEG}}/M_{\mathrm{PEG}}^{\mathrm{mono}}$. The number of hydration waters per PEG-monomer can thus be obtained as

$$\frac{n_{\mathrm{hyd}}}{n_{\mathrm{PEG}}^{\mathrm{mono}}} = \frac{1 - \Phi_{\mathrm{gel}} - K_{\mathrm{gel}} e^{\beta(\Delta F_{\mathrm{dex}} + \Delta F_{\mathrm{PEG}})} - 3\pi \left(\frac{r}{l}\right)^2 + \frac{32}{3}\left(\frac{r}{l}\right)^3}{\Phi_{\mathrm{PEG}}} \frac{M_{\mathrm{PEG}}^{\mathrm{mono}}}{M_{\mathrm{w}}}. \tag{S42}$$

With the values of $K_{\mathrm{gel}} e^{\beta(\Delta F_{\mathrm{dex}} + \Delta F_{\mathrm{PEG}})}$, $V_{\mathrm{dex}}/V_{\mathrm{unit}} = 3\pi \left(\frac{r}{l}\right)^2 - \frac{32}{3}\left(\frac{r}{l}\right)^3$, Φ_{gel} and Φ_{PEG} for the two hydrogels from the main text, eq. (S42) allows us to estimate the number of hydration waters per PEG monomer for all measurements. Figure S6 shows the results of the calculation for each of the two hydrogels. Estimated values scatter around 8 water molecules per PEG monomer, with a slight dependence on the PEG linker length and dextran mass. Depending on the employed experimental method, values reported in the literature vary, ranging from 2 to 11 water molecules per PEG monomer.[6–10] Additionally, an increase of the hydration waters per monomer has been observed, as a function of the polymerization degree.[11] The values obtained from our estimates, all lie within the range of values reported in the literature, as indicated by the grey shaded area in Figure S6. This further corroborates our methodology and specifically the model for the free-energy of eq. (12), since the estimate of eq. (S42) is based on this model.

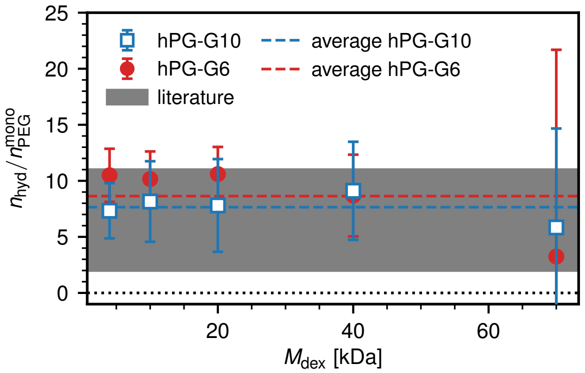

Supplementary Figure S6: Estimated number of water molecules per PEG monomer $n_{\mathrm{hyd}}/n_{\mathrm{PEG}}^{\mathrm{mono}}$ from the obtained values of K_{gel} for the two hydrogels based on eq. (S42). The estimated values scatter around $n_{\mathrm{hyd}}/n_{\mathrm{PEG}}^{\mathrm{mono}} = 8$ and agree with the range of values reported in the literature indicated as the grey shaded area, ranging from $n_{\mathrm{hyd}}/n_{\mathrm{PEG}}^{\mathrm{mono}} = 2$ to $n_{\mathrm{hyd}}/n_{\mathrm{PEG}}^{\mathrm{mono}} = 11$.[6–10]

S10 Permeability Coefficient

The definition of the permeability coefficient P is, as stated in the main text[12]

$$P(z_1, z_2) := \frac{J}{c(z_1) - c(z_2)}, \tag{S43}$$

with the stationary flux J and the equilibrium concentrations on both sides of the barrier $c(z_1)$ and $c(z_2)$. From the generalized diffusion equation (6) in the main text, one obtains the stationary flux for the case of $\partial c(z,t)/\partial t = 0$ as

$$J = D(z) e^{-\beta F(z)} \frac{\partial}{\partial z} \left(c(z,t) e^{\beta F(z)} \right), \tag{S44}$$

as a function of the diffusion constant $D(z)$ and the free-energy landscape $F(z)$ across the barrier. After rearranging eq. (S44) and integrating from one side of the barrier from z_1 to the other z_2, we obtain the following relation

$$J \int_{z_1}^{z_2} \frac{e^{\beta F(z)}}{D(z)}\, dz = c(z_1,t) e^{\beta F(z_1)} - c(z_2,t) e^{\beta F(z_2)}. \tag{S45}$$

We now assume that the free-energy value is the same on both sides of the barrier and additionally set it to zero as reference so that $F(z_1) = F(z_2) = 0$ and thus

$$J \int_{z_1}^{z_2} \frac{e^{\beta F(z)}}{D(z)}\, dz = c(z_1,t) - c(z_2,t), \tag{S46}$$

which, in combination with eq. (S43), gives eq. (14) used in the main text

$$\frac{1}{P} = \int_{z_1}^{z_2} \frac{e^{\beta F(z)}}{D(z)}\, dz. \tag{S47}$$

References

(1) Widengren, J.; Rigler, R.; Mets, Ü. Triplet-state monitoring by fluorescence correlation spectroscopy. *Journal of Fluorescence* **1994**, *4*, 255–258, DOI: 10.1007/BF01878460.

(2) Rigler, R.; Elson, E. S. *Fluorescence Correlation Spectroscopy: Theory and Applications*; Springer Series in Chemical Physics; Springer Berlin Heidelberg, 2012.

(3) Rüttinger, S.; Buschmann, V.; Krämer, B.; Erdmann, R.; MacDonald, R.; Koberling, F. Comparison and accuracy of methods to determine the confocal volume for quantitative fluorescence correlation spectroscopy. *Journal of Microscopy* **2008**, *232*, 343–352, DOI: 10.1111/j.1365-2818.2008.02105.x.

(4) Rubinstein, M.; Colby, R. H. *Polymer Physics*; OUP Oxford, 2003.

(5) Netz, R. R.; Andelman, D. Neutral and charged polymers at interfaces. *Physics Reports* **2003**, *380*, 1–95, DOI: 10.1016/S0370-1573(03)00118-2.

(6) Huang, L.; Nishinari, K. Interaction between poly(ethylene glycol) and water as studied by differential scanning calorimetry. *Journal of Polymer Science, Part B: Polymer Physics* **2001**, *39*, 496–506, DOI: 10.1002/1099-0488(20010301)39:5<496::AID-POLB1023>3.0.CO;2-H.

(7) Shikata, T.; Takahashi, R.; Sakamoto, A. Hydration of Poly(ethylene oxide)s in Aqueous Solution As Studied by Dielectric Relaxation Measurements. *The Journal of Physical Chemistry B* **2006**, *110*, 8941–8945, DOI: 10.1021/jp060356i.

(8) Kaatze, U.; Gottmann, O.; Podbielski, R.; Pottel, R.; Terveer, U. Dielectric relaxation in aqueous solutions of some oxygen-containing linear hydrocarbon polymers. *The Journal of Physical Chemistry* **1978**, *82*, 112–120, DOI: 10.1021/j100490a025.

(9) Bieze, T. W. N.; Barnes, A. C.; Huige, C. J. M.; Enderby, J. E.; Leyte, J. C. Distribution

of Water around Poly(ethylene oxide): A Neutron Diffraction Study. *The Journal of Physical Chemistry* **1994**, *98*, 6568–6576, DOI: 10.1021/j100077a024.

(10) Żwirbla, W.; Sikorska, A.; Linde, B. B. Ultrasonic investigations of water mixtures with polyethylene glycols 200, 400 and ethylene glycol. *Journal of Molecular Structure* **2005**, *743*, 49–52, DOI: 10.1016/j.molstruc.2005.02.019.

(11) Branca, C.; Magazù, S.; Maisano, G.; Migliardo, F.; Migliardo, P.; Romeo, G. Hydration Study of PEG/Water Mixtures by Quasi Elastic Light Scattering, Acoustic and Rheological Measurements. *The Journal of Physical Chemistry B* **2002**, *106*, 10272–10276, DOI: 10.1021/jp014345v.

(12) Diamond, J. M.; Katz, Y. Interpretation of nonelectrolyte partition coefficients between dimyristoyl lecithin and water. *The Journal of Membrane Biology* **1974**, *17*, 121–154, DOI: 10.1007/BF01870176.
